# PD-L1-Guided Chemo-Immunotherapy in Advanced Triple-Negative Breast Cancer: A Meta-Analysis of Survival Benefits and Toxicity Profiles

**DOI:** 10.3390/cancers18091352

**Published:** 2026-04-23

**Authors:** Lingshan Nan, Xi Zuo, Xiaohui Yin, Haiming Li, Yue Wang, Xiaomin Wang, Dong Chen, Ganlin Zhang

**Affiliations:** 1 Department of Oncology, Beijing Hospital of Traditional Chinese Medicine, Capital Medical University, Beijing 100010, China; 2 School of Graduates, Beijing University of Chinese Medicine, Beijing 100029, China; 3 Jiangsu Collaborative Innovation Center of Traditional Chinese Medicine Prevention and Treatment of Tumor, The First Clinical Medical College, Nanjing University of Chinese Medicine, Nanjing 210046, China; 4 Department of Surgery, Guang’anmen Hospital, China Academy of Chinese Medical Sciences, Beijing 100053, China

**Keywords:** triple-negative breast cancer, immune checkpoint inhibitors, chemotherapy, meta-analysis

## Abstract

Importance: Triple-negative breast cancer (TNBC) is characterized by high tumor mutation burden and frequent programmed cell death ligand 1 (PD-L1) expression, making immune checkpoint inhibitors (ICIs) a promising therapeutic approach. However, randomized trials of chemoimmunotherapy (Chemo-IO) in locally recurrent unresectable or metastatic TNBC have shown inconsistent results, necessitating a clearer understanding of efficacy and patient selection. Objective: The aim of this study was to evaluate the efficacy and safety of chemotherapy combined with immunotherapy vs. chemotherapy alone in patients with locally recurrent unresectable or metastatic triple-negative breast cancer and to identify beneficiary populations to guide optimal treatment selection. Data Sources: PubMed, Embase, and the Cochrane Library were searched from database inception through 23 August 2025. Study Selection: Randomized clinical trials (RCTs) comparing chemotherapy combined with ICIs vs. chemotherapy with placebo or control in patients with locally recurrent unresectable or metastatic TNBC were selected. Data Extraction and Synthesis: Two investigators independently performed data extraction and assessed risk of bias using the Cochrane Risk of Bias 2 tool (RoB 2). Heterogeneity was evaluated using the I^2^ statistic. Data were synthesized using random-effects meta-analysis models to calculate hazard ratios (HRs) for time-to-event outcomes and risk ratios (RRs) for dichotomous outcomes according to the Preferred Reporting Items for Systematic Reviews and Meta-analyses (PRISMA) reporting guidelines. Results: Seven RCTs comprising 3485 patients (2085 in the Chemo-IO group, 1400 in the control group) were included. The median age across trials ranged from 52 to 57 years. Chemo-IO significantly improved PFS (HR, 0.82 [95% CI, 0.76–0.89]; *p* < 0.01) and OS (HR = 0.88; 95% CI: 0.81–0.96; *p* = 0.004) in the intention-to-treat (ITT) population, with PFS benefit particularly evident in PD-L1-positive patients (HR = 0.68, 95% CI: 0.59–0.79). However, OS improvement in the PD-L1-positive subgroup was not statistically significant. CBR did not differ significantly in the intention-to-treat population (RR, 1.11 [95% CI, 0.99–1.25]; *p* =  0.08) but was higher in PD-L1-positive patients (RR, 1.15 [95% CI, 1.01–1.31]; *p* = 0.04). Safety analyses revealed no significant differences in overall AE (RR, 1.01 [95% CI, 0.99–1.02]; *p* = 0.35), TEAE (RR, 1.01 [95% CI, 0.99–1.03]; *p* = 0.19), or grade ≥ 3 TEAE (RR, 1.00; [95% CI, 0.93–1.07]; *p* =  0.98). However, serious AE (RR, 1.32 [95% CI, 1.11–1.57]; *p* = 0.001) and irAE (RR, 1.86 [95% CI, 1.41–2.45]; *p* <  0.01) were more frequent with Chemo-IO. Conclusions and Relevance: Chemotherapy combined with immunotherapy significantly improved PFS and OS in patients with locally recurrent unresectable or metastatic TNBC, without substantially increasing chemotherapy-related toxicities. However, the OS benefit in PD-L1-positive patients was not statistically significant, and the combined regimen was associated with higher rates of serious and immune-related adverse events. These findings support the use of Chemo-IO as a treatment option, highlighting the importance of PD-L1 status and careful monitoring of immune-mediated toxicities in clinical practice.

## Simple Summary

Triple-negative breast cancer (TNBC) remains a clinically challenging subtype of breast cancer, and the role of immune checkpoint inhibitors combined with chemotherapy is still debated because randomized trials have produced inconsistent results. In this systematic review and meta-analysis of seven randomized trials, chemoimmunotherapy improved progression-free survival in the overall population and more clearly in PD-L1-positive disease, whereas overall survival improved modestly in the intention-to-treat population but not significantly in the PD-L1-positive subgroup. The combination did not clearly increase the overall incidence of treatment-emergent toxicity, but serious and immune-related adverse events were more frequent. These findings support a selective rather than universal use of chemoimmunotherapy in advanced TNBC and highlight the need for assay-harmonized biomarkers, steroid-sparing chemotherapy backbones, and close monitoring of immune-related toxicities.

## 1. Introduction

Triple-negative breast cancer (TNBC) accounts for approximately 15% to 20% of all breast cancers [1] and is characterized by aggressive clinical behavior, high rates of recurrence and metastasis, and a generally poor prognosis. Historically, surgery and chemotherapy constituted the standard first-line treatment for the majority of patients [2]. Despite these interventions, however, recurrence or visceral metastasis occurred in approximately 63% of patients. Outcomes following recurrence were dismal: the median overall survival (mOS) was limited to 9 to 12 months [3], and the 5-year survival rate remained at approximately 13% [3]. Nevertheless, the distinct biology of TNBC—including genomic instability, tumor-infiltrating lymphocytes, and elevated programmed death-ligand 1 (PD-L1) expression—provides a rationale for incorporating immunotherapy [4].

In recent years, chemoimmunotherapy (Chemo-IO) emerged as a novel therapeutic strategy for metastatic triple-negative breast cancer (mTNBC). The pivotal IMpassion130 [5] and KEYNOTE-355 [6] trials have validated the efficacy of immunotherapy combinations. In IMpassion130, atezolizumab plus nab-paclitaxel significantly prolonged mPFS (7.2 vs. 5.0 months; HR, 0.80; *p* < 0.01), particularly in the PD-L1 IC-positive subgroup. Similarly, KEYNOTE-355 demonstrated that pembrolizumab plus chemotherapy significantly extended both mPFS (9.7 vs. 5.6 months; HR, 0.65; *p* < 0.01) and mOS (23.0 vs. 16.1 months; HR, 0.73; *p* < 0.05) in PD-L1 CPS ≥ 10 patients. However, subsequent trials—specifically IMpassion131 [7] and IMpassion132 [8]—failed to confirm these benefits, demonstrating no statistically significant improvement. Neither study demonstrated statistically significant improvements in mPFS or mOS within the intention-to-treat (ITT) populations or PD-L1-positive subgroups of patients with mTNBC or early relapse. These conflicting data led to the withdrawal of the US indication for atezolizumab in metastatic TNBC. This inconsistency highlights the influence of variables such as chemotherapy backbones, PD-L1 assay methodologies, and patient selection. To address these inconsistencies, this study synthesized data from recent high-quality randomized controlled trials (RCTs). The objective was to evaluate efficacy disparities between Chemo-IO and chemotherapy alone (Chemo), identify optimal regimens and beneficiary subgroups, and establish robust evidence to guide clinical and health-economic decision-making.

## 2. Methods

This study was designed and conducted in strict accordance with the Preferred Reporting Items for Systematic Reviews and Meta-Analyses (PRISMA) guidelines [9], and the completed PRISMA checklist is provided in Supplementary File S1. The study protocol was registered in PROSPERO under the registration number CRD420251132609.

### 2.1. Search Strategies and Research Options

To comprehensively evaluate the efficacy and safety of Chemo-IO in locally recurrent unresectable/metastatic TNBC, we systematically searched PubMed, Embase, and the Cochrane Library from database inception to 23 August 2025. The search strategy combined Medical Subject Headings (MeSHs) and free-text terms for “triple-negative breast cancer,” “immune checkpoint inhibitors (ICIs),” “PD-L1,” “chemotherapy,” and “randomized controlled trial.” To ensure data completeness, we prioritized the most recent and comprehensive reports for trials published multiple times and manually reviewed reference lists of included studies. When full-text access was unavailable or data were insufficient, we contacted corresponding authors directly.

Two investigators independently screened titles and abstracts, followed by full-text review. Discrepancies were resolved by consensus or by a third senior investigator. Only studies published in English were included. Discrepancies were resolved through consensus or arbitration by a third senior investigator. Eligible studies enrolled patients with histologically or cytologically confirmed locally recurrent unresectable, metastatic, or early-relapsing advanced TNBC and compared chemotherapy plus ICIs with chemotherapy plus placebo or control. The investigational arm received Chemo-IO, whereas the control arm received Chemo. Primary outcomes were PFS, OS, and immune-related adverse events (irAEs). Secondary endpoints included objective response rate (ORR), clinical benefit rate (CBR), general adverse events (AEs), treatment-emergent adverse events (TEAEs), and serious adverse events (SAEs).

### 2.2. Data Extraction and Quality Assessment

Two investigators independently extracted data using a pre-defined, standardized form. Collected variables encompassed study characteristics (design, sample size, and geographic region), patient demographics (age, Eastern Cooperative Oncology Group (ECOG) performance status, PD-L1 expression, and prior treatment history), and efficacy outcomes, including PFS, OS, ORR, and CBR. To accurately evaluate the safety profile, we specifically analyzed adverse events with a plausible causal relationship to the study treatment. Data were cross-verified for accuracy, and any discrepancies were resolved through consensus. The risk of bias was assessed using the Cochrane Risk of Bias 2 (RoB 2) tool. This assessment covered the randomization process, deviations from intended interventions, missing outcome data, outcome measurement, and selection of the reported results. Each domain was categorized as “low risk,” “high risk,” or “some concerns”.

### 2.3. Statistical Analysis

Statistical analyses were performed using Stata 14.0 (Stata Corp, College Station, Texas, United States) and Rev Man 5.4 (Cochrane Collaboration, London, United Kingdom). For time-to-event outcomes (PFS and OS), we extracted hazard ratios (HRs) with 95% confidence intervals (CIs) from the published reports. Where HRs were not reported, estimates were derived from Kaplan–Meier curves or event data using the method described by Tierney and colleagues [10]. Binary outcomes, including ORR, CBR, and AEs, were analyzed using risk ratios (RRs) [11]. Pre-specified subgroup analyses were conducted based on PD-L1 status, age, race, ECOG performance status, metastatic status, and prior chemotherapy history. Heterogeneity was assessed using the Q statistic and I^2^ values. Significant heterogeneity, defined as a Q-statistic *p* < 0.10 or I^2^ > 50%, prompted the use of a random-effects model; otherwise, a fixed-effects model was applied [12]. Sources of heterogeneity and result robustness were further investigated using L’Abbe plots, Galbraith radial plots, and sensitivity analyses. In instances of clinical or methodological heterogeneity, random-effects meta-regression was employed using the Paule–Mandel method to estimate between-study variance (τ^2^), with adjustments applied via the Hartung–Knapp–Sidik–Jonkman method. Publication bias was evaluated using funnel plots, Egger’s and Begg’s tests, and the trim-and-fill method. All statistical tests were two-sided, with significance defined as *p* < 0.05.

## 3. Result

**Figure 1:**
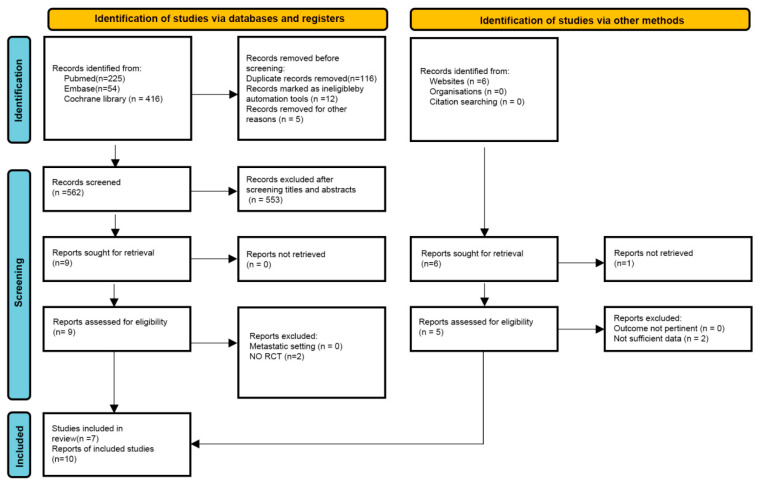
Preferred Reporting Items for Systematic Reviews and Meta-Analyses (PRISMA) flowchart for study selection.

The literature search identified 695 records from PubMed (*n* = 225), Embase (*n* = 54), and the Cochrane Library (*n* = 416) (Table S1). Following deduplication and preliminary screening, 14 studies underwent full-text review. Ultimately, seven randomized controlled trials comprising 10 distinct publications met the eligibility criteria and were included in the meta-analysis (Figure 1). The included trials were ALICE [13], IMpassion130 [5], IMpassion131 [7], IMpassion132 [8], KEYNOTE-355 [6], TBCRC 043 [14], and TORCHLIGHT [15]; multiple publications were utilized for the IMpassion130, IMpassion131, and KEYNOTE-355 trials. Regarding risk of bias, six studies were classified as having a low risk across all domains. The TBCRC 043 trial was the sole exception, rated as having “some concerns” regarding deviations from the intended intervention. No study demonstrated a serious or critical risk of bias (Figure S1).

### 3.1. Eligible Studies and Characteristics

Seven RCTs comprising five Phase III and two Phase II studies published between 2018 and 2024 satisfied the inclusion criteria. These trials evaluated the efficacy and safety of Chemo-IO versus Chemo for the treatment of locally recurrent unresectable/metastatic TNBC. The cumulative cohort included 3485 patients, with 2085 assigned to the investigational arm and 1400 to the control arm. Individual study sample sizes (intention-to-treat or full analysis set) ranged from 68 to 902 cases: IMpassion130 (*n* = 902), KEYNOTE-355 (*n* = 847), IMpassion131 (*n* = 651), TORCHLIGHT (*n* = 531), IMpassion132 (*n* = 380), TBCRC 043 (*n* = 106), and ALICE (*n* = 68). The mean age across the cohort ranged from 52 to 57 years, and the median follow-up duration spanned 8.8 to 44.1 months. All studies employed randomized (1:1 or 2:1 ratios), double-blind, placebo-controlled designs and were predominantly international or multicenter in scope. Eligibility criteria consistently mandated an ECOG performance status of 0–1, and most protocols excluded patients with prior chemotherapy or targeted therapy for metastatic disease. Stratification factors typically included PD-L1 expression status (IC ≥ 1% or CPS ≥ 10), age, ethnicity, metastatic status, prior treatment history, and geographic region. Investigational arms administered PD-L1 (atezolizumab) or PD-1 (pembrolizumab, toripalimab) inhibitors combined with chemotherapy (nab-paclitaxel, paclitaxel, anthracyclines, or carboplatin), whereas control arms received chemotherapy with or without placebo. Detailed baseline clinical characteristics are provided in Table 1 and Tables S2–S6.

### 3.2. Progression-Free Survival

**Figure 2:**
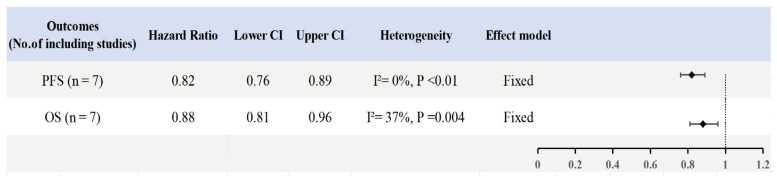
Forest plots of progression-free survival (PFS) and overall survival (OS) for Chemo-IO versus chemotherapy alone (Chemo) in the ITT population. Hazard ratios (HRs) with 95% confidence intervals (CIs) were pooled using fixed-effect or random-effects models according to between-study heterogeneity.

As shown in Figure 2, in the ITT population, Chemo-IO significantly improved PFS compared with Chemo (HR = 0.82; 95% CI: 0.76–0.89; I^2^ = 0%, *p* < 0.01). Subgroup analyses indicated that efficacy varied by PD-L1 status. A significant improvement in PFS was observed in patients in the PD-L1-positive subgroup (HR = 0.68; 95% CI: 0.59–0.79; I^2^ = 0%, *p* < 0.01), whereas no significant benefit was detected in the PD-L1-negative subgroup (HR = 0.89; 95% CI: 0.80–1.00; I^2^ = 2%, *p* = 0.06) (Figure S2). Significant benefits persisted across age groups (≤64 years: HR 0.79, 95% CI 0.71–0.87; >64 years: HR 0.77, 95% CI 0.63–0.93) and ECOG performance scores (score 0: HR 0.72, 95% CI 0.63–0.83; score 1: HR 0.85, 95% CI 0.74–0.98). Regarding race, White patients derived significant benefit (HR 0.76, 95% CI 0.66–0.88), whereas results for Asian (HR 0.83, 95% CI 0.65–1.07) and Black patients (HR 0.83, 95% CI 0.51–1.37) did not reach statistical significance. Analysis by metastatic site demonstrated significantly prolonged PFS in patients with lung (HR 0.78, 95% CI 0.67–0.91) or liver metastases (HR 0.80, 95% CI 0.66–0.98), but not in those with bone metastases (HR 0.90, 95% CI 0.74–1.10). Chemo-IO improved outcomes regardless of prior chemotherapy exposure, though the benefit was more pronounced in chemotherapy-naive patients (HR 0.69, 95% CI 0.58–0.81) compared with those who had received prior treatment (HR 0.86, 95% CI 0.76–0.98) (Table S7 and Figure S3). Egger’s test, Begg’s test, and funnel plot assessments suggested potential publication bias solely within the age subgroup analysis; however, the results remained robust following trim-and-fill adjustment (Tables S8–S13 and Figure S4). No significant publication bias was detected for other outcomes (Table S14 and Figures S5 and S6).

### 3.3. Overall Survival

In the ITT population, Chemo-IO significantly improved OS compared with Chemo (HR = 0.88; 95% CI: 0.81–0.96; I^2^ = 37%, *p* = 0.004) (Figure 2). Subgroup analyses based on PD-L1 status revealed a trend toward survival benefit in PD-L1-positive patients, although this did not reach statistical significance (HR = 0.82; 95% CI: 0.64–1.04; I^2^ = 70%, *p* = 0.11); sensitivity analysis confirmed the robustness of this finding. Conversely, no significant benefit was observed in the PD-L1-negative subgroup (HR = 1.01; 95% CI: 0.89–1.15; I^2^ = 0%, *p* = 0.85) (Figure S7). Regarding age, patients aged <65 years derived significant benefit (HR = 0.83; 95% CI: 0.73–0.95), whereas those aged ≥65 years did not (HR = 0.95; 95% CI: 0.72–1.25). Analysis by performance status demonstrated significantly improved OS in patients with an ECOG score of 0 (HR = 0.80; 95% CI: 0.68–0.95), but not in those with a score of 1 (HR = 0.93; 95% CI: 0.71–1.24); this conclusion was also supported by sensitivity analysis. Furthermore, patients with lung and/or liver metastases exhibited significantly prolonged overall survival (HR = 0.87; 95% CI: 0.76–0.99) (Table S15 and Figure S8). Assessments of heterogeneity using Galbraith radial plots and sensitivity analyses indicated robust results (Figures S9–S11), and meta-regression analyses based on sample size and publication year are presented in Figure S12. Finally, Egger’s test, Begg’s test, and funnel plot assessments revealed no evidence of significant publication bias (Table S16 and Figure S13).

### 3.4. Objective Response Rate and Clinical Benefit Rate

**Figure 3:**
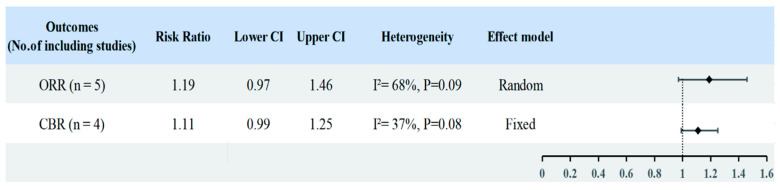
Forest plots of objective response rate (ORR) and clinical benefit rate (CBR) for Chemo-IO versus chemotherapy alone (Chemo) in the ITT population. Risk ratios (RRs) with 95% confidence intervals (CIs) were pooled using fixed-effect or random-effects models according to between-study heterogeneity.

In the ITT population, Chemo-IO and Chemo did not significantly improve the ORR (RR = 1.19; 95% CI: 0.97–1.46; I^2^ = 68%, *p* = 0.09) (Figure 3). Sensitivity analyses confirmed the robustness of this conclusion. Similarly, no significant improvement was observed in the PD-L1-positive subgroup (RR = 1.10; 95% CI: 0.97–1.25; I^2^ = 34%, *p* = 0.13) (Table S17 and Figure S14). We assessed heterogeneity using L’Abbe and Galbraith radial plots, with sensitivity analyses confirming the stability of the results (Figures S15–S17). Meta-regression analyses based on sample size and publication year are presented in Figure S18. Regarding the CBR, meta-analysis of the ITT population showed no significant benefit with Chemo-IO (RR = 1.11; 95% CI: 0.99–1.25; I^2^ = 37%, *p* = 0.08). However, in the PD-L1-positive subgroup, Chemo-IO demonstrated a significant CBR (RR = 1.15; 95% CI: 1.01–1.31; I^2^ = 0%, *p* = 0.04) (Table S18 and Figure S19). No evidence of publication bias was detected for these outcomes based on Egger’s test, Begg’s test, and funnel plot assessments (Tables S19 and S20, Figures S20 and S21).

### 3.5. Safety Analysis

**Figure 4:**
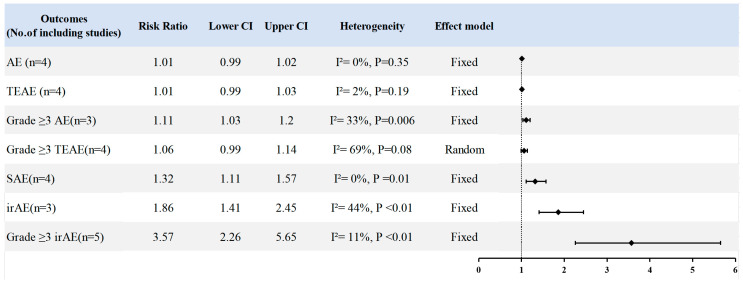
Forest plots of adverse event outcomes for Chemo-IO versus chemotherapy alone (Chemo) in the ITT population. Risk ratios (RRs) with 95% confidence intervals (CIs) were pooled using fixed-effect or random-effects models according to between-study heterogeneity.

As illustrated in Figure 4, the safety analysis revealed no significant differences between the treatment groups regarding the overall incidence of adverse events (RR = 1.01; 95% CI: 0.99–1.02; I^2^ = 0%; *p* = 0.35), TEAEs (RR = 1.01; 95% CI: 0.99–1.03; I^2^ = 7%; *p* = 0.19), or Grade ≥ 3 TEAEs (RR = 1.00; 95% CI: 0.93–1.07; I^2^ = 68%; *p* = 0.98). Conversely, Chemo-IO was associated with a significantly higher incidence of Grade ≥ 3AEs (RR = 1.11; 95% CI: 1.03–1.20; *p* = 0.006), SAEs (RR = 1.32; 95% CI: 1.11–1.57; *p* = 0.001), irAEs (RR = 1.86; 95% CI: 1.41–2.45; *p* < 0.01), and Grade ≥ 3irAEs (RR = 3.57; 95% CI: 2.26–5.65; *p* < 0.01) (Table S21 and Figure S22). We evaluated heterogeneity using L’Abbé and Galbraith radial plots, and sensitivity analyses confirmed the robustness of these findings (Figures S23–S25). Meta-regression analyses based on sample size and publication year are presented in Figure S26. Assessment via Egger’s test, Begg’s test, and funnel plots indicated no evidence of publication bias (Table S22 and Figure S27).

We further analyzed the incidence of specific adverse events across the safety population. Regarding general adverse events, we observed no significant differences between the Chemo-IO and Chemo arms in the rates of rash, fatigue, weakness, constipation, vomiting, nausea, anemia, elevated alanine aminotransferase, or decreased neutrophil count (Figure S28). In terms of TEAEs, patients in the Chemo-IO group experienced significantly higher rates of nausea (RR = 1.80; 95% CI: 1.23–2.64; *p* = 0.002) and neutropenia (RR = 1.12; 95% CI: 1.01–1.25; *p* = 0.03). Conversely, the incidences of fatigue, anemia, and alopecia were comparable to those observed in the Chemo group (Figures S29 and S30). Analysis of irAEs revealed significantly increased risks in the Chemo-IO arm for hypothyroidism (RR = 3.20; 95% CI: 2.46–4.17; *p* < 0.01), hyperthyroidism (RR = 6.23; 95% CI: 3.41–11.39; *p* < 0.01), pneumonia (RR = 4.47; 95% CI: 2.28–8.76; *p* < 0.01), and immune-related rash (RR = 1.22; 95% CI: 1.07–1.39; *p* = 0.004). However, no significant differences were detected regarding fever, infusion-related reactions, colitis, pancreatitis, immune-related diabetes, hepatitis, or adrenal insufficiency (Figures S31 and S32).

## 4. Discussion

Although ICIs have transformed the treatment landscape for many cancers, their efficacy in TNBC has been inconsistent. Biologically, TNBC is characterized by high levels of tumour-infiltrating lymphocytes, PD-L1 expression, and tumor mutational burden, which are putative predictors of response to immunotherapy [16]. However, pivotal phase 3 trials of Chemo-IO in metastatic triple-negative breast cancer have reported conflicting efficacy results, creating uncertainty in clinical practice. To resolve this ambiguity and inform clinical practice, we conducted a systematic review and meta-analysis of RCTs.

Our study is broadly consistent with prior meta-analyses published in 2023–2025, which showed a reproducible PFS benefit for PD-1/PD-L1 blockade in advanced or metastatic TNBC but less consistent conclusions regarding OS, particularly in PD-L1-defined populations. Compared with these earlier syntheses, the present analysis incorporates newer randomized evidence and updated follow-up, separates ITT estimates from PD-L1-positive subgroup estimates more explicitly, and provides a more detailed evaluation of safety and clinically relevant exploratory subgroups. These updates reinforce that PFS benefit is more robust than OS benefit and that assay, regimen, and population heterogeneity remain central to interpretation.

Our study demonstrated that Chemo-IO improved PFS in the ITT population and more clearly in PD-L1-positive disease, whereas OS improved modestly in the ITT population but not significantly in the PD-L1-positive subgroup, and the survival benefit was modulated by factors such as treatment history, disease burden, and ECOG performance status. Specifically, patients with PD-L1-positive tumors derived substantial PFS benefit and improved CBR, with a manageable safety profile. These findings underscore that optimizing Chemo-IO requires rigorous patient selection based on validated PD-L1 assays, judicious choice of chemotherapy backbone, and vigilant monitoring for irAEs.

Regarding survival outcomes, Chemo-IO significantly improved PFS in patients with locally recurrent unresectable/metastatic TNBC, a benefit primarily driven by PD-L1 positivity1. In the ITT population, Chemo-IO also significantly improved OS. However, in the PD-L1-positive subgroup, the improvement in OS was not statistically significant. Although the HR in the PD-L1-positive subgroup (0.82) suggested a survival advantage, the 95% CI crossed unity (0.64–1.04), indicating statistical uncertainty. Furthermore, high statistical heterogeneity (I^2^ = 70%) limited the certainty of this finding. This heterogeneity probably stems from methodological differences across trials, including the use of different PD-L1 assays, scoring algorithms, and tissue sampling sites. For instance, the KEYNOTE-355 trial [6] employed the Dako 22C3 PD-L1 IHC assay, defining positivity as a CPS ≥10. Conversely, the IMpassion trials [5,7,8] utilized the VENTANA PD-L1 SP142 IHC assay, defining positivity as IC ≥1%. Although both the 22C3 and SP263 assays held European approval and CE-IVD certification for CPS testing [17], their clinical application remained distinct: a CPS ≥ 10 identified candidates for pembrolizumab [18], whereas the SP142 assay (IC ≥ 1%) constituted the sole approved eligibility criterion for atezolizumab [19]. Therefore, the choice of assay, platform, and scoring algorithm can significantly influence patient stratification and trial outcomes. Evidence suggests [20] that while the Dako and Ventana platforms were technically reliable, the CPS and IC scoring systems assessed distinct cellular populations, which precluded their interchangeability in mTNBC [21,22]. Furthermore, PD-L1 expression can vary between primary tumours and metastatic sites. These factors dictated that the selection of specific ICIs necessitated strict adherence to the corresponding validated detection methods, platforms, and scoring thresholds.

Chemotherapy induced immunogenic cell death (ICD) within the tumor microenvironment, a process that released antigens and danger-associated molecular patterns (DAMPs) [23]. This process remodels the tumor microenvironment and activates antitumor immunity, thereby potentiating the response to ICIs [24]. To evaluate whether specific chemotherapy backbones differentially impacted efficacy, the seven RCTs included in this study employed a diverse array of agents, including paclitaxel, nab-paclitaxel, anthracyclines, platinum-gemcitabine, and capecitabine. Among these, anthracyclines were considered particularly immunogenic, capable of triggering the release of four distinct DAMPs [25]. Indeed, in the adjuvant TNBC setting, the KEYNOTE-522 [26], GeparNuevo [27], and IMpassion031 [28] trials confirmed that anthracycline-containing regimens significantly enhanced response rates to ICIs. However, the utility of conventional anthracyclines is limited by cumulative and irreversible cardiotoxicity. The ALICE trial [13] addressed this limitation, demonstrating a significant PFS benefit with pegylated liposomal doxorubicin (PLD) combined with cyclophosphamide and atezolizumab in mTNBC. Yet, despite the potential for long-term use of PLD, its prohibitive cost relative to other anthracyclines presented a substantial barrier to widespread adoption [29]. Consequently, taxanes remain a cornerstone of treatment for mTNBC.

Notably, outcomes differed markedly between trials using the same ICI. While IMpassion130 [5] established a significant PFS benefit for atezolizumab plus nab-paclitaxel in both the overall and PD-L1-positive populations, IMpassion131 [7] failed to demonstrate any benefit for atezolizumab combined with paclitaxel in PD-L1-positive patients. Given comparable baseline characteristics, the discrepancy implicates the obligatory corticosteroid premedication used with paclitaxel, which might attenuate immunotherapy efficacy [30]. Although subgroup analyses of KEYNOTE-355 [6] suggested PFS benefits with pembrolizumab regardless of whether paclitaxel or nab-paclitaxel was used, these findings were limited by small sample sizes and imbalances in patient populations and PD-L1 testing methods. Mechanistically, corticosteroids exerted immunosuppressive effects through the multifaceted regulation of T-cell activation, differentiation, and migration [31]. While widely utilized to manage irAEs induced by ICIs [32] and to prevent chemotherapy-associated hypersensitivity, their impact on survival appeared context-dependent. Administration for severe immune-related adverse events does not seem to compromise OS [32,33]. Conversely, the use of steroids for disease-related symptoms or chemotherapy prophylaxis necessitated extreme caution [34]. A meta-analysis corroborated this risk, revealing that patients treated with steroids for non-AEs indications exhibited significantly higher risks of death and disease progression, including a 50% increase in mortality risk among those with brain metastases [35]. Therefore, in locally recurrent unresectable/metastatic TNBC, minimizing the dose and duration of prophylactic corticosteroids is imperative. Specifically, chemotherapy backbones requiring high-dose corticosteroid premedication should be avoided to preserve immunotherapy efficacy.

Subgroup analyses revealed differential PFS benefit according to PD-L1 status, site of metastasis, and prior treatment history. Notably, significant PFS improvement was confined to patients with PD-L1-positive tumors, with no discernible benefit in PD-L1-negative patients. This outcome stood in contrast to findings from early-stage TNBC studies—specifically KEYNOTE-522 [26] and IMpassion031 [28]—in which neoadjuvant ICIs combined with chemotherapy improved disease-free survival (DFS) independent of PD-L1 expression. This discrepancy might be attributable to the higher immunogenicity of early-stage tumors [36]. Furthermore, PFS benefit was observed in patients with lung or liver metastases, indicating regimen activity despite high disease burden. Similarly, the KEYNOTE-189 trial [37] demonstrated that pembrolizumab combined with chemotherapy significantly prolonged both OS and PFS in metastatic non-small-cell lung cancer; notably, this efficacy was maintained independent of tumor burden. In our study, subgroup analyses indicated that significant OS benefits were associated with younger age, good performance status, and the presence of visceral metastases. These findings suggest that younger, fit patients can benefit from chemoimmunotherapy even with high visceral disease burden. Regarding prior treatment, although PFS benefit was observed irrespective of previous chemotherapy, it was most pronounced in chemotherapy-naive patients. This observation aligned with reports of higher immunogenicity in untreated metastatic disease [16,38]. In contrast, the IMpassion132 trial [8], which enrolled patients with locally recurrent unresectable/metastatic TNBC, reported rapid recurrence following multi-agent systemic therapy and found no prognostic improvement with the addition of PD-L1 inhibitors. Although KEYNOTE-355 [6] reported contrasting results in the PD-L1-positive population, these findings derived from an exploratory subgroup analysis with a limited sample size, precluding definitive comparison.

Furthermore, divergent eligibility criteria regarding the disease-free interval (DFI) were noted: IMpassion132 [8] calculated DFI from the date of the last chemotherapy dose or surgery, whereas KEYNOTE-355 [6] excluded patients with a DFI of less than 6 months. Although current approvals do not exclude patients with early-relapsing disease, available data question the therapeutic value of chemoimmunotherapy in this cohort. These observations underscore that, in the metastatic setting, prior treatment history is a prognostic factor as important as PD-L1 status. Consequently, future trials are needed to compare different chemotherapy backbones combined with immunotherapy. Additionally, investigation into de-escalation strategies, such as induction-followed-by-maintenance or shortened combination cycles, was deemed necessary for elderly patients or those with compromised performance status. Such approaches were intended to facilitate more personalized Chemo-IO regimens for locally recurrent unresectable/metastatic TNBC.

Regarding safety profiles, the Chemo-IO group showed no significant difference compared with the Chemo group in the incidence of AEs, TEAEs, or Grade ≥ 3 TEAEs. This suggests that adding immunotherapy does not markedly exacerbate the toxicity profile of chemotherapy, consistent with previous reports in solid tumors and likely due to overlapping toxicities [39,40]. Conversely, the Chemo-IO group had significantly higher rates of serious adverse events and grade 3 or worse adverse events, largely attributable to irAEs. Notably, the risk of grade 3 or worse irAEs was markedly elevated with Chemo-IO. We attribute this increase to the inherent toxicity of ICIs and cumulative effects from prolonged exposure. Mechanistically, while priming antitumour immunity, these agents can trigger aberrant systemic immune activation, leading to collateral tissue damage [41]. The 2022 ESMO guidelines on immunotherapy toxicity management highlighted that, distinct from chemotherapy-induced adverse effects, irAEs were characterized by an insidious onset, multi-organ involvement, and significant variability in severity. Although specific high-grade irAEs, such as immune-mediated pneumonitis, occurred with low incidence, they carried a disproportionately high mortality risk. This factor likely drove the increased incidence of SAEs observed in the Chemo-IO cohort. Regarding age-specific outcomes, our study did not show a clear clinical benefit for elderly patients. This diverges from observations in most other solid tumors, where the risk–benefit profile of ICIs often favours older patients [42]. Therefore, based on the efficacy and safety profiles observed, chemoimmunotherapy is best reserved for patients with good performance status. The safety profile is manageable only with appropriate patient selection, baseline organ assessment, and active surveillance for endocrine, pulmonary, dermatologic, and other immune-mediated toxicities. For elderly patients or those with poor performance status, alternative strategies, such as limiting the duration of the combination regimen, warranted consideration.

## 5. Limitations

Our study has several limitations. First, follow-up duration and maturity of OS data varied across trials. Second, major clinical and methodological heterogeneity arose from differences in PD-L1 assays, scoring algorithms, and tissue compartments, which precluded harmonized analyses by precise PD-L1 expression level. Third, subgroup analyses by age, race, metastatic site, and prior treatment were exploratory and based on limited study-level data; they should be considered hypothesis-generating. Fourth, the small number of included studies limited the reliability of publication bias testing and constrained our ability to perform stable subgroup analyses by ICI type or assay methodology. Finally, because chemotherapy backbones differed substantially across studies, backbone-specific conclusions remain provisional.

## 6. Conclusions

This meta-analysis shows that chemoimmunotherapy improves PFS and modestly improves OS in the overall population with locally recurrent unresectable or metastatic TNBC, with the clearest efficacy signal being the PFS benefit in PD-L1-positive disease. Because OS in the PD-L1-positive subgroup was not statistically significant and serious and immune-related toxicities were increased, treatment selection should integrate validated PD-L1 testing, chemotherapy backbones, prior treatment history, performance status, and careful toxicity monitoring. Consequently, our data support pembrolizumab plus chemotherapy as a standard of care for treatment-naive patients with a combined positive score of 10 or more. Highly immunogenic regimens (e.g., nab-paclitaxel) should be prioritised. In cases of resistance, gemcitabine plus platinum (carboplatin or cisplatin) represents an appropriate alternative. Conversely, paclitaxel protocols requiring high-dose corticosteroids, as well as anthracycline-based regimens, were deemed unsuitable due to their potential to blunt the immune response. Furthermore, minimizing the dosage and duration of prophylactic corticosteroids, alongside rigorous vigilance for ICI-specific irAEs and cumulative toxicities, remained paramount.

## Figures and Tables

**Table 1:** Main Characteristics of the Studies Included in the Systematic Review.

Clinical Trial	Year	Sample Size	Study Design	Experimental Group	Control Group	Primary End Point
IMpassion130	2018/2020/2021	ITT: 902 Chemo-IO: 451; Chemo: 451	Phase III international, randomized, double-blind, placebo-controlled trial (randomisation 1:1)	Atezolizumab + nab-paclitaxel	Placebo + nab-paclitaxel	PFS, OS, ORR, DOR, AEs
KEYNOTE-355	2020/2022	ITT: 847 Chemo-IO: 566; Chemo: 281	Phase III international, randomized, double-blind, placebo-controlled trial (randomisation 2:1)	Pembrolizumab + chemotherapy	Placebo + chemotherapy	PFS, OS, ORR, DOR, DCR, AEs
IMpassion131	2021	ITT: 651 Chemo-IO: 431; Chemo: 220	Phase III international, randomized, double-blind, placebo-controlled trial (randomisation 2:1)	Atezolizumab + paclitaxel	Placebo + paclitaxel	PFS, OS, ORR, EORTC QLQ-C30, GHS/HRQoL, AEs
ALICE	2022	FAS: 68 Chemo-IO: 40; Chemo: 28	Phase IIb multicenter, randomized, double-blind, parallel-group, placebo-controlled trial (randomisation 3:2)	Atezolizumab + anthracycline-based chemotherapy	Placebo + anthracycline-based chemotherapy	PFS, OS, ORR, DOR, DRR, CBR, PROs, AEs
TBCRC 043	2024	ALL: 106 Chemo-IO: 56; Chemo: 50	Phase II prospective, multicenter, randomized clinical trial (randomisation 1:1)	Atezolizumab + Carboplatin	Carboplatin	PFS, OS, ORR, DOR; CBR, AEs
TORCHLIGHT	2024	ITT: 531 Chemo-IO: 353; Chemo: 178	Phase III multicenter, randomized, double-blind, parallel-group, placebo-controlled trial (randomisation 2:1)	Toripalimab + nab-paclitaxel	Placebo + nab-paclitaxel	PFS, OS, ORR, DCR, DOR, CBR, AEs
IMpassion132	2024	mITT: 380 Chemo-IO: 188; Chemo: 192	Phase III international, randomized, double-blind, placebo-controlled trial (randomisation 1:1)	Atezolizumab + chemotherapy	Placebo + chemotherapy	PFS, OS, ORR, DOR, CBR, AEs, 12-month and 18-month survival rates

## Data Availability

The data supporting this study were derived from publicly accessible resources. All datasets analyzed were sourced from PubMed, Web of Science, Embase, and the Cochrane Library.

## Supplementary Materials

The following supporting information can be downloaded at https://www.mdpi.com/article/10.3390/cancers18091352/s1, Supplementary File S1: PRISMA_2020_checklist; Supplementary Table S1: Search strategy; Supplementary Table S2: Main Characteristics of the 7 studies included in meta-analysis; Supplementary Table S3: Race distribution of 7 studies included in meta-analysis; Supplementary Table S4: Baseline Metastatic status of 7 studies included in meta-analysis; Supplementary Table S5: Baseline previous therapy of 7 studies included in meta-analysis; Supplementary Table S6: Treatment exposure of 7 studies included in meta-analysis; Supplementary Table S7: Subgroup analyses on progression-free survival based on age, race, and other baseline characteristics. Data are presented as hazard ratios (HR) with 95% confidence intervals (CI) for each subgroup; Table S8: The result of PFS in the age ≤ 64 years subgroup of patients with chemoimmunotherapy vs. chemotherapy in mTNBC before the trim and fill procedure; Supplementary Table S9: Trimming estimator of the result of PFS in the age ≤ 64 years subgroup of patients with chemoimmunotherapy vs. chemotherapy in mTNBC; Supplementary Table S10: The result of PFS in the age ≤ 64 years subgroup of patients with chemoimmunotherapy vs. chemotherapy in mTNBC after the trim and fill procedure; Supplementary Table S11: The result of PFS in the age > 64 years subgroup of patients with chemoimmunotherapy vs. chemotherapy in mTNBC before the trim and fill procedure; Supplementary Table S12: Trimming estimator of the result of PFS in the age > 64 years subgroup of patients with chemoimmunotherapy vs. chemotherapy in mTNBC; Supplementary Table S13: The result of PFS in the age > 64 years subgroup of patients with chemoimmunotherapy vs. chemotherapy in mTNBC after the trim and fill procedure; Supplementary Table S14: The Outcomes of Begg’s test and Egger’s test for PFS in the ITT Population and Subgroups; Supplementary; Supplementary Table S15: Subgroup analyses on overall survival based on age, race, and other baseline characteristics. Data are presented as hazard ratios (HR) with 95% confidence intervals (CI) for each subgroup; Supplementary Table S16: The Outcomes of Begg’s test and Egger’s test for OS in the ITT Population and Subgroups; Supplementary Table S17: Subgroup analyses on objective response rate. Data are presented as risk ratios (RR) with 95% confidence intervals (CI) for each subgroup; Supplementary Table S18: Subgroup analyses on Clinical Benefit Rate. Data are presented as risk ratios (RR) with 95% confidence intervals (CI) for each subgroup; Supplementary Table S19: The Outcomes of Begg’s test and Egger’s test for ORR in the ITT Population and Subgroups; Supplementary Table S20: The Outcomes of Begg’s test and Egger’s test for CBR in the ITT Population and Subgroups; Supplementary Table S21: Overall analysis on safety endpoints. Data are presented as risk ratios (RR) with 95% confidence intervals (CI); Supplementary Table S22: The Outcomes of Begg’s test and Egger’s test for safety analysis in the ITT Population; Supplementary Figure S1: Bias Risk Assessment of Included Clinical Studies; Supplementary Figure S2: Forest plots of PFS for chemoimmunotherapy vs. chemotherapy in mTNBC; Supplementary Figure S3: Forest plots of PFS for chemoimmunotherapy vs. chemotherapy in mTNBC by subgroup; Supplementary Figure S4: The funnel plots of PFS for chemoimmunotherapy vs. chemotherapy in mTNBC by age subgroup after the trim and fill procedure; Supplementary Figure S5: The funnel plots of PFS for chemoimmunotherapy vs. chemotherapy in mTNBC (part 1); Supplementary Figure S6: The funnel plots of PFS for chemoimmunotherapy vs. chemotherapy in mTNBC (part 2); Supplementary Figure S7: Forest plots of OS for chemoimmunotherapy vs. chemotherapy in mTNBC; Supplementary Figure S8: Forest plots of OS for chemoimmunotherapy vs. chemotherapy in mTNBC by subgroup; Supplementary Figure S9: The Galbraith radial plot of OS for chemoimmunotherapy vs. chemotherapy in mTNBC; Supplementary Figure S10: The sensitivity analysis of OS for chemoimmunotherapy vs. chemotherapy in mTNBC; Supplementary Figure S11: The outcome of sensitivity analysis of OS for chemoimmunotherapy vs. chemotherapy in mTNBC; Supplementary Figure S12: The meta-regressions of OS for chemoimmunotherapy vs. chemotherapy in mTNBC according to publication year and sample size; Supplementary Figure S13: The funnel plots of OS for chemoimmunotherapy vs. chemotherapy in mTNBC; Supplementary Figure S14: Forest plots of ORR for chemoimmunotherapy vs. chemotherapy in mTNBC; Supplementary Figure S15: The L’Abbe plot and Galbraith radial plot of ORR for chemoimmunotherapy vs. chemotherapy in mTNBC; Supplementary Figure S16: The sensitivity analysis of ORR for chemoimmunotherapy vs. chemotherapy in mTNBC; Supplementary Figure S17: The outcome of sensitivity analysis of ORR for chemoimmunotherapy vs. chemotherapy in mTNBC; Supplementary Figure S18: The meta-regressions of ORR for chemoimmunotherapy vs. chemotherapy in mTNBC according to publication year and sample size; Supplementary Figure S19: Forest plots of CBR for chemoimmunotherapy vs. chemotherapy in mTNBC; Supplementary Figure S20: The funnel plots of ORR for chemoimmunotherapy vs. chemotherapy in mTNBC; Supplementary Figure S21: The funnel plots of CBR for chemoimmunotherapy vs. chemotherapy in mTNBC; Supplementary Figure S22: Forest plots of safety outcomes for chemoimmunotherapy vs. chemotherapy in mTNBC; Supplementary Figure S23: The L’Abbe plot and Galbraith radial plot of safety outcomes for chemoimmunotherapy vs. chemotherapy in mTNBC; Supplementary Figure S24: The sensitivity analysis of safety outcomes for chemoimmunotherapy vs. chemotherapy in mTNBC; Supplementary Figure S25: The outcome of sensitivity analysis of safety outcomes for chemoimmunotherapy vs. chemotherapy in mTNBC; Supplementary Figure S26: The meta-regressions of safety outcomes for chemoimmunotherapy vs. chemotherapy in mTNBC according to publication year and sample size; Supplementary Figure S27: The funnel plots of safety outcomes for chemoimmunotherapy vs. chemotherapy in mTNBC; Supplementary Figure S28: Forest plots of the incidence of adverse events for chemoimmunotherapy versus chemotherapy in mTNBC; Supplementary Figure S29: Forest plots of the incidence of treatment-emergent adverse events for chemoimmunotherapy versus chemotherapy in mTNBC; Supplementary Figure S30: Forest plots of the incidence of treatment-emergent adverse events for chemoimmunotherapy versus chemotherapy in mTNBC, adjusted for heterogeneity; Supplementary Figure S31: Forest plots of the incidence of immune-related adverse events for chemoimmunotherapy versus chemotherapy in mTNBC; Supplementary Figure S32: Forest plots of the incidence of treatment-emergent adverse events for chemoimmunotherapy versus chemotherapy in mTNBC, adjusted for heterogeneity.

## Abbreviations

TNBC, triple-negative breast cancer; Chemo-IO, chemotherapy combined with immunotherapy; ICI, immune checkpoint inhibitor; PD-L1, programmed cell death ligand 1; OS, overall survival; PFS, progression-free survival; CBR, clinical benefit rate; irAE, immune-related adverse event; TEAE, treatment-emergent adverse event; AE, adverse event; HR, hazard ratio; RR, risk ratio; CI, confidence interval; RCT, randomized clinical trial; PRISMA, Preferred Reporting Items for Systematic Reviews and Meta-Analyses; RoB 2, Cochrane Risk of Bias 2 tool.
