# Supplementary material for: PD-L1-Guided Chemo-Immunotherapy in Advanced Triple-Negative Breast Cancer: A Meta-Analysis of Survival Benefits and Toxicity Profiles
Source: Cancers (Basel). 2026 Apr 23;18(9):1352. doi: 10.3390/cancers18091352 (PMC13162691; doi:10.3390/cancers18091352)

CONTENTS

CONTENTS 1

Supplementary Table S1: Search strategy 4

Supplementary Table S2. Main Characteristics of the 7 studies included in meta-analysis 10

Supplementary Table S3: Race distribution of 7 studies included in meta-analysis........ 15

Supplementary Table S4: Baseline Metastatic status of 7 studies included in meta-analysis........ 16

Supplementary Table S5: Baseline previous therapy of 7 studies included in meta-analysis........ 17

Supplementary Table S6. Treatment exposure of 7 studies included in meta-analysis 18

Supplementary Table S7. Subgroup analyses on progression-free survival based on age, race, and other baseline characteristics. Data are presented as hazard ratios (HR) with 95% confidence intervals (CI) for each subgroup........ 20

Supplementary Table S8. The result of PFS in the age ≤64 years subgroup of patients with chemoimmunotherapy vs. chemotherapy in mTNBC before the trim and fill procedure. 21

Supplementary Table S9. Trimming estimator of the result of PFS in the age ≤64 years subgroup of patients with chemoimmunotherapy vs. chemotherapy in mTNBC. 22

Supplementary Table S10. The result of PFS in the age ≤64 years subgroup of patients with chemoimmunotherapy vs. chemotherapy in mTNBC after the trim and fill procedure........ 23

Supplementary Table S11. The result of PFS in the age＞64 years subgroup of patients with chemoimmunotherapy vs. chemotherapy in mTNBC before the trim and fill procedure. 24

Supplementary Table S12. Trimming estimator of the result of PFS in the age＞64 years subgroup of patients with chemoimmunotherapy vs. chemotherapy in mTNBC. 25

Supplementary Table S13. The result of PFS in the age＞64 years subgroup of patients with chemoimmunotherapy vs. chemotherapy in mTNBC after the trim and fill procedure........ 26

Supplementary Table S14. The Outcomes of Begg's test and Egger's test for PFS in the ITT Population and Subgroups. ... 27

Supplementary Table S15. Subgroup analyses on overall survival based on age, race, and other baseline characteristics. Data are presented as hazard ratios (HR) with 95% confidence intervals (CI) for each subgroup. 28

Supplementary Table S16. The Outcomes of Begg's test and Egger's test for OS in the ITT Population and Subgroups...... 29

Supplementary Table S17. Subgroup analyses on objective response rate . Data are presented as risk ratios (RR) with 95% confidence intervals (CI) for each subgroup. 30

Supplementary Table S18. Subgroup analyses on Clinical Benefit Rate. Data are presented as risk ratios (RR) with 95% confidence intervals (CI) for each subgroup. 31

Supplementary Table S19 The Outcomes of Begg's test and Egger's test for ORR in the ITT Population and Subgroups. .. 32

Supplementary Table S20. The Outcomes of Begg's test and Egger's test for CBR in the ITT Population and Subgroups... 33

Supplementary Table S21. Overall analysis on safety endpoints. Data are presented as risk ratios (RR) with 95% confidence intervals (CI). 34

Supplementary Table S22 The Outcomes of Begg's test and Egger's test for safety analysis in the ITT Population. 35

Supplementary Figure S1 Bias Risk Assessment of Included Clinical Studies.() 36

Supplementary Figure S2 Forest plots of PFS for chemoimmunotherapy vs. chemotherapy in mTNBC 37

Supplementary Figure S3 Forest plots of PFS for chemoimmunotherapy vs. chemotherapy in mTNBC by subgroup. 38

Supplementary Figure S4 The funnel plots of PFS for chemoimmunotherapy vs. chemotherapy in mTNBC by age subgroup after the trim and fill procedure. 39

Supplementary Figure S5 The funnel plots of PFS for chemoimmunotherapy vs. chemotherapy in mTNBC (part 1) 40

Supplementary Figure S6 The funnel plots of PFS for chemoimmunotherapy vs. chemotherapy in mTNBC(part 2) 41

Supplementary Figure S7 Forest plots of OS for chemoimmunotherapy vs. chemotherapy in mTNBC. 42

Supplementary Figure S8 Forest plots of OS for chemoimmunotherapy vs. chemotherapy in mTNBC by subgroup. 43

Supplementary Figure S9 The Galbraith radial plot of OS for chemoimmunotherapy vs. chemotherapy in mTNBC. 44

Supplementary Figure S10 The sensitivity analysis of OS for chemoimmunotherapy vs. chemotherapy in mTNBC. 45

Supplementary Figure S11 The outcome of sensitivity analysis of OS for chemoimmunotherapy vs. chemotherapy in mTNBC. 46

Supplementary Figure S12 The meta-regressions of OS for chemoimmunotherapy vs. chemotherapy in mTNBC according to publication year and sample size. 47

Supplementary Figure S13 The funnel plots of OS for chemoimmunotherapy vs. chemotherapy in mTNBC. 48

Supplementary Figure S14 Forest plots of ORR for chemoimmunotherapy vs. chemotherapy in mTNBC 49

Supplementary Figure S15 The L'Abbe plot and Galbraith radial plot of ORR for chemoimmunotherapy vs. chemotherapy in mTNBC. 50

Supplementary Figure S16 The sensitivity analysis of ORR for chemoimmunotherapy vs. chemotherapy in mTNBC. 51

Supplementary Figure S17 The outcome of sensitivity analysis of ORR for chemoimmunotherapy vs. chemotherapy in mTNBC. 52

Supplementary Figure S18 The meta-regressions of ORR for chemoimmunotherapy vs. chemotherapy in mTNBC according to publication year and sample size. 53

Supplementary Figure S19 Forest plots of CBR for chemoimmunotherapy vs. chemotherapy in mTNBC. 54

Supplementary Figure S20 The funnel plots of ORR for chemoimmunotherapy vs. chemotherapy in mTNBC.......... 55

Supplementary Figure S21 The funnel plots of CBR for chemoimmunotherapy vs. chemotherapy in mTNBC. 56

Supplementary Figure S22 Forest plots of safety outcomes for chemoimmunotherapy vs. chemotherapy in mTNBC. 57

Supplementary Figure S23 The L'Abbe plot and Galbraith radial plot of safety outcomes for chemoimmunotherapy vs. chemotherapy in mTNBC.......... 58

Supplementary Figure S24 The sensitivity analysis of safety outcomes for chemoimmunotherapy vs. chemotherapy in mTNBC. 59

Supplementary Figure S25 The outcome of sensitivity analysis of safety outcomes for chemoimmunotherapy vs. chemotherapy in mTNBC.......... 60

Supplementary Figure S26 The meta-regressions of safety outcomes for chemoimmunotherapy vs. chemotherapy in mTNBC according to publication year and sample size. 61

Supplementary Figure S27 The funnel plots of safety outcomes for chemoimmunotherapy vs. chemotherapy in mTNBC. . 62

Supplementary Figure S28 Forest plots of the incidence of adverse events for chemoimmunotherapy versus chemotherapy in mTNBC. 63

Supplementary Figure S29 Forest plots of the incidence of treatment-emergent adverse events for chemoimmunotherapy versus chemotherapy in mTNBC. 64

Supplementary Figure S30 Forest plots of the incidence of treatment-emergent adverse events for chemoimmunotherapy versus chemotherapy in mTNBC, adjusted for heterogeneity. 65

Supplementary Figure S31 Forest plots of the incidence of immune-related adverse events for chemoimmunotherapy versus chemotherapy in mTNBC.......... 66

Supplementary Figure S32 Forest plots of the incidence of treatment-emergent adverse events for chemoimmunotherapy versus chemotherapy in mTNBC, adjusted for heterogeneity. 67

Supplementary Table S1: Search strategy

Database	Search strategy
Pubmed	**#1** "Triple Negative Breast Neoplasms"[Mesh]<br>**#2** ((ER-Negative PR-Negative HER2-Negative Breast Cancer[Title/Abstract]) OR (ER Negative PR Negative HER2 Negative Breast Cancer[Title/Abstract]) OR (ER-Negative PR-Negative HER2-Negative Breast Neoplasms[Title/Abstract]) OR (ER Negative PR Negative HER2 Negative Breast Neoplasms[Title/Abstract]) OR (Triple Negative Breast Cancer[Title/Abstract]) OR (Triple-Negative Breast Cancer[Title/Abstract]) OR (Breast Cancers, Triple-Negative[Title/Abstract]) OR (Breast Cancer, Triple-Negative[Title/Abstract]) OR (Triple-Negative Breast Cancers[Title/Abstract]) OR (Triple-Negative Breast Neoplasm[Title/Abstract]) OR (Breast Neoplasms, Triple-Negative[Title/Abstract]) OR (Breast Neoplasm, Triple-Negative[Title/Abstract]) OR (Triple Negative Breast Neoplasm[Title/Abstract]) OR (Triple-Negative Breast Neoplasms[Title/Abstract]))<br>**#3 #1 OR #2**<br>**#4** "Consolidation Chemotherapy"[Mesh]<br>**#5** ((Chemotherapies, Consolidation[Title/Abstract]) OR (Chemotherapy, Consolidation[Title/Abstract]) OR (Consolidation Chemotherapies[Title/Abstract]) OR ("Maintenance Chemotherapy"[Mesh]) OR (Chemotherapies, Maintenance[Title/Abstract]) OR (Chemotherapy, Maintenance[Title/Abstract]) OR (Maintenance Chemotherapies[Title/Abstract]) OR ("Chemotherapy, Adjuvant"[Mesh]) OR (Adjuvant Chemotherapy[Title/Abstract]) OR (Adjuvant Drug Therapy[Title/Abstract]) OR (Drug Therapy, Adjuvant[Title/Abstract]) OR ("Antineoplastic Combined Chemotherapy Protocols"[Mesh]) OR (Combined Antineoplastic Agents[Title/Abstract]) OR (Antineoplastic Agents, Combined[Title/Abstract]) OR (Agent, Combined Antineoplastic[Title/Abstract]) OR (Antineoplastic Agent, Combined[Title/Abstract]) OR (Combined Antineoplastic Agent[Title/Abstract]) OR (Anticancer Drug Combinations[Title/Abstract]) OR (Anticancer Drug Combination[Title/Abstract]) OR (Drug Combination, Anticancer[Title/Abstract]) OR (Drug Combinations, Anticancer[Title/Abstract]) OR (Drug Combinations, Antineoplastic[Title/Abstract]) OR (Antineoplastic Combined Chemotherapy Regimens[Title/Abstract]) OR (Antineoplastic Drug Combinations[Title/Abstract]) OR (Antineoplastic Drug Combination[Title/Abstract]) OR (Combinations, Antineoplastic Drug[Title/Abstract]) OR (Drug Combination, Antineoplastic[Title/Abstract]) OR (Antineoplastic Chemotherapy Protocols[Title/Abstract]) OR (Antineoplastic Chemotherapy Protocol[Title/Abstract]) OR (Chemotherapy Protocol, Antineoplastic[Title/Abstract]) OR (Protocol, Antineoplastic Chemotherapy[Title/Abstract]) OR (Protocols, Antineoplastic Chemotherapy[Title/Abstract]) OR (Cancer Chemotherapy Protocols[Title/Abstract]) OR (Cancer Chemotherapy Protocol[Title/Abstract]) OR (Chemotherapy Protocol, Cancer[Title/Abstract]) OR (Chemotherapy Protocols, Cancer[Title/Abstract]) OR (Protocol, Cancer Chemotherapy[Title/Abstract]) OR (Protocols, Cancer Chemotherapy[Title/Abstract]) OR (Chemotherapy Protocols, Antineoplastic[Title/Abstract]) OR (albumin-bound paclitaxel[Title/Abstract]) OR (paclitaxel[Title/Abstract]) OR (gemcitabine[Title/Abstract]) OR

	(carboplatin[Title/Abstract]) OR (Capecitabine[Title/Abstract]) OR (Vinorelbine[Title/Abstract]) OR (Etoposide[Title/Abstract]) OR (Doxorubicin Liposome Injection[Title/Abstract]) OR (Carboplatin[Title/Abstract]) OR (Chemotherapy[Title/Abstract]) OR (Chemotherapies[Title/Abstract]))<br>**#6 #4 OR #5**<br>**#7** "Randomized Controlled Trials as Topic"[Mesh]<br>**#8** ((randomized controlled trial[Publication Type]) OR (controlled clinical trial[Publication Type]) OR (Clinical Trials, Randomized[Title/Abstract]) OR (Trials, Randomized Clinical[Title/Abstract]) OR (Controlled Clinical Trials, Randomized[Title/Abstract]) OR (Clinical Trials[Title/Abstract]))<br>**#9 #7 OR #8**<br>**#10** (("Immune Checkpoint Inhibitors"[Mesh]) OR ("Abatacept"[Mesh]) OR ("Ipilimumab"[Mesh]) OR ("Nivolumab"[Mesh]))<br>**#11** (("Immune Checkpoint Inhibitors" [Pharmacological Action]) OR (Checkpoint Inhibitors, Immune[Title/Abstract]) OR (Immune Checkpoint Blockers[Title/Abstract]) OR (Checkpoint Blockers, Immune[Title/Abstract]) OR (Immune Checkpoint Inhibitor[Title/Abstract]) OR (Checkpoint Inhibitor, Immune[Title/Abstract]) OR (CTLA-4 Inhibitors[Title/Abstract]) OR (CTLA 4 Inhibitors[Title/Abstract]) OR (Cytotoxic T-Lymphocyte-Associated Protein 4 Inhibitors[Title/Abstract]) OR (Cytotoxic T Lymphocyte Associated Protein 4 Inhibitors[Title/Abstract]) OR (Cytotoxic T-Lymphocyte-Associated Protein 4 Inhibitor[Title/Abstract]) OR (Cytotoxic T Lymphocyte Associated Protein 4 Inhibitor[Title/Abstract]) OR (CTLA-4 Inhibitor[Title/Abstract]) OR (CTLA 4 Inhibitor[Title/Abstract]) OR (PD-1 Inhibitors[Title/Abstract]) OR (PD 1 Inhibitors[Title/Abstract]) OR (Programmed Cell Death Protein 1 Inhibitor[Title/Abstract]) OR (Programmed Cell Death Protein 1 Inhibitors[Title/Abstract]) OR (PD-1 Inhibitor[Title/Abstract]) OR (Inhibitor, PD-1[Title/Abstract]) OR (PD 1 Inhibitor[Title/Abstract]) OR (Immune Checkpoint Blockade[Title/Abstract]) OR (Checkpoint Blockade, Immune[Title/Abstract]) OR (Immune Checkpoint Inhibition[Title/Abstract]) OR (Checkpoint Inhibition, Immune[Title/Abstract]) OR (PD-L1 Inhibitors[Title/Abstract]) OR (PD L1 Inhibitors[Title/Abstract]) OR (Programmed Death-Ligand 1 Inhibitors[Title/Abstract]) OR (Programmed Death Ligand 1 Inhibitors[Title/Abstract]) OR (PD-L1 Inhibitor[Title/Abstract]) OR (PD L1 Inhibitor[Title/Abstract]) OR (PD-1-PD-L1 Blockade[Title/Abstract]) OR (Blockade, PD-1-PD-L1[Title/Abstract]) OR (PD 1 PD L1 Blockade[Title/Abstract]) OR (PD-1[Title/Abstract]) OR (PD-L1[Title/Abstract]) OR ("pembrolizumab" [Supplementary Concept]) OR ("atezolizumab" [Supplementary Concept]) OR ("lisavanbulin" [Supplementary Concept]) OR ("toripalimab " [Supplementary Concept]) OR ("sotorasib" [Supplementary Concept]) OR ("spartalizumab" [Supplementary Concept]) OR ("relatlimab" [Supplementary Concept]) OR ("PHI-101" [Supplementary Concept]) OR ("quemliclustat" [Supplementary Concept]) OR ("Opdualag" [Supplementary Concept]))<br>**#12 #10 AND #11**<br>**#13 #3AND #6 AND #9 AND #12**
Embase	**#1** 'triple negative breast cancer'/exp OR 'triple negative breast cancer'

	**#2** 'ER-Negative PR-Negative HER2-Negative Breast Cancer':ab,kw,ti OR 'ER Negative PR Negative HER2 Negative Breast Cancer':ab,kw,ti OR 'ER-Negative PR-Negative HER2-Negative Breast Neoplasms':ab,kw,ti OR 'ER Negative PR Negative HER2 Negative Breast Neoplasms':ab,kw,ti OR 'Triple Negative Breast Cancer':ab,kw,ti OR 'Triple-Negative Breast Cancer':ab,kw,ti OR 'Breast Cancers, Triple-Negative':ab,kw,ti OR 'Breast Cancer, Triple-Negative':ab,kw,ti OR 'Triple-Negative Breast Cancers':ab,kw,ti OR 'Triple-Negative Breast Neoplasm':ab,kw,ti OR 'Breast Neoplasms, Triple-Negative':ab,kw,ti OR 'Breast Neoplasm, Triple-Negative':ab,kw,ti OR 'Triple Negative Breast Neoplasm':ab,kw,ti OR 'Triple-Negative Breast Neoplasms':ab,kw,ti<br>**#3 #1 OR #2**<br>**#4** 'cancer chemotherapy'/exp OR 'cancer chemotherapy'<br>**#5** 'Chemotherapy':ab,kw,ti OR 'Chemotherapies':ab,kw,ti 'anticancer chemotherapy':ab,kw,ti OR 'antineoplastic chemotherapy':ab,kw,ti OR 'carcinochemotherapy':ab,kw,ti OR 'chemotherapy, cancer':ab,kw,ti OR 'tumor chemotherapy':ab,kw,ti OR 'tumour chemotherapy':ab,kw,ti OR 'cancer chemotherapy':ab,kw,ti OR 'Consolidation Chemotherapy':ab,kw,ti OR 'Chemotherapies, Consolidation':ab,kw,ti OR 'Chemotherapy, Consolidation':ab,kw,ti OR 'Consolidation Chemotherapies':ab,kw,ti OR 'Maintenance Chemotherapy' OR 'Chemotherapies, Maintenance':ab,kw,ti OR 'Chemotherapy, Maintenance':ab,kw,ti OR 'Maintenance Chemotherapies':ab,kw,ti OR 'Antineoplastic Combined Chemotherapy Protocols':ab,kw,ti OR 'Combined Antineoplastic Agents':ab,kw,ti OR 'Antineoplastic Agents, Combined':ab,kw,ti OR 'Agent, Combined Antineoplastic':ab,kw,ti OR 'Antineoplastic Agent, Combined':ab,kw,ti OR 'Combined Antineoplastic Agent':ab,kw,ti OR 'Anticancer Drug Combinations':ab,kw,ti OR 'Anticancer Drug Combination':ab,kw,ti OR 'Drug Combination, Anticancer':ab,kw,ti OR 'Drug Combinations, Anticancer':ab,kw,ti OR 'Drug Combinations, Antineoplastic':ab,kw,ti OR 'Antineoplastic Combined Chemotherapy Regimens':ab,kw,ti OR 'Antineoplastic Drug Combinations':ab,kw,ti OR 'Antineoplastic Drug Combination':ab,kw,ti OR 'Combinations, Antineoplastic Drug':ab,kw,ti OR 'Drug Combination, Antineoplastic':ab,kw,ti OR 'Antineoplastic Chemotherapy Protocols':ab,kw,ti OR 'Antineoplastic Chemotherapy Protocol':ab,kw,ti OR 'Chemotherapy Protocol, Antineoplastic':ab,kw,ti OR 'Protocol, Antineoplastic Chemotherapy':ab,kw,ti OR 'Protocols, Antineoplastic Chemotherapy':ab,kw,ti OR 'Cancer Chemotherapy Protocols':ab,kw,ti OR 'Cancer Chemotherapy Protocol':ab,kw,ti OR 'Chemotherapy Protocol, Cancer':ab,kw,ti OR 'Chemotherapy Protocols, Cancer':ab,kw,ti OR 'Protocol, Cancer Chemotherapy':ab,kw,ti OR 'Protocols, Cancer Chemotherapy':ab,kw,ti OR 'Chemotherapy Protocols, Antineoplastic':ab,kw,ti OR 'albumin-bound paclitaxel':ab,kw,ti OR 'paclitaxel':ab,kw,ti OR 'gemcitabine':ab,kw,ti OR 'carboplatin':ab,kw,ti OR 'Capecitabine':ab,kw,ti OR 'Vinorelbine':ab,kw,ti OR 'Etoposide':ab,kw,ti OR 'Doxorubicin Liposome Injection':ab,kw,ti OR 'Carboplatin':ab,kw,ti<br>**#6 #4 OR #5**<br>**#7** 'randomized controlled trial (topic)'/exp OR 'randomized controlled trial (topic)'<br>**#8** 'Randomized Controlled Trials as Topic':ab,kw,ti OR 'randomized controlled trial':ab,kw,ti OR 'controlled clinical trial':ab,kw,ti OR 'Clinical Trials, Randomized':ab,kw,ti OR 'Trials, Randomized Clinical':ab,kw,ti OR 'Controlled Clinical Trials, Randomized':ab,kw,ti OR 'Clinical Trials':ab,kw,ti<br>**#9 #7 OR #8**

	**#10** 'immune checkpoint inhibitor'/exp OR 'immune checkpoint inhibitor'<br>**#11** 'Immune Checkpoint Inhibitors':ab,kw,ti OR 'Abatacept':ab,kw,ti OR 'Ipilimumab':ab,kw,ti OR 'Nivolumab':ab,kw,ti OR 'Immune Checkpoint Inhibitors':ab,kw,ti OR 'Checkpoint Inhibitors, Immune':ab,kw,ti OR 'Immune Checkpoint Blockers':ab,kw,ti OR 'Checkpoint Blockers, Immune':ab,kw,ti OR 'Immune Checkpoint Inhibitor':ab,kw,ti OR 'Checkpoint Inhibitor, Immune':ab,kw,ti OR 'CTLA-4 Inhibitors':ab,kw,ti OR 'CTLA 4 Inhibitors':ab,kw,ti OR 'Cytotoxic T-Lymphocyte-Associated Protein 4 Inhibitors':ab,kw,ti OR 'Cytotoxic T Lymphocyte Associated Protein 4 Inhibitors':ab,kw,ti OR 'Cytotoxic T-Lymphocyte-Associated Protein 4 Inhibitor':ab,kw,ti OR 'Cytotoxic T Lymphocyte Associated Protein 4 Inhibitor':ab,kw,ti OR 'CTLA-4 Inhibitor':ab,kw,ti OR 'CTLA 4 Inhibitor':ab,kw,ti OR 'PD-1 Inhibitors':ab,kw,ti OR 'PD 1 Inhibitors':ab,kw,ti OR 'Programmed Cell Death Protein 1 Inhibitor':ab,kw,ti OR 'Programmed Cell Death Protein 1 Inhibitors':ab,kw,ti OR 'PD-1 Inhibitor':ab,kw,ti OR 'Inhibitor, PD-1':ab,kw,ti OR 'PD 1 Inhibitor':ab,kw,ti OR 'Immune Checkpoint Blockade':ab,kw,ti OR 'Checkpoint Blockade, Immune':ab,kw,ti OR 'Immune Checkpoint Inhibition':ab,kw,ti OR 'Checkpoint Inhibition, Immune':ab,kw,ti OR 'PD-L1 Inhibitors':ab,kw,ti OR 'PD L1 Inhibitors':ab,kw,ti OR 'Programmed Death-Ligand 1 Inhibitors':ab,kw,ti OR 'Programmed Death Ligand 1 Inhibitors':ab,kw,ti OR 'PD-L1 Inhibitor':ab,kw,ti OR 'PD L1 Inhibitor':ab,kw,ti OR 'PD-1-PD-L1 Blockade':ab,kw,ti OR 'Blockade, PD-1-PD-L1':ab,kw,ti OR 'PD 1 PD L1 Blockade':ab,kw,ti OR 'PD-1':ab,kw,ti OR 'PD-L1':ab,kw,ti OR 'pembrolizumab':ab,kw,ti OR 'atezolizumab':ab,kw,ti OR 'lisavanbulin':ab,kw,ti OR 'toripalimab ':ab,kw,ti OR 'sotorasib':ab,kw,ti OR 'spartalizumab':ab,kw,ti OR 'relatlimab':ab,kw,ti OR 'PHI-101':ab,kw,ti OR 'quemliclustat':ab,kw,ti OR 'Opdualag':ab,kw,ti<br>**#12 #10 OR #11**<br>**#13 #3 AND #6 AND #9 AND #12**
Cochrane Library	**#1** MeSH descriptor: [Triple Negative Breast Neoplasms] explode all trees<br>**#2** (ER Negative PR Negative HER2 Negative Breast Cancer):ti,ab,kw OR (ER Negative PR Negative HER2 Negative Breast Cancer):ti,ab,kw OR (ER Negative PR Negative HER2 Negative Breast Neoplasms):ti,ab,kw OR (ER Negative PR Negative HER2 Negative Breast Neoplasms):ti,ab,kw OR (Triple Negative Breast Cancer):ti,ab,kw OR (Triple Negative Breast Cancer):ti,ab,kw OR (Breast Cancers, Triple Negative):ti,ab,kw OR (Breast Cancer, Triple Negative):ti,ab,kw OR (Triple Negative Breast Cancers):ti,ab,kw OR (Triple Negative Breast Neoplasm):ti,ab,kw OR (Breast Neoplasms, Triple Negative):ti,ab,kw OR (Breast Neoplasm, Triple Negative):ti,ab,kw OR (Triple Negative Breast Neoplasm):ti,ab,kw OR (Triple Negative Breast Neoplasms):ti,ab,kw<br>**#3 #1 OR #2**<br>**#4** MeSH descriptor: [Drug Therapy] explode all trees<br>**#5** (Chemotherapy):ti,ab,kw OR (Chemotherapies):ti,ab,kw OR (Consolidation Chemotherapy):ti,ab,kw OR (Chemotherapies, Consolidation):ti,ab,kw OR (Chemotherapy, Consolidation):ti,ab,kw OR (Consolidation Chemotherapies):ti,ab,kw OR (Maintenance Chemotherapy'

	OR 'Chemotherapies, Maintenance):ti,ab,kw OR (Chemotherapy, Maintenance):ti,ab,kw OR (Maintenance Chemotherapies):ti,ab,kw OR (Antineoplastic Combined Chemotherapy Protocols):ti,ab,kw OR (Combined Antineoplastic Agents):ti,ab,kw OR (Antineoplastic Agents, Combined):ti,ab,kw OR (Agent, Combined Antineoplastic):ti,ab,kw OR (Antineoplastic Agent, Combined):ti,ab,kw OR (Combined Antineoplastic Agent):ti,ab,kw OR (Anticancer Drug Combinations):ti,ab,kw OR (Anticancer Drug Combination):ti,ab,kw OR (Drug Combination, Anticancer):ti,ab,kw OR (Drug Combinations, Anticancer):ti,ab,kw OR (Drug Combinations, Antineoplastic):ti,ab,kw OR (Antineoplastic Combined Chemotherapy Regimens):ti,ab,kw OR (Antineoplastic Drug Combinations):ti,ab,kw OR (Antineoplastic Drug Combination):ti,ab,kw OR (Combinations, Antineoplastic Drug):ti,ab,kw OR (Drug Combination, Antineoplastic):ti,ab,kw OR (Antineoplastic Chemotherapy Protocols):ti,ab,kw OR (Antineoplastic Chemotherapy Protocol):ti,ab,kw OR (Chemotherapy Protocol, Antineoplastic):ti,ab,kw OR (Protocol, Antineoplastic Chemotherapy):ti,ab,kw OR (Protocols, Antineoplastic Chemotherapy):ti,ab,kw OR (Cancer Chemotherapy Protocols):ti,ab,kw OR (Cancer Chemotherapy Protocol):ti,ab,kw OR (Chemotherapy Protocol, Cancer):ti,ab,kw OR (Chemotherapy Protocols, Cancer):ti,ab,kw OR (Protocol, Cancer Chemotherapy):ti,ab,kw OR (Protocols, Cancer Chemotherapy):ti,ab,kw OR (Chemotherapy Protocols, Antineoplastic):ti,ab,kw OR (albumin-bound paclitaxel):ti,ab,kw OR (paclitaxel):ti,ab,kw OR (gemcitabine):ti,ab,kw OR (carboplatin):ti,ab,kw OR (Capecitabine):ti,ab,kw OR (Vinorelbine):ti,ab,kw OR (Etoposide):ti,ab,kw OR (Doxorubicin Liposome Injection):ti,ab,kw OR (Carboplatin):ti,ab,kw<br>**#6 #4 OR #5**<br>**#7** MeSH descriptor: [Randomized Controlled Trials as Topic] explode all trees<br>**#8** (randomized controlled trial):ti,ab,kw OR (controlled clinical trial):ti,ab,kw OR (Clinical Trials, Randomized):ti,ab,kw OR (Trials, Randomized Clinical):ti,ab,kw OR (Controlled Clinical Trials, Randomized):ti,ab,kw OR (Clinical Trials):ti,ab,kw<br>**#9 #7 OR #8**<br>**#10** MeSH descriptor: [Immune Checkpoint Inhibitors] explode all trees<br>**#11** (Abatacept):ti,ab,kw OR (Ipilimumab):ti,ab,kw OR (Nivolumab):ti,ab,kw OR (Immune Checkpoint Inhibitors):ti,ab,kw OR (Checkpoint Inhibitors, Immune):ti,ab,kw OR (Immune Checkpoint Blockers):ti,ab,kw OR (Checkpoint Blockers, Immune):ti,ab,kw OR (Immune Checkpoint Inhibitor):ti,ab,kw OR (Checkpoint Inhibitor, Immune):ti,ab,kw OR (CTLA 4 Inhibitors):ti,ab,kw OR (CTLA 4 Inhibitors):ti,ab,kw OR (Cytotoxic T Lymphocyte Associated Protein 4 Inhibitors):ti,ab,kw OR (Cytotoxic T Lymphocyte Associated Protein 4 Inhibitors):ti,ab,kw OR (Cytotoxic T Lymphocyte Associated Protein 4 Inhibitor):ti,ab,kw OR (Cytotoxic T Lymphocyte Associated Protein 4 Inhibitor):ti,ab,kw OR (CTLA 4 Inhibitor):ti,ab,kw OR (CTLA 4 Inhibitor):ti,ab,kw OR (PD 1 Inhibitors):ti,ab,kw OR (PD 1 Inhibitors):ti,ab,kw OR (Programmed Cell Death Protein 1 Inhibitor):ti,ab,kw OR (Programmed Cell Death Protein 1 Inhibitors):ti,ab,kw OR (PD 1 Inhibitor):ti,ab,kw OR (Inhibitor, PD 1):ti,ab,kw OR (PD 1 Inhibitor):ti,ab,kw OR (Immune Checkpoint Blockade):ti,ab,kw OR (Checkpoint Blockade, Immune):ti,ab,kw OR (Immune Checkpoint Inhibition):ti,ab,kw OR (Checkpoint Inhibition, Immune):ti,ab,kw OR (PD L1 Inhibitors):ti,ab,kw OR (PD L1 Inhibitors):ti,ab,kw OR (Programmed Death Ligand 1 Inhibitors):ti,ab,kw OR (Programmed Death Ligand 1 Inhibitors):ti,ab,kw OR (PD L1 Inhibitor):ti,ab,kw OR (PD L1 Inhibitor):ti,ab,kw OR (PD 1 PD L1 Blockade):ti,ab,kw OR (Blockade, PD 1 PD L1):ti,ab,kw OR (PD 1 PD L1 Blockade):ti,ab,kw OR

	(pembrolizumab):ti,ab,kw OR (atezolizumab):ti,ab,kw OR (lisavanbulin):ti,ab,kw OR (toripalimab):ti,ab,kw OR (sotorasib):ti,ab,kw OR (spartalizumab):ti,ab,kw OR (relatlimab):ti,ab,kw OR (PHI 101):ti,ab,kw OR (quemliclustat):ti,ab,kw OR (Opdualag):ti,ab,kw OR (PD-1):ti,ab,kw OR (PD-L1):ti,ab,kw<br>**#12 #10 OR #11**<br>**#13 #3 AND #6 AND #9 AND #12**

Supplementary Table S2. Main Characteristics of the 7 studies included in meta-analysis

Clinical trial	Year	Sample size	Study design	Key inclusion criteria	Stratification factors	Experimental group	Control group	Primary end point
**IMpassion130**	2018	ITT: 902<br>Chemo-IO: 451; Chemo: 451<br>PD-L1+(IC ≥ 1%):369<br>Chemo -IO: 185; CT: 184	Phase III international, randomized, double-blind, placebo-controlled trial (randomisation 1:1)	Stage IV TNBC/mTNBC; No previous chemotherapy or targeted therapy for mTNBC; ECOG 0-1	Presence or absence of liver metastases; Use or nonuse of neoadjuvant or adjuvant taxane treatment; PD-L1 expression on tumor-infiltrating immune cells as a percentage of tumor area	Atezolizumab + nab-paclitaxel	Placebo + nab-paclitaxel	PFS, OS, ORR, DOR, AEs
	2020							PFS, OS, AEs
	2021							PFS, OS, , AEs
**KEYNOTE-355**	2020	ITT: 847<br>Chemo-IO: 566; Chemo: 281<br>PD-L1 CPS≥10: 323	Phase III international, randomized, double-blind, placebo-controlled trial	Locally recurrent inoperable or metastatic TNBC; ECOG 0-1	Type of on-study chemotherapy received (taxane or gemcitabine-carboplatin); tumour PD-L1	Pembrolizumab + chemotherapy	Placebo + chemotherapy	PFS, OS, ORR, DOR, DCR, AEs

	2022	Chemo-IO: 220; Chemo: 103<br>PD-L1 CPS⩾1: 636<br>Chemo-IO: 425; Chemo: 211	(randomisation 2:1)		expression at baseline (CPS⩾1 or CPS<1); Previous treatment with the same class of chemotherapy in the neoadjuvant or adjuvant setting (yes or no)			PFS, OS, ORR, AEs
**IMpassion131**	2021	ITT: 651<br>Chemo-IO: 431; Chemo: 220<br>PD-L1+(IC ⩾ 1%):292<br>Chemo-IO: 191; Chemo: 101	Phase III international, randomized, double-blind, placebo-controlled trial (randomisation 2:1)	Metastatic or unresectable locally advanced measurable TNBC; Eligible for taxane； ECOG 0-1	PD-L1 status; Prior taxane therapy; Liver metastases; Geographical region	Atezolizumab + paclitaxel	Placebo + paclitaxel	PFS, OS, ORR, EORTC QLQ-C30, GHS/HRQoL, AEs
**ALICE**	2022	FAS: 68<br>Chemo-IO: 40; Chemo: 28	Phase IIb multi-center, randomized, double-blind, parallel-group,	Metastatic or incurable locally advanced, histologically documented	PD-L1 status	Atezolizumab + anthracycline-based chemotherapy	Placebo + anthracycline-based chemotherapy	PFS, OS, ORR, DOR, DRR, CBR, PROs, AEs

		PD-L1+(IC ≥ 1%):59<br><br>Chemo-IO: 36; Chemo: 23	placebo-controlled trail (randomisation 3:2)	TNBC; Adjuvant treatment with anthracyclines or cyclophosphamide; No more than one prior line of chemotherapy in the metastatic setting; ECOG 0-1; DFS≥12 months				
**TBCRC 043**	2024	ALL: 106<br><br>Chemo-IO: 56; Chemo: 50<br><br>PD-L1+(IC>1%):20<br><br>Chemo-IO: 10; Chemo: 10	Phase II prospective, multi-center, randomized, double-blind trail (randomisation 1:1)	Clinical stage IV TNBC/ metastatic invasive TNBC; 0 to 1 prior treatments for metastatic disease; No prior carboplatin in the metastatic setting; ECOG 0-1	PD-L1 status; Prior therapy; Metastatic status	Atezolizumab + Carboplatin	Carboplatin	PFS, OS, ORR, DOR; CBR, AEs

**TORCHLIGHT**	2024	ITT: 531<br><br>Chemo-IO: 353; Chemo: 178<br><br>PD-L1+(CPS ⩾ 1):300<br><br>Chemo-IO: 200; Chemo: 100	Phase III multi-center, randomized, double-blind, parallel-group, placebo-controlled trail (randomisation 2:1)	Stage IV TNBC/ Locally advanced TNBC not amenable to surgery; No more than one previous systemic chemotherapy regimen; ECOG 0-1	PD-L1 expression status; Previous history of paclitaxel therapy; Current line of therapy	Toripalimab + nab-paclitaxel	Placebo + nab-paclitaxel	PFS, OS, ORR, DCR, DOR, CBR, AEs
**IMpassion132**	2024	mITT: 380<br><br>Chemo-IO: 188; Chemo: 192<br><br>PD-L1+(CPS ⩾ 1):354<br><br>Chemo-IO: 177; Chemo: 177	Phase III international, randomized, double-blind, placebo-controlled trial (randomisation 1:1)	Early relapsing unresectable locally advanced /aTNBC; No prior chemotherapy or systemic targeted therapy for aTNBC; Anthracycline and taxane-containing neoadjuvant or adjuvant	Investigator-selected chemotherapy; metastases status; PD-L1 status	Pembrolizumab + chemotherapy	Placebo + chemotherapy	OS, 12-month&18-month survival rates, PFS, ORR, DOR, CBR, AEs

				chemotherapy for eTNBC				

ITT, Intent - to - Treat; Chemo-IO, Chemoimmunotherapy; Chemo, Chemotherapy; mTNBC, Metastatic Triple Negative Breast Cancer; aTNBC, Advanced Triple Negative Breast Cancer; eTNBC, Early Triple Negative Breast Cancer; ECOG, Eastern Cooperative Oncology Group Performance Status; PFS, Progression Free Survival; OS, Overall Survival; ORR, Objective Response Rate; DOR, Duration of Response; AE, Adverse Event; DCR, Disease Control Rate; IC, Immune Cell; CPS, Combined Positive Score; DRR, durable response rate; CBR, Clinical Benefit Rate; PROs, Patient - Reported Outcomes; mITT, Modified Intent - to - Treat; EORTC QLQ - C30, European Organisation for Research and Treatment of Cancer Quality of Life Questionnaire - Core 30; GHS/HRQoL, Global Health Status / Health - Related Quality of Life;

Supplementary Table S3: Race distribution of 7 studies included in meta-analysis

Race	Impassion130	KEYNOTE-355	Impassion131	ALICE	TBCRC 043	TORCHLIGHT	Impassion132	Total
**White**	609	579	374	NR	73	NR	281	1916
**Asian**	161	175	189	NR	1	NR	49	575
**Black/African American**	59	37	31	NR	12	NR	11	150
**Native American/American Indian/Alaska Native**	40	12	NR	NR	NR	NR	6	58
**Hawaiian or other Pacific Islander**	1	NR	NR	NR	NR	NR	NR	1
**Multiple/Other**	5	19	6	NR	NR	NR	1	31
**Unknown/Missing**	27	25	37	NR	11	NR	16	116

NR, not reported

Supplementary Table S4: Baseline Metastatic status of 7 studies included in meta-analysis

Metastatic site	IMpassion130	KEYNOTE-355	IMpassion131	ALICE	TBCRC 043	TORCHLIGHT	IMpassion132	Total
**Bone**	286	254	200	33	36	179	NR	988
**Brain**	61	26	NR	NR	6	6	NR	99
**Liver**	244	249	179	25	4	119	103	923
**Lymph node**	56	623	NR	35	54	NR	NR	768
**Lung**	468	486	330	28	50	283	206	1851

NR, not reported

Supplementary Table S5: Baseline previous therapy of 7 studies included in meta-analysis

	IMpassion130	KEYNOTE-355	IMpassion131	ALICE	TBCRC 043	TORCHLIGHT	IMpassion132	Total
**Neoadjuvant or adjuvant therapy**	570	538	NR	NR	75	401	NR	1584
**Taxane**	461	446	315	NR	NR	NR	377	1599
**Anthracycline**	485	473	322	42	NR	NR	377	1699
**Platinum**	NR	65	NR	NR	15	NR	82	162
**Paclitaxel**	NR	NR	NR	NR	NR	97	NR	97
**Capecitabine**	NR	NR	NR	NR	NR	NR	77	77
**Other**	NR	498	NR	NR	NR	NR	NR	498

NR, not reported

Supplementary Table S6. Treatment exposure of 7 studies included in meta-analysis

Study	Treatments	Experimental group		Control group	
		ICI, Median (range) treatment duration, months	CT, Median (range) treatment duration, months	Placebo, Median (range) treatment duration, months	CT, Median (range) treatment duration, months
**IMpassion130**	Patients received atezolizumab at a dose of 840 mg or placebo, administered intravenously, on days 1 and 15 and received nab-paclitaxel at a dose of 100 mg per square meter of body-surface area, administered intravenously, on days 1, 8, and 15 of every 28-day cycle.	Atezolizumab: 24.1(0-139)	Nab-paclitaxel: 22.1(0-137)	Placebo: 22.1(0-109)	Nab-paclitaxel: 21.8(0-103)
**KEYNOTE-355**	In part 1, patients received 200 mg of pembrolizumab (Keytruda, Merck Sharp and Dohme) every 3 weeks in combination with one of three chemotherapy options (nab-paclitaxel 100 mg/m2 on days 1, 8, and 15, every 28 days; paclitaxel 90 mg/m2 on days 1, 8, and 15, every 28 days; or gemcitabine 1000 mg/m2 plus carboplatin area under the curve 2 on days 1 and 8, every 21 days). In part 2, patients received pembrolizumab – chemotherapy (investigator's choice of nab-paclitaxel; paclitaxel; or gemcitabine-carboplatin, as described) or placebo – chemotherapy for up to 35 administrations (pembrolizumab or placebo only; chemotherapy was continued at the investigator's discretion) or until confirmed disease progression, unacceptable toxicity, withdrawal of consent, or physician's decision. Crossover between treatment groups was not permitted.	Pembrolizumab:24.2(0.1-140.1)	Nab-paclitaxel(n=174): 23.1(0.1-212.1)<br><br>Paclitaxel(n=81): 21.6(1.1-108.9)<br><br>Gemcitabine(n=309): 22.1(0.1-199.1)<br><br>Carboplatin(n=309): 22.1(0.1-199.1)	Placebo: 22.1(0.1-119.6)	Nab-paclitaxel(n=95): 18.1(0.1-208.1)<br><br>Paclitaxel(n=32): 17.4(0.1-208.1)<br><br>Gemcitabine(n=154): 23.1(0.1-215.7)<br><br>Carboplatin(n=154): 22.9(0.1-215.7)

**IMpassion131**	Patients received intravenous paclitaxel 90 mg/m2 on days 1, 8 and 15 every 28 days in combination with either intravenous atezolizumab 840 mg or placebo, administered on days 1 and 15 every 28 days.	Atezolizumab: 5.1(0-35)	Paclitaxel: 4.9(0-35)	Placebo: 5.2(0-28)	Paclitaxel: 5.1(0-22)
**ALICE**	PLD (20 mg/m2 i.v. on day 1 of each 14-day cycle) and cyclophosphamide (50 mg by mouth (p.o.) daily in every other 14-day cycle). Atezolizumab (840 mg) or placebo was given i.v. on day 1 of each cycle.	Atezolizumab+CT: 5.2(IQR 2.3 – 7.2)		Placebo+CT: 3.2(IQR 2.0 – 5.7)	
**TBCRC 043**	Patients received intravenous carboplatin area under the curve (AUC) 6 alone or in combination with atezolizumab, 1200 mg, every 3 weeks.	Atezolizumab+Carboplatin: 17.4(1.4-90.3)		Carboplatin: 15.4(3.0-72.1)	
**TORCHLIGHT**	Patients received either toripalimab 240 mg or placebo on day 1 of each 21-d cycle and nab-P 125 mg m−2 on day 1 and day 8 of each cycle.	Toripalimab: 21.143(0.143-129.429)	Nab-paclitaxel: 22.143(0.143-117.0)	Placebo: 22.143(0.143-125.571)	Nab-paclitaxel: 22.429(0.143-125.571)
**IMpassion132**	Patients received either placebo or atezolizumab 1200 mg on day 1 every 21 days with the chosen chemotherapy. Before randomisation, investigators selected one of two chemotherapy options: intravenous gemcitabine 1000 mg/m2 plus carboplatin area under the curve 2 mg/ml/min both on days 1 and 8 every 21 days, or oral capecitabine 1000 mg/m2 twice daily on days 1-14 every 21 days. Capecitabine was mandatory if patients had received platinum-containing therapy for eTNBC; the proportion of patients receiving capecitabine was capped at ~30% of the overall population.	Atezolizumab:2.8(0-62)	Carboplatin(n=208): 3.4 (0-43)<br><br>Gemcitabine(n = 209): 3.4 (0-56)<br><br>Capecitabine(n = 83): 2.5 (0 – 28)	Placebo: 2.9(0-61)	Carboplatin(n=210): 3.2 (0-49)<br><br>Gemcitabine(n = 210): 3.2 (0-61)<br><br>Capecitabine(n = 84): 2.6 (0-61)

ICI, Immune Checkpoint Inhibitor; Chemo, Chemotherapy; i.v. Intravenous Injection; p.o. per os

Supplementary Table S7. Subgroup analyses on progression-free survival based on age, race, and other baseline characteristics. Data are presented as hazard ratios (HR) with 95% confidence intervals (CI) for each subgroup.

Subgroups	HR	95%CI
**PD-L1**		
Mean PD-L1 expression ＜1	0.93	0.82-1.06
Mean PD-L1 expression ⩾1	0.68	0.59- 0.79
**Age**		
Mean age less than 64 year-old	0.79	0.71–0.87
Mean age more than 64 year-old	0.77	0.63–0.93
**Race**		
White	0.76	0.66–0.88
Aisa	0.83	0.65–1.07
Black or African American	0.83	0.51–1.37
**ECOG**		
0	0.77	0.69–0.87
1	0.83	0.73–0.95
**Metastatic site**		
Lung(+)	0.78	0.67–0.91
Liver(+)	0.80	0.66–0.98
Bone(+)	0.90	0.74-1.10
**Previous neoadjuvant or adjuvant chemotherapy**		
Previous adjuvant chemotherapy	0.86	0.76–0.98
Previous neoadjuvant chemotherapy	0.69	0.58–0.81

Supplementary Table S8. The result of PFS in the age ≤64 years subgroup of patients with chemoimmunotherapy vs. chemotherapy in mTNBC before the trim and fill procedure.

Method	Pooled Est	95% CI Lower	95% CI Upper	Asymptotic z_value	Asymptotic p_value	No. of studies
**Fixed**	-0.240	-0.338	-0.143	-4.841	0.000	6
**Random**	-0.240	-0.338	-0.143	-4.841	0.000	6

Supplementary Table S9. Trimming estimator of the result of PFS in the age ≤64 years subgroup of patients with chemoimmunotherapy vs. chemotherapy in mTNBC.

iteration	estimate	Tn	# to trim	diff
**1**	-0.240	5	0	21
**2**	-0.240	5	0	0

Supplementary Table S10. The result of PFS in the age ≤64 years subgroup of patients with chemoimmunotherapy vs. chemotherapy in mTNBC after the trim and fill procedure.

Method	Pooled Est	95% CI Lower	95% CI Upper	Asymptotic z_value	Asymptotic p_value	No. of studies
**Fixed**	0.786	0.713	0.867	-4.841	0.000	6
**Random**	0.786	0.713	0.867	-4.841	0.000	6

Supplementary Table S11. The result of PFS in the age＞64 years subgroup of patients with chemoimmunotherapy vs. chemotherapy in mTNBC before the trim and fill procedure.

Method	Pooled Est	95% CI Lower	95% CI Upper	Asymptotic z_value	Asymptotic p_value	No. of studies
**Fixed**	-0.267	-0.466	-0.068	-2.634	0.008	6
**Random**	-0.267	-0.466	-0.068	-2.634	0.008	6

Supplementary Table S12. Trimming estimator of the result of PFS in the age＞64 years subgroup of patients with chemoimmunotherapy vs. chemotherapy in mTNBC.

iteration	estimate	Tn	# to trim	diff
**1**	-0.267	16	2	21
**2**	-0.327	18	3	4
**3**	-0.340	19	3	2
**4**	-0.340	19	3	0

Supplementary Table S13. The result of PFS in the age＞64 years subgroup of patients with chemoimmunotherapy vs. chemotherapy in mTNBC after the trim and fill procedure.

Method	Pooled Est	95% CI Lower	95% CI Upper	Asymptotic z_value	Asymptotic p_value	No. of studies
**Fixed**	0.712	0.595	0.852	-3.701	0.000	9
**Random**	0.712	0.595	0.852	-3.701	0.000	9

Supplementary Table S14. The Outcomes of Begg's test and Egger's test for PFS in the ITT Population and Subgroups.

Outcomes	No. of studies	Begg's test		Egger's test	
		Z	*P*r>|z|	t	*P*>|t|
**ITT Population**	7	1.20	0.23	-1.57	0.178
**PD-L1**					
Mean PD-L1 expression ＜1	5	0.73	0.462	-0.98	0.400
Mean PD-L1 expression ⩾1	5	0.24	0.806	-0.07	0.947
**Age**					
Mean age less than 64 year-old	6	1.50	0.133	-3.47	0.026
Mean age more than 64 year-old	6	1.88	0.060	3.39	0.027
Race					
White	3	0.00	1.000	-0.31	0.806
Aisa	3	1.04	0.296	4.13	0.151
Black or African American	3	0.00	1.000	-0.02	0.988
**ECOG**					
0	6	1.50	0.133	-1.50	0.207
1	6	0.00	1.000	-0.95	0.395
**Metastatic site**					
Lung(+)	4	-0.34	1.000	-1.80	0.214
Liver(+)	5	0.73	0.462	-0.75	0.505
Bone(+)	4	1.02	0.308	-1.72	0.227
**Previous neoadjuvant or adjuvant chemotherapy**					
Previous adjuvant chemotherapy	3	0.00	1.000	-0.93	0.524
Previous neoadjuvant chemotherapy	3	0.00	1.000	0.12	0.922

Supplementary Table S15. Subgroup analyses on overall survival based on age, race, and other baseline characteristics. Data are presented as hazard ratios (HR) with 95% confidence intervals (CI) for each subgroup.

Subgroups	HR	95%CI
**PD-L1**		
Mean PD-L1 expression ＜1	1.01	0.87–1.17
Mean PD-L1 expression ⩾1	0.84	0.69–1.04
**Age**		
Mean age less than 65 year-old	0.83	0.73–0.95
Mean age more than 65 year-old	0.95	0.72–1.25
**ECOG**		
0	0.80	0.68–0.95
1	0.93	0.71–1.24
**Metastatic site**		
Lung(+) and/or Liver(+)	0.87	0.76–0.99

Supplementary Table S16. The Outcomes of Begg's test and Egger's test for OS in the ITT Population and Subgroups.

Outcomes	No. of studies	Begg's test		Egger's test	
		Z	*P*r>|z|	t	*P*>|t|
**ITT Population**	7	0.30	0.764	-1.12	0.313
**PD-L1**					
Mean PD-L1 expression ＜1	3	0.00	1.000	0.59	0.660
Mean PD-L1 expression ⩾1	4	1.70	0.089	-4.03	0.057
**Age**					
Mean age less than 65 year-old	3	0.00	1.000	-0.99	0.502
Mean age more than 65 year-old	3	0.00	1.000	0.42	0.745
**ECOG**					
0	3	1.04	0.296	-2.04	0.290
1	3	0.00	1.000	0.24	0.850
**Metastatic site**					
Lung(+) and/or Liver(+)	3	1.04	0.296	-0.81	0.568

Supplementary Table S17. Subgroup analyses on objective response rate . Data are presented as risk ratios (RR) with 95% confidence intervals (CI) for each subgroup.

Subgroups	RR	95%CI
**PD-L1**		
Mean PD-L1 expression ⩾1	1.10	0.97–1.25

Supplementary Table S18. Subgroup analyses on Clinical Benefit Rate. Data are presented as risk ratios (RR) with 95% confidence intervals (CI) for each subgroup.

Subgroups	RR	95%CI
**PD-L1**		
Mean PD-L1 expression ⩾1	1.15	1.01-1.31

Supplementary Table S19 The Outcomes of Begg's test and Egger's test for ORR in the ITT Population and Subgroups.

Outcomes	No. of studies	Begg's test		Egger's test	
		Z	*P*r>|z|	t	*P*>|t|
**ITT Population**	4	1.02	0.308	1.09	0.389
**PD-L1**					
Mean PD-L1 expression ≥1	4	1.02	0.308	1.02	0.414

Supplementary Table S20. The Outcomes of Begg's test and Egger's test for CBR in the ITT Population and Subgroups.

Outcomes	No. of studies	Begg's test		Egger's test	
		Z	*P*r>|z|	t	*P*>|t|
**ITT Population**	4	1.02	0.308	1.32	0.318
**PD-L1**					
Mean PD-L1 expression ≥1	3	1.04	0.296	4.91	0.128

Supplementary Table S21. Overall analysis on safety endpoints. Data are presented as risk ratios (RR) with 95% confidence intervals (CI).

Safety analysis	RR	95%CI
**AE**	1.01	0.99-1.02
**TEAE**	1.01	0.99-1.03
**Grade⩾3 AE**	1.11	1.03-1.20
**Grade⩾3 TEAE**	1.00	0.93-1.07
**SAE**	1.32	1.11-1.57
**irAE**	1.86	1.31-2.45
**Grade≥3 irAE**	3.57	2.26-5.65

Supplementary Table S22 The Outcomes of Begg's test and Egger's test for safety analysis in the ITT Population.

Outcomes	No. of studies	Begg's test		Egger's test	
		Z	*P*r>|z|	t	*P*>|t|
**ITT Population**	4	1.04	0.296	-1.57	0.316
**TEAE**	4	-0.34	1.000	0.12	0.915
**Grade≥3 AE**	3	1.04	0.296	2.77	0.221
**Grade⩾3 TEAE**	3	0.00	1.000	0.05	0.67
**SAE**	4	0.34	0.734	1.39	0.300
**irAE**	3	1.04	0.296	1.06	0.41
**Grade≥3 irAE**	5	0.73	0.462	1.66	0.196

Supplementary Figure S1 Bias Risk Assessment of Included Clinical Studies.()

A.Risk of bias graph

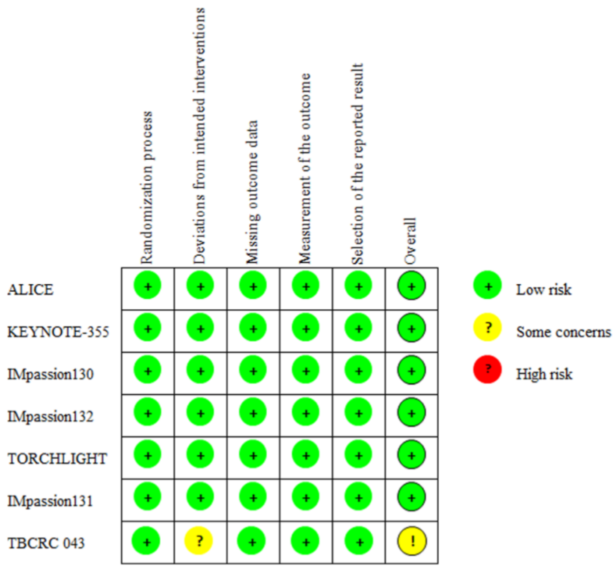

B.Risk of bias summary

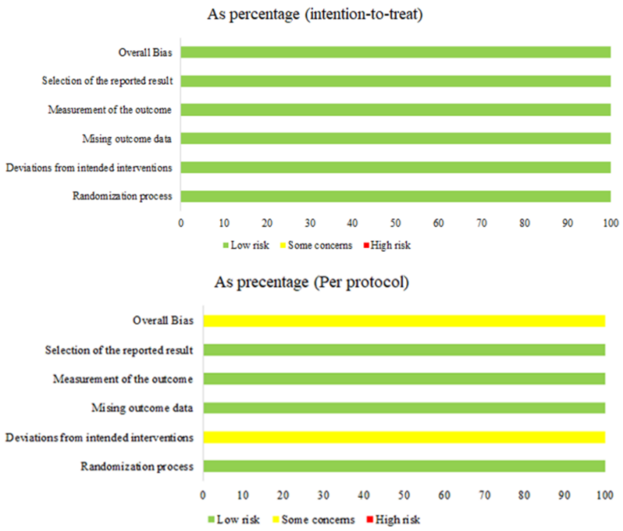

Supplementary Figure S2 Forest plots of PFS for chemoimmunotherapy vs. chemotherapy in mTNBC

A.PFS in the ITT population

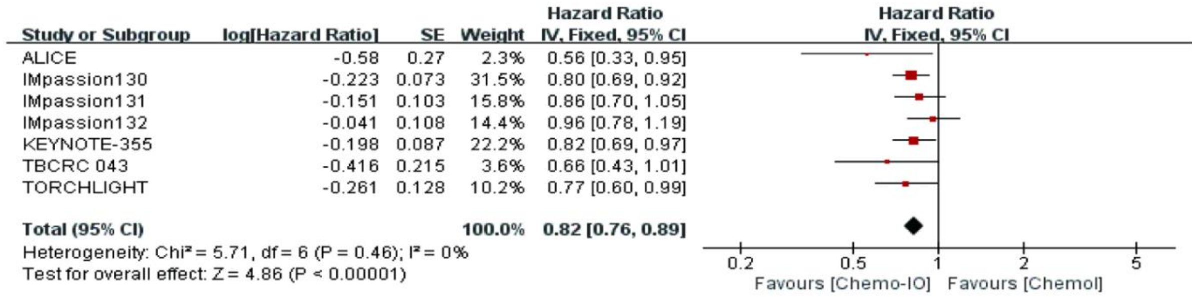

B.PFS in the PD-L1-positive population

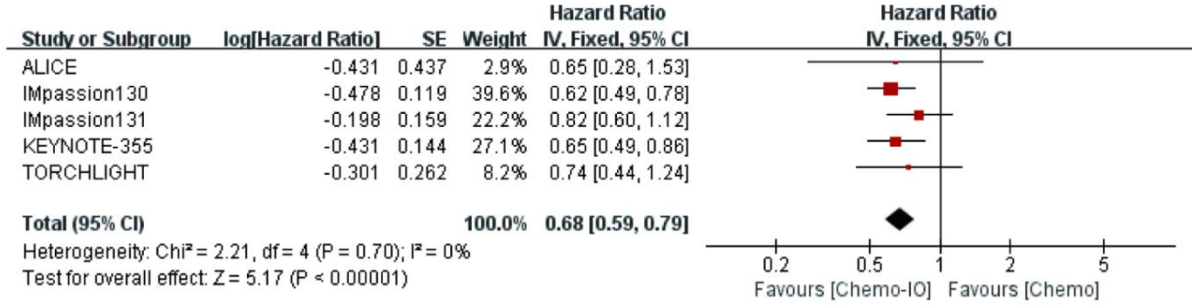

C.PFS in the PD-L1-negative population

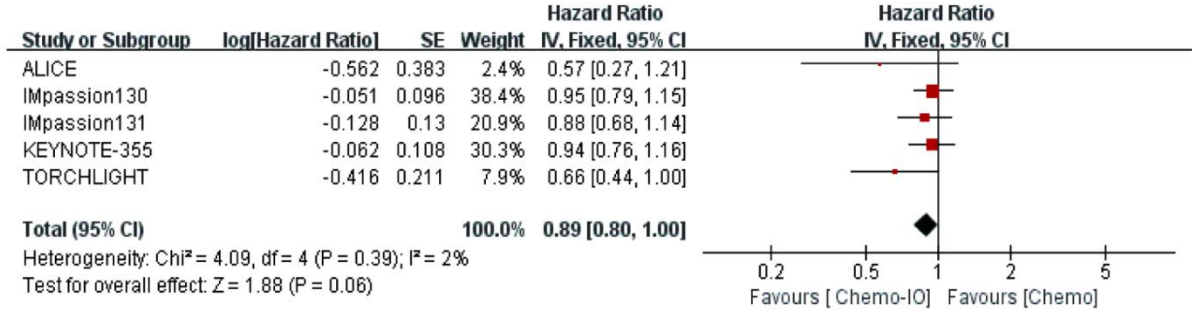

Supplementary Figure S3 Forest plots of PFS for chemoimmunotherapy vs. chemotherapy in mTNBC by subgroup.

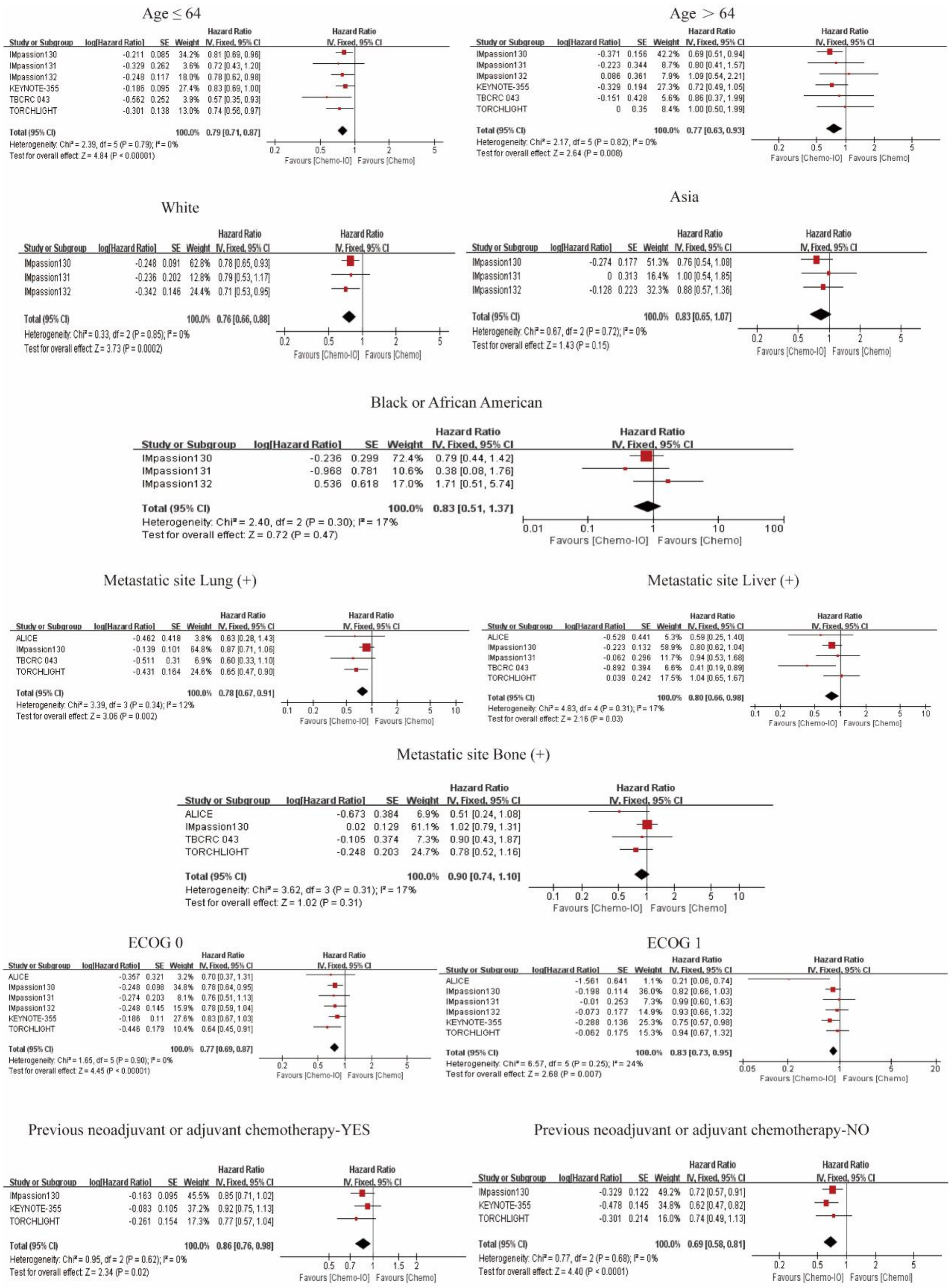

Supplementary Figure S4 The funnel plots of PFS for chemoimmunotherapy vs. chemotherapy in mTNBC by age subgroup after the trim and fill procedure.

Age ≤ 64

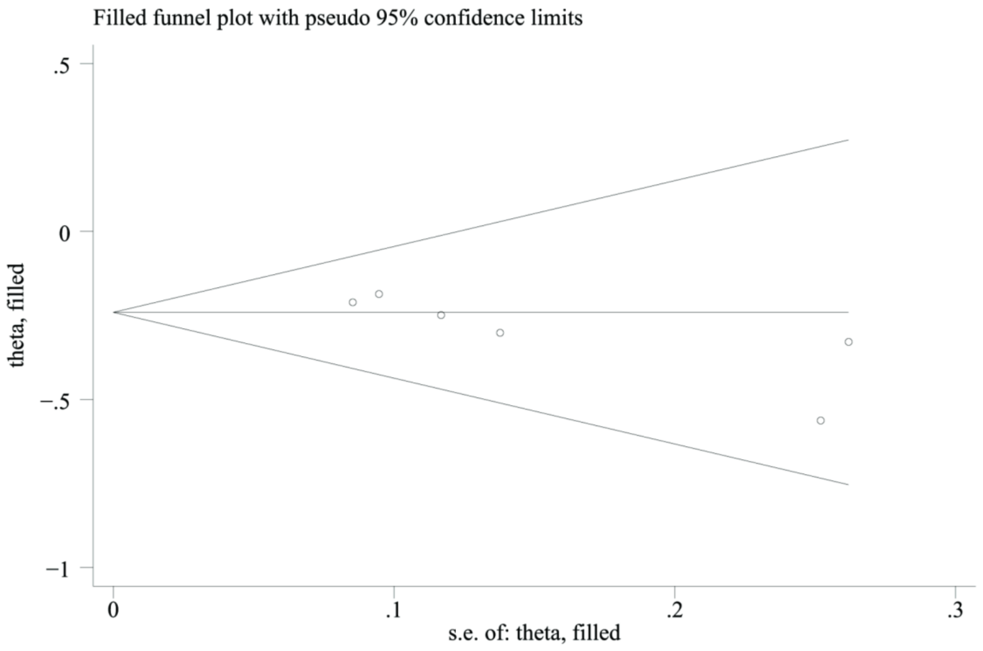

Age > 64

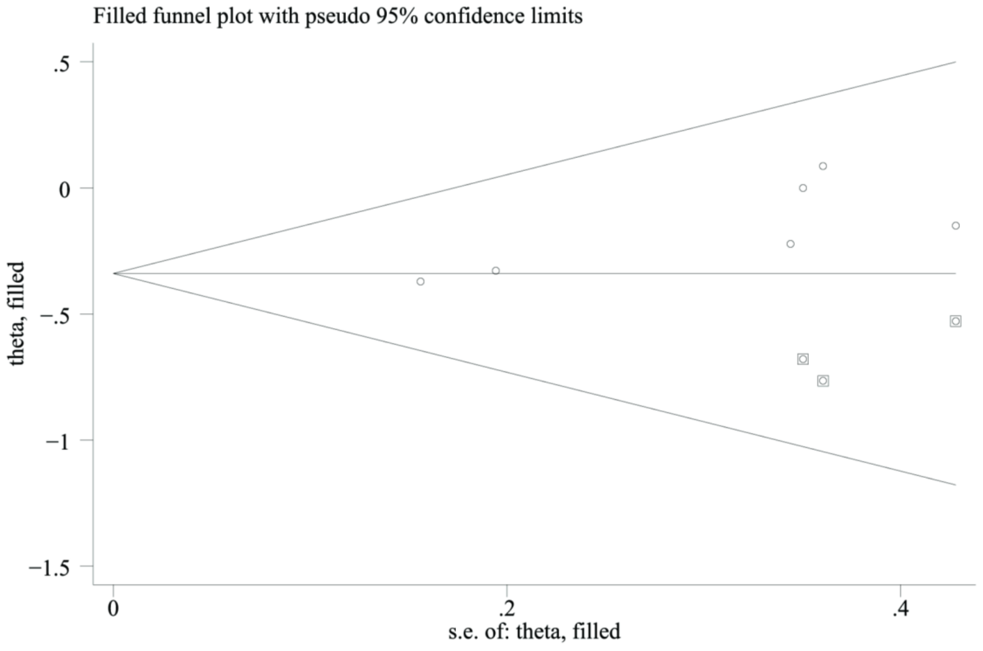

Supplementary Figure S5 The funnel plots of PFS for chemoimmunotherapy vs. chemotherapy in mTNBC (part 1)

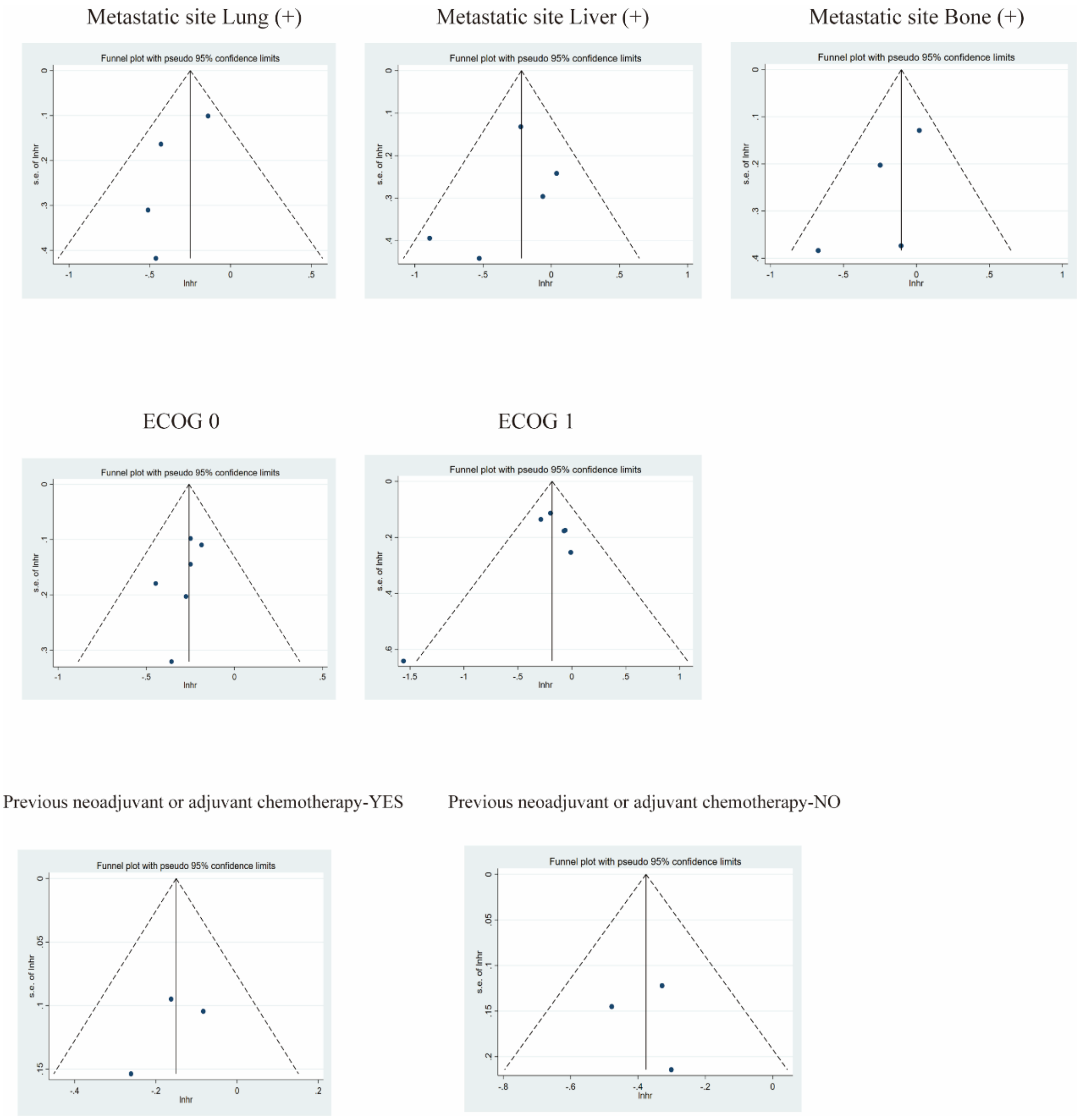

Supplementary Figure S6 The funnel plots of PFS for chemoimmunotherapy vs. chemotherapy in mTNBC(part 2)

Metastatic site Lung (+)

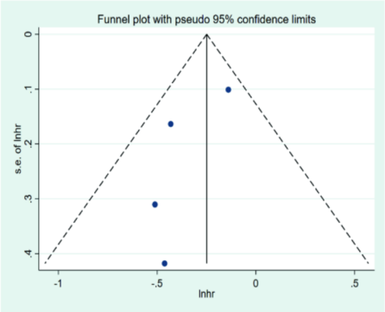

Metastatic site Liver (+)

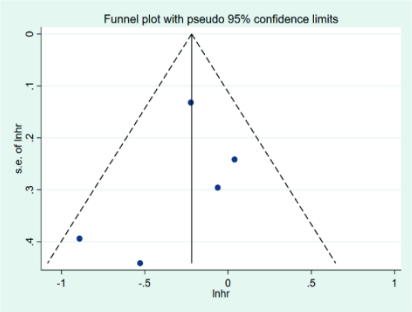

Metastatic site Bone (+)

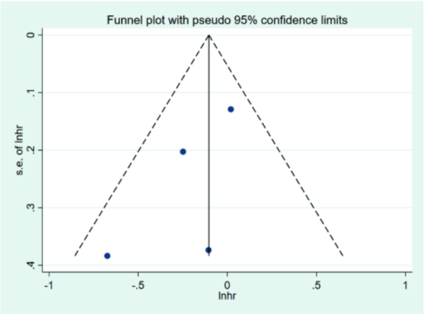

ECOG 0

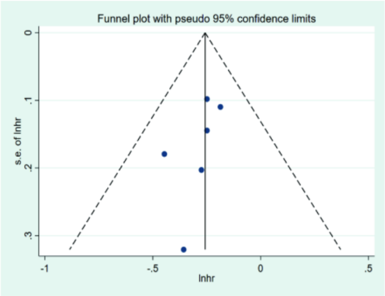

ECOG 1

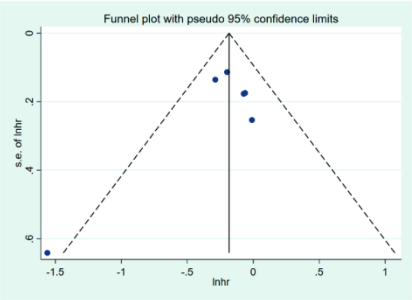

Previous adjuvant chemotherapy

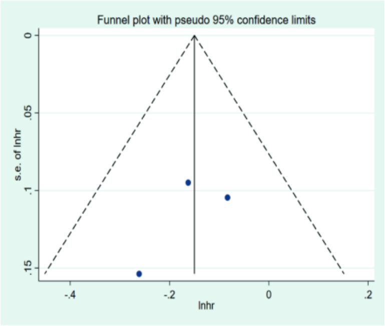

Previous neoadjuvant chemotherapy

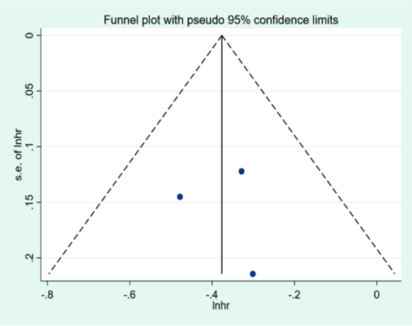

Supplementary Figure S7 Forest plots of OS for chemoimmunotherapy vs. chemotherapy in mTNBC.

A.OS in the ITT population

Study or Subgroup	log[Hazard Ratio]	SE	Weight	Hazard Ratio IV, Fixed, 95% CI
ALICE	-0.288	0.282	2.4%	0.75 [0.43, 1.30]
IMpassion130	-0.139	0.078	31.0%	0.87 [0.75, 1.01]
IMpassion131	0.113	0.124	12.3%	1.12 [0.88, 1.43]
IMpassion132	-0.062	0.112	15.0%	0.94 [0.75, 1.17]
KEYNOTE-355	-0.117	0.082	28.0%	0.89 [0.76, 1.04]
TBCRC 043	-0.511	0.243	3.2%	0.60 [0.37, 0.97]
TORCHLIGHT	-0.371	0.152	8.2%	0.69 [0.51, 0.93]
**Total (95% CI)**			**100.0%**	**0.88 [0.81, 0.96]**

Heterogeneity: Chi² = 9.52, df = 6 (P = 0.15); I² = 37%
Test for overall effect: Z = 2.87 (P = 0.004)

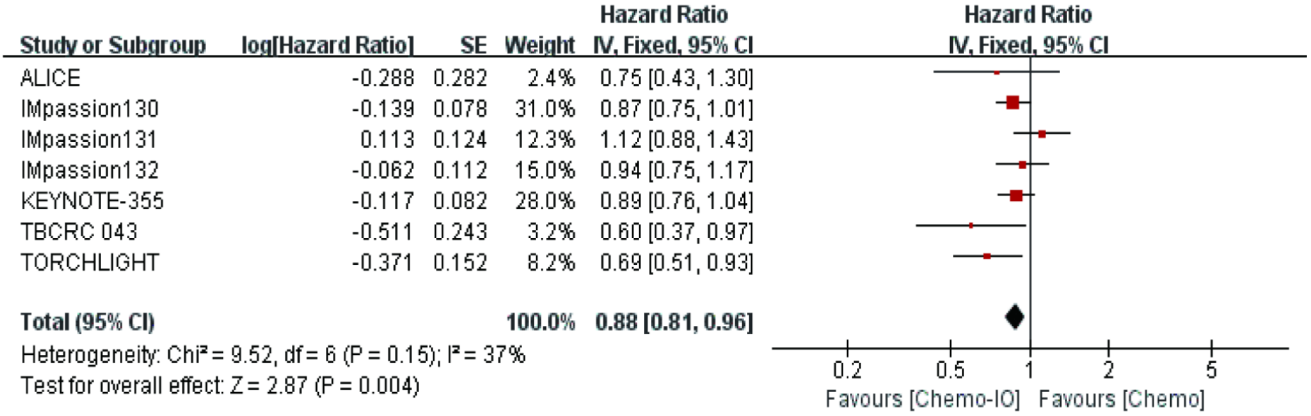

B.OS in the PD-L1-positive population

Study or Subgroup	log[Hazard Ratio]	SE	Weight	Hazard Ratio IV, Random, 95% CI
IMpassion130	-0.4	0.123	23.8%	0.67 [0.53, 0.85]
IMpassion131	0.104	0.206	16.8%	1.11 [0.74, 1.66]
IMpassion132	0.049	0.099	25.9%	1.05 [0.86, 1.28]
KEYNOTE-355	-0.315	0.139	22.4%	0.73 [0.56, 0.96]
TORCHLIGHT	-0.598	0.3	11.1%	0.55 [0.31, 0.99]
**Total (95% CI)**			**100.0%**	**0.82 [0.64, 1.04]**

Heterogeneity: Tau² = 0.05; Chi² = 13.28, df = 4 (P = 0.01); I² = 70%
Test for overall effect: Z = 1.62 (P = 0.11)

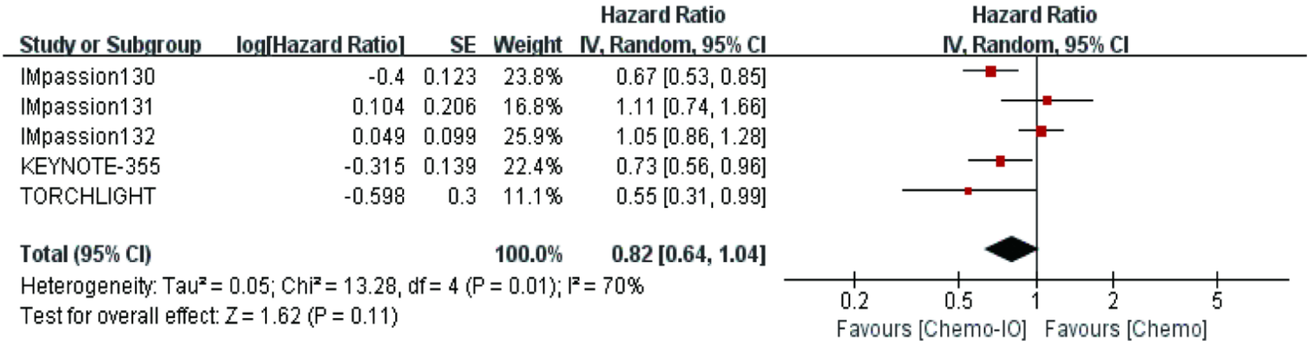

C.OS in the PD-L1-negative population

Study or Subgroup	log[Hazard Ratio]	SE	Weight	Hazard Ratio IV, Fixed, 95% CI
IMpassion130	0.068	0.118	31.4%	1.07 [0.85, 1.35]
IMpassion131	0	0.153	18.7%	1.00 [0.74, 1.35]
KEYNOTE-355	0.039	0.1	43.8%	1.04 [0.85, 1.26]
TORCHLIGHT	-0.4	0.267	6.1%	0.67 [0.40, 1.13]
**Total (95% CI)**			**100.0%**	**1.01 [0.89, 1.15]**

Heterogeneity: Chi² = 2.68, df = 3 (P = 0.44); I² = 0%
Test for overall effect: Z = 0.21 (P = 0.83)

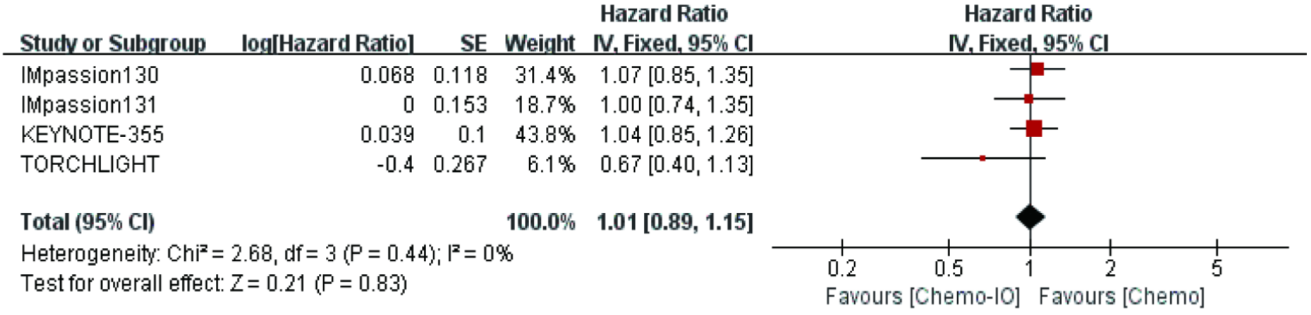

Supplementary Figure S8 Forest plots of OS for chemoimmunotherapy vs. chemotherapy in mTNBC by subgroup.

Age ＜ 65

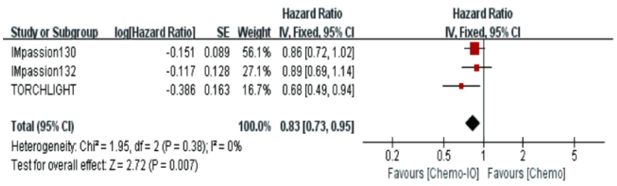

Age ≥ 65

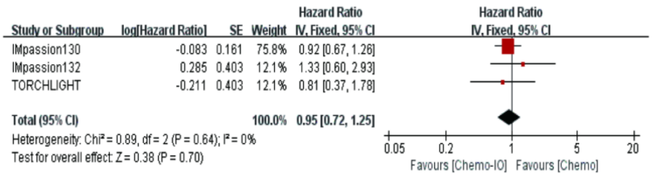

Metastatic site Lung and/or Liver（+）

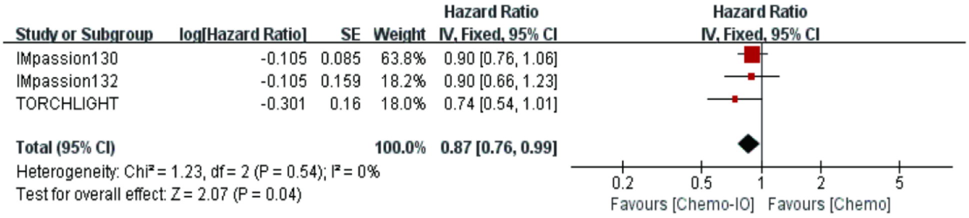

ECOG 0

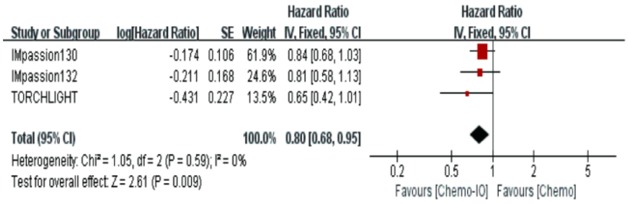

ECOG 1

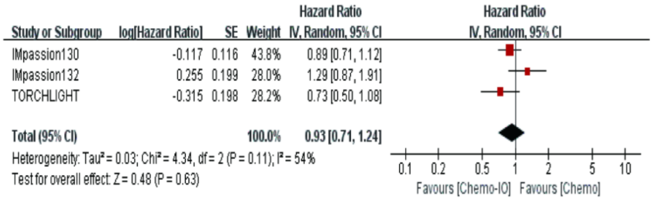

Supplementary Figure S9 The Galbraith radial plot of OS for chemoimmunotherapy vs. chemotherapy in mTNBC.

PD-L1-positive

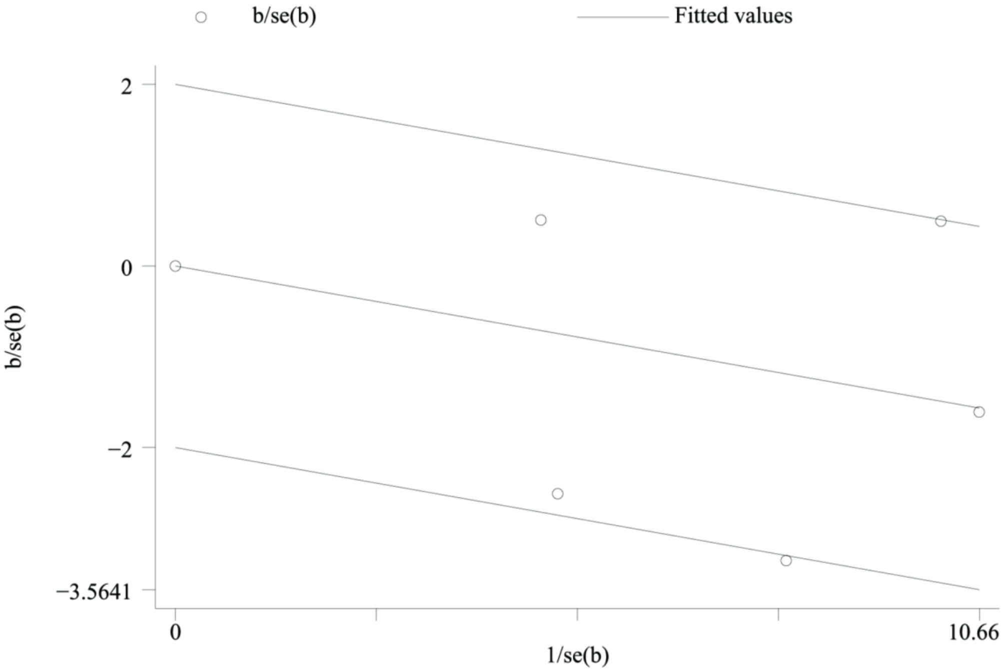

ECOG 1

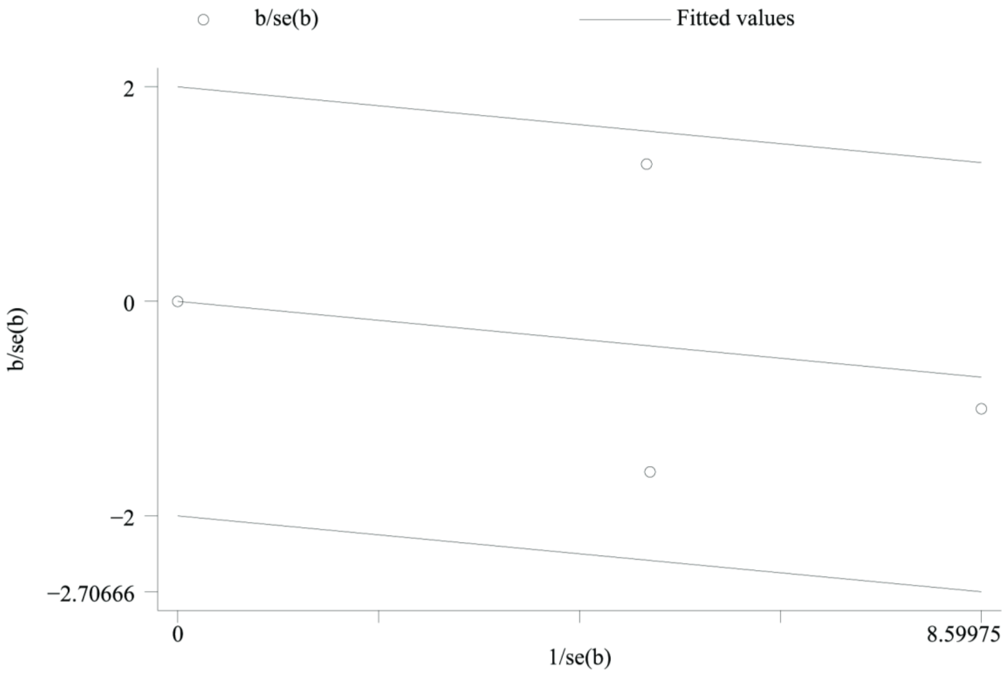

Supplementary Figure S10 The sensitivity analysis of OS for chemoimmunotherapy vs. chemotherapy in mTNBC.

PD-L1-positive

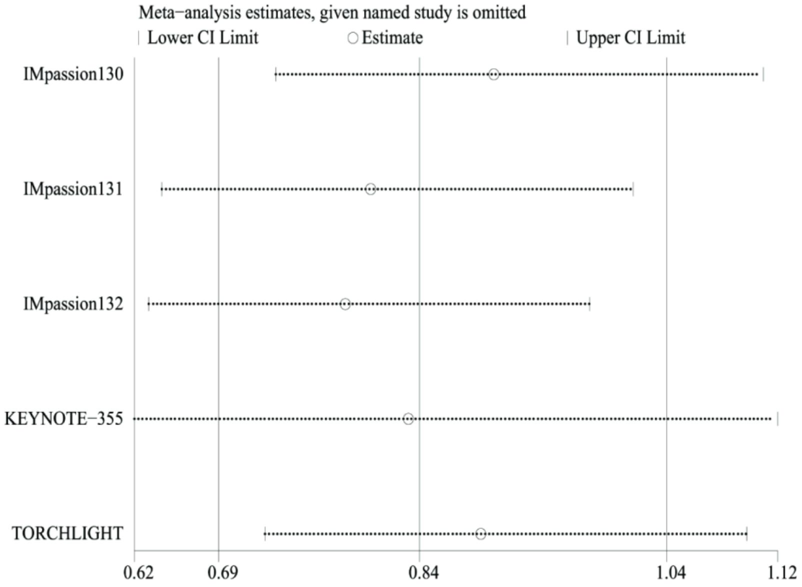

ECOG 1

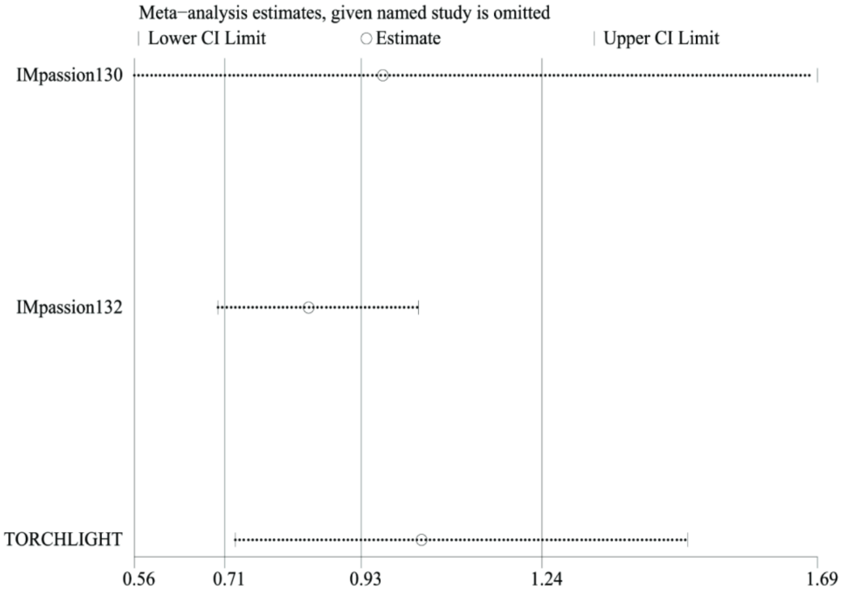

Supplementary Figure S11 The outcome of sensitivity analysis of OS for chemoimmunotherapy vs. chemotherapy in mTNBC.

PD-L1-positive

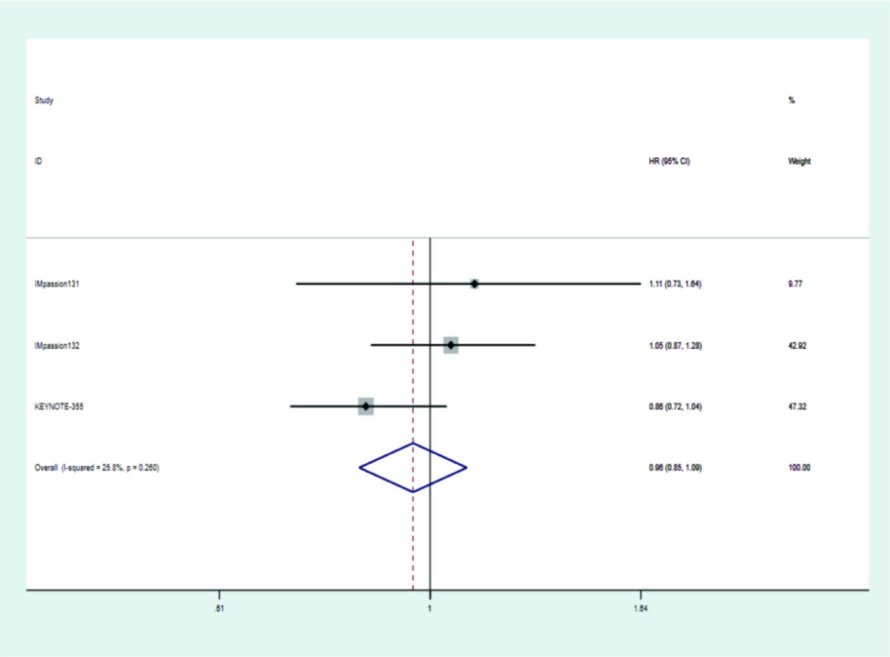

ECOG 1

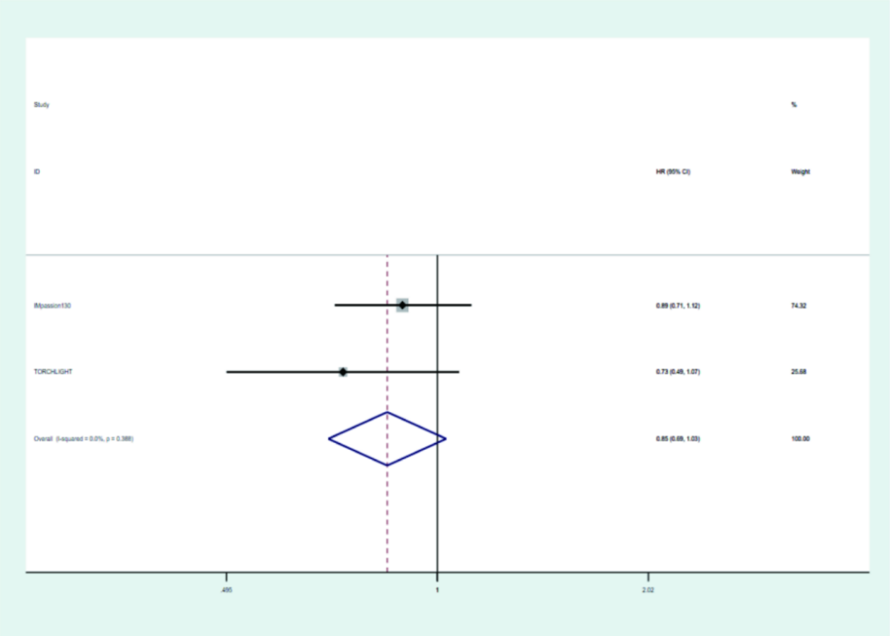

Supplementary Figure S12 The meta-regressions of OS for chemoimmunotherapy vs. chemotherapy in mTNBC according to publication year and sample size.

PD-L1-positive

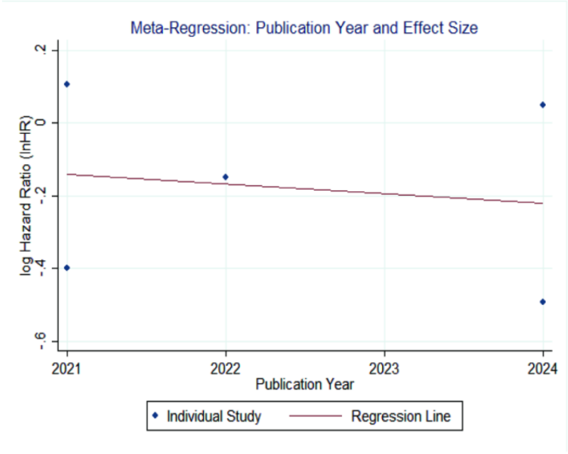

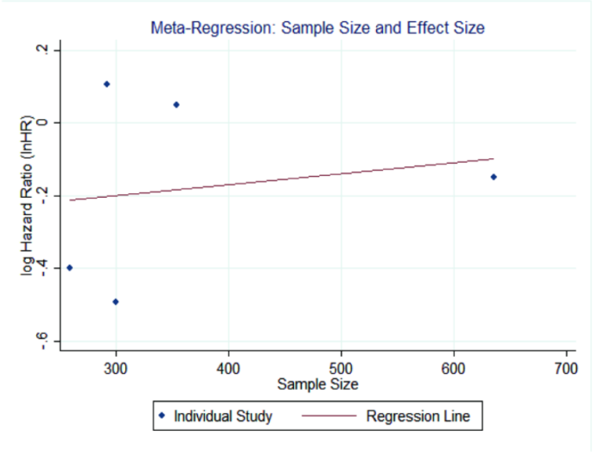

ECOG 1

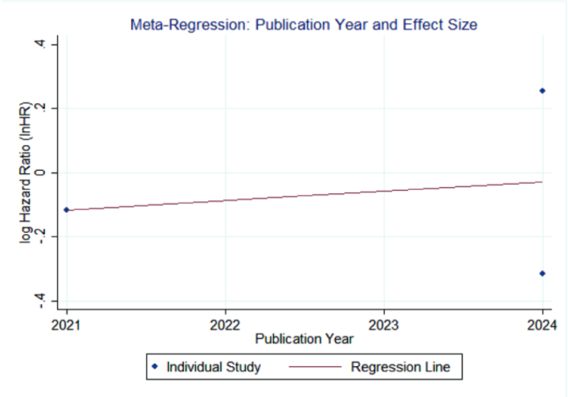

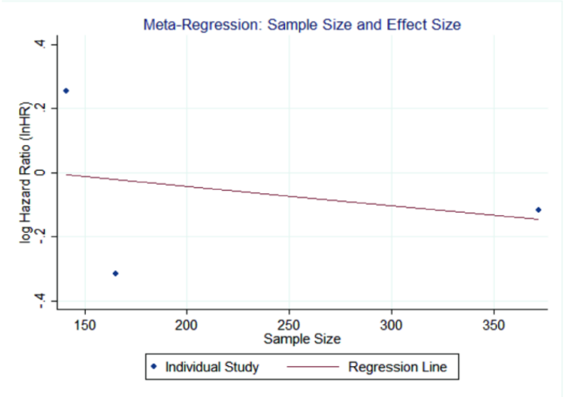

Supplementary Figure S13 The funnel plots of OS for chemoimmunotherapy vs. chemotherapy in mTNBC.

ITT

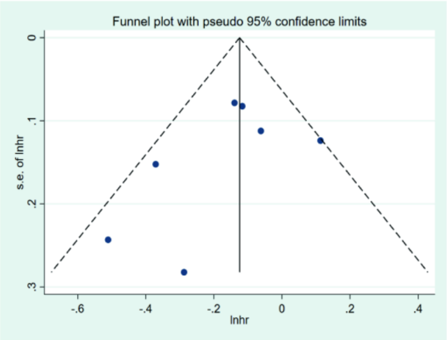

Metastatic site Lung and/or Liver (+)

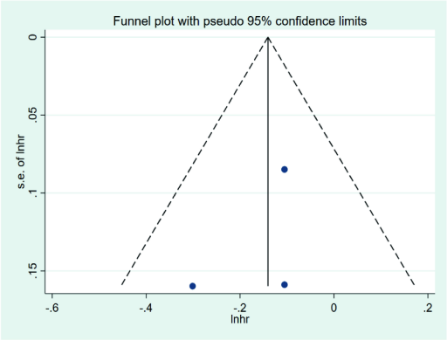

PD-L1-positive

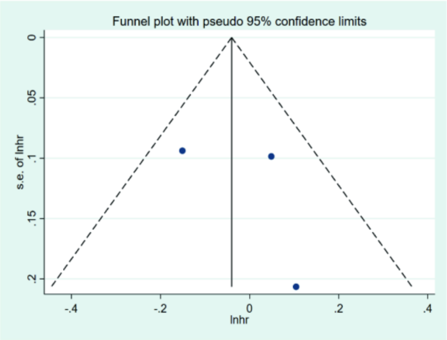

PD-L1-negative

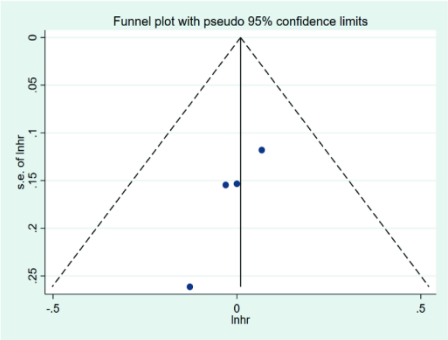

Age < 65

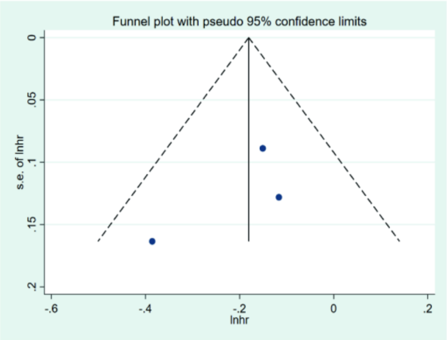

Age ≥ 65

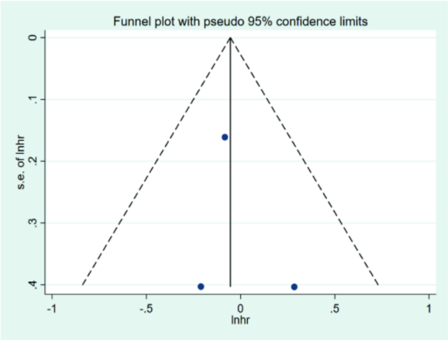

ECOG 0

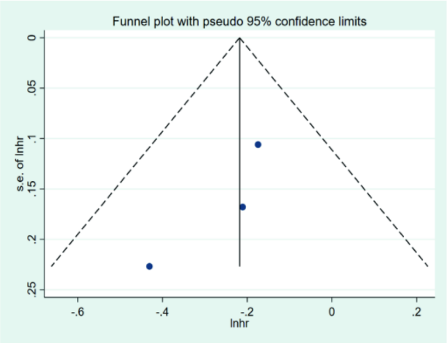

ECOG 1

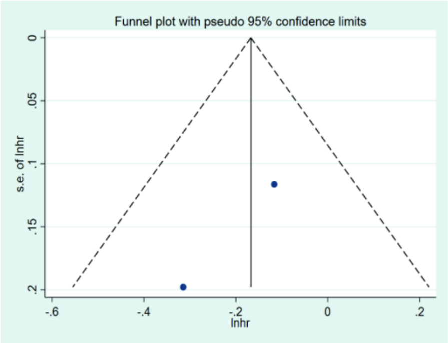

Supplementary Figure S14 Forest plots of ORR for chemoimmunotherapy vs. chemotherapy in mTNBC

A.ORR in the ITT population

Study or Subgroup	Chemo-IO Events	Chemo-IO Total	Chemo Events	Chemo Total	Weight	Risk Ratio M-H, Random, 95% CI
ALICE	11	40	5	28	4.1%	1.54 [0.60, 3.94]
IMpassion130	141	252	95	206	29.2%	1.21 [1.01, 1.46]
KEYNOTE-355	254	566	109	281	30.0%	1.16 [0.97, 1.38]
TBCRC 043	21	56	4	50	3.7%	4.69 [1.73, 12.73]
TORCHLIGHT	227	353	114	178	32.9%	1.00 [0.88, 1.15]
**Total (95% CI)**		**1267**		**743**	**100.0%**	**1.19 [0.97, 1.46]**
Total events	654		327			

Heterogeneity: Tau² = 0.03; Chi² = 12.39, df = 4 (P = 0.01); I² = 68%
Test for overall effect: Z = 1.71 (P = 0.09)

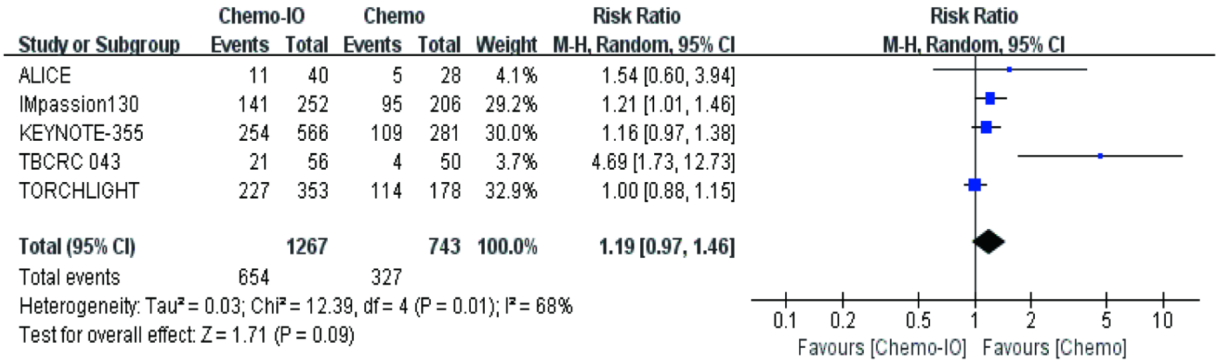

B.ORR in the PD-L1-positive population

Study or Subgroup	Chemo-IO Events	Chemo-IO Total	Chemo Events	Chemo Total	Weight	Risk Ratio M-H, Fixed, 95% CI
ALICE	6	21	2	10	1.1%	1.43 [0.35, 5.86]
IMpassion130	64	109	33	78	16.3%	1.39 [1.02, 1.88]
KEYNOTE-355	173	425	78	211	44.2%	1.10 [0.89, 1.36]
TORCHLIGHT	132	200	68	100	38.4%	0.97 [0.82, 1.15]
**Total (95% CI)**		**755**		**399**	**100.0%**	**1.10 [0.97, 1.25]**
Total events	375		181			

Heterogeneity: Chi² = 4.56, df = 3 (P = 0.21); I² = 34%
Test for overall effect: Z = 1.51 (P = 0.13)

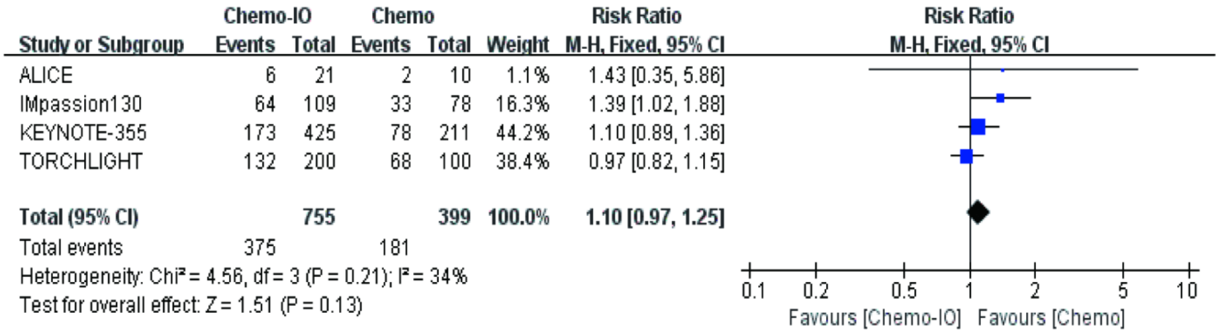

Supplementary Figure S15 The L'Abbe plot and Galbraith radial plot of ORR for chemoimmunotherapy vs. chemotherapy in mTNBC.

ITT

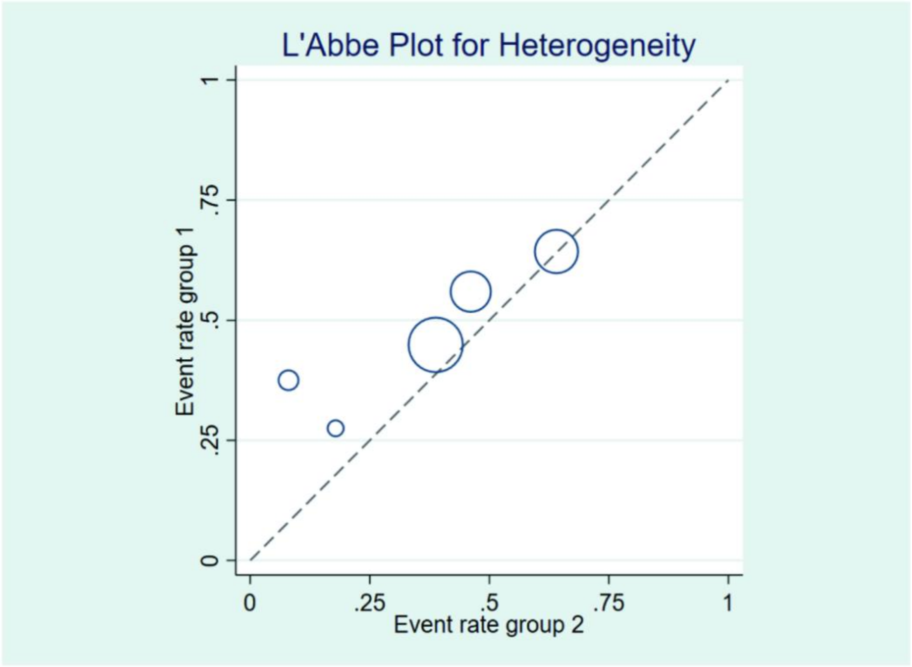

ITT

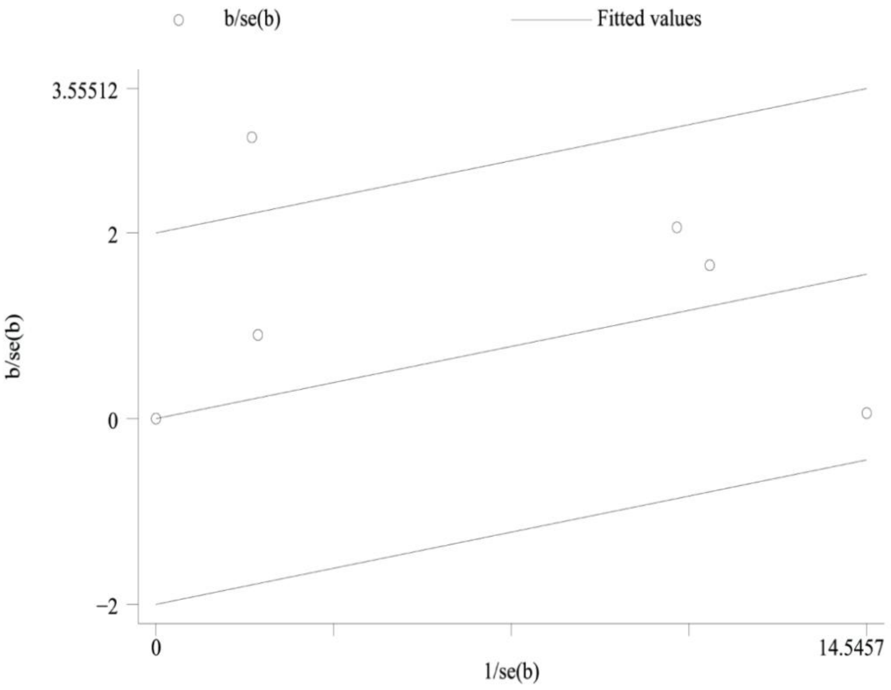

Supplementary Figure S16 The sensitivity analysis of ORR for chemoimmunotherapy vs. chemotherapy in mTNBC.

ITT

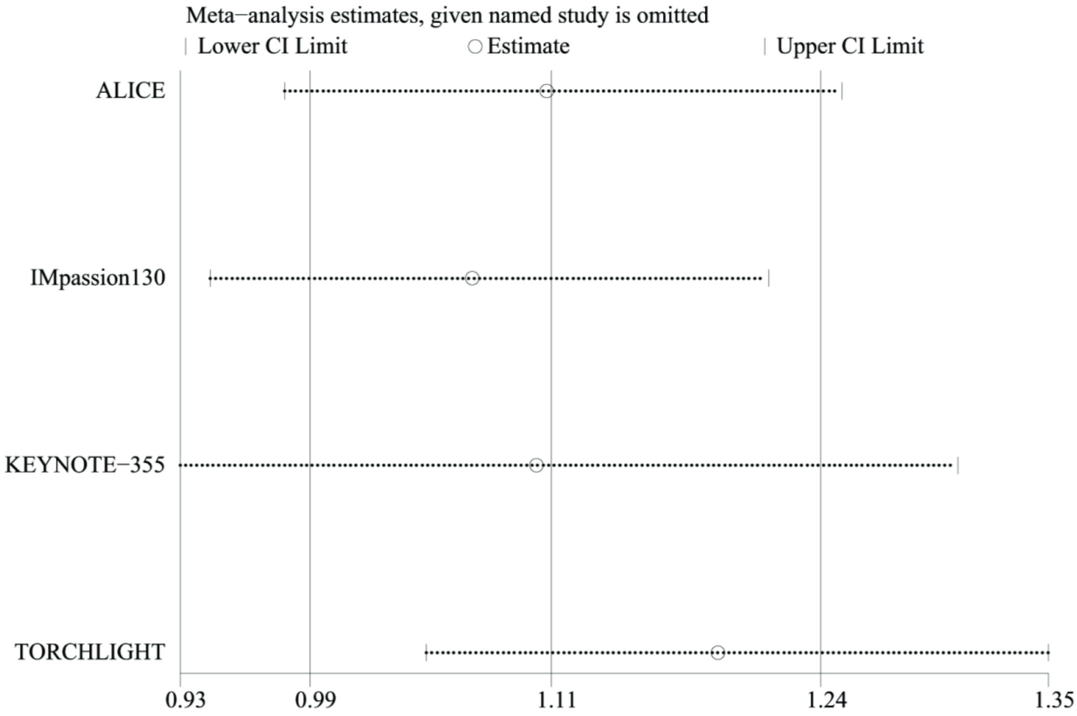

Supplementary Figure S17 The outcome of sensitivity analysis of ORR for chemoimmunotherapy vs. chemotherapy in mTNBC.

ITT

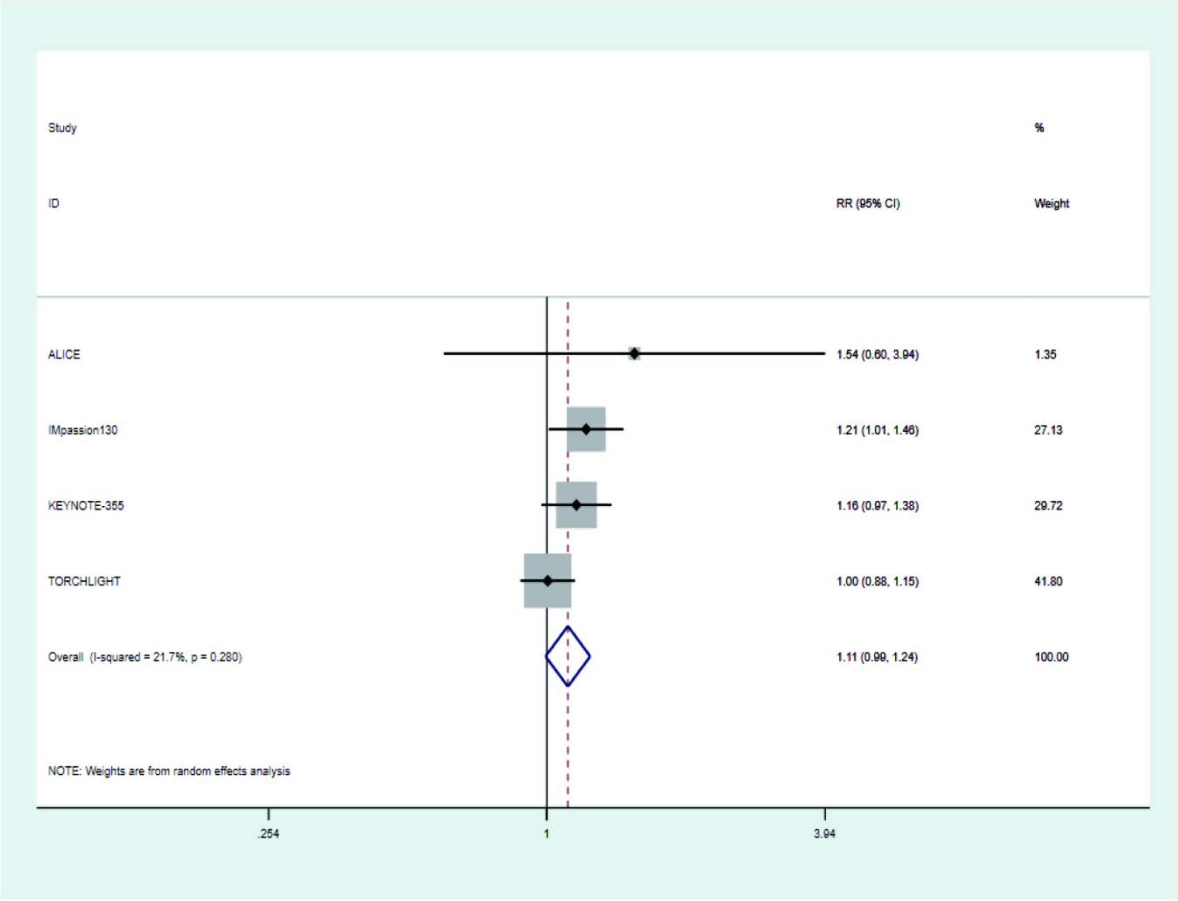

Supplementary Figure S18 The meta-regressions of ORR for chemoimmunotherapy vs. chemotherapy in mTNBC according to publication year and sample size.

ITT

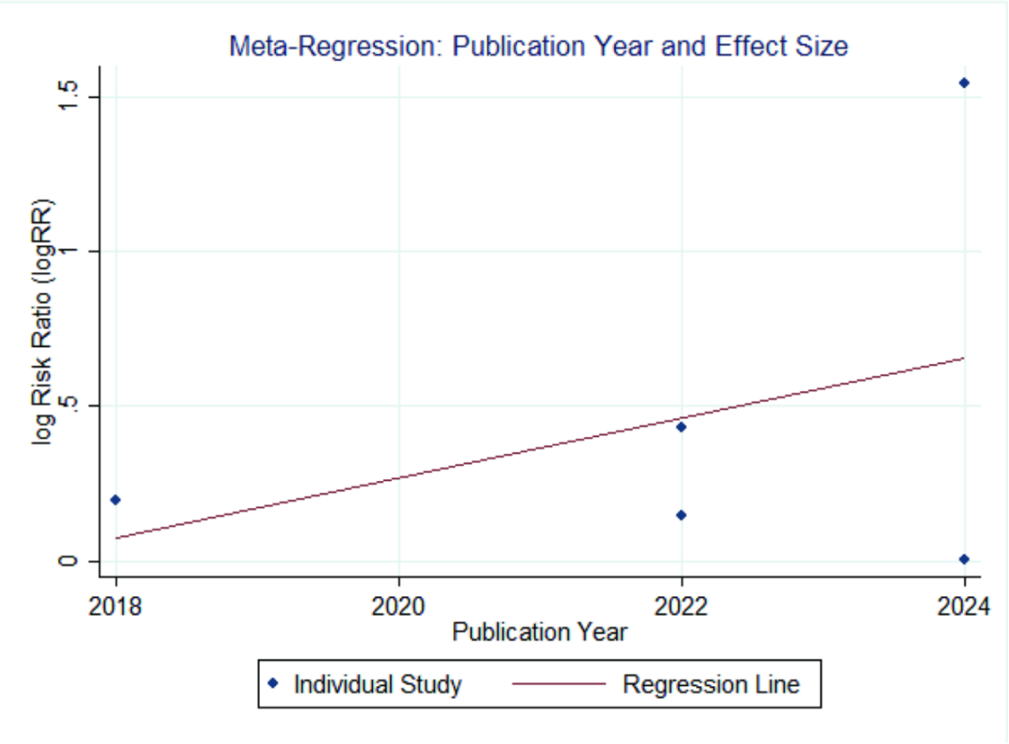

ITT

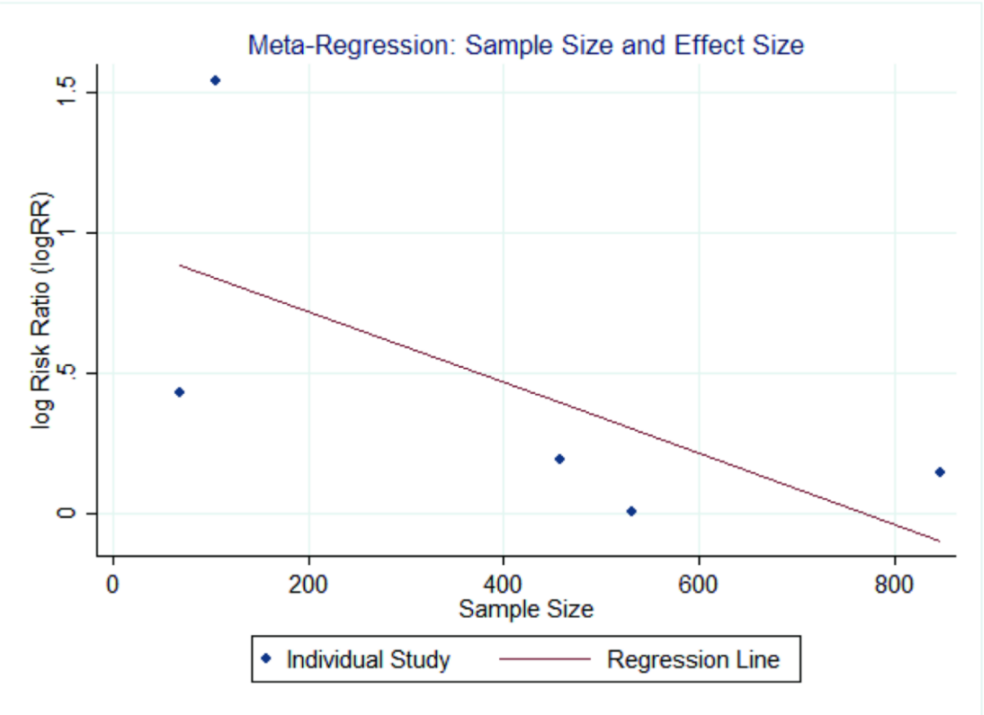

Supplementary Figure S19 Forest plots of CBR for chemoimmunotherapy vs. chemotherapy in mTNBC.

A.CBR in the ITT population

	Chemo-IO		Chemo			Risk Ratio
**Study or Subgroup**	**Events**	**Total**	**Events**	**Total**	**Weight**	**M-H, Fixed, 95% CI**
ALICE	20	40	10	28	4.2%	1.40 [0.78, 2.51]
IMpassion132	66	188	69	192	24.2%	0.98 [0.74, 1.28]
KEYNOTE-355	317	566	144	281	68.2%	1.09 [0.95, 1.25]
TBCRC 043	21	56	9	50	3.4%	2.08 [1.05, 4.12]
**Total (95% CI)**		**850**		**551**	**100.0%**	**1.11 [0.99, 1.25]**
Total events	424		232			

Heterogeneity: Chi² = 4.79, df = 3 (P = 0.19); I² = 37%
Test for overall effect: Z = 1.75 (P = 0.08)

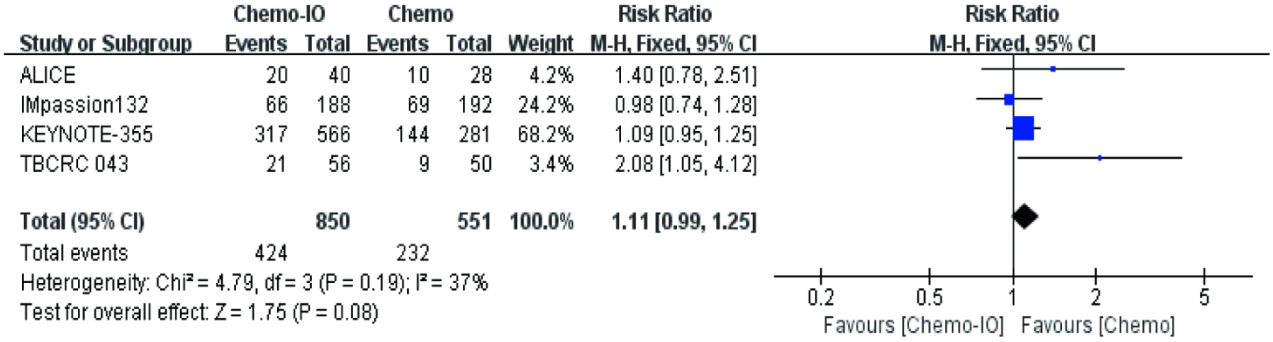

B.CBR in the PD-L1-positive population

	Chemo-IO		Chemo			Risk Ratio
**Study or Subgroup**	**Events**	**Total**	**Events**	**Total**	**Weight**	**M-H, Fixed, 95% CI**
ALICE	12	21	3	10	1.9%	1.90 [0.69, 5.26]
IMpassion132	76	177	62	177	28.6%	1.23 [0.94, 1.59]
KEYNOTE-355	249	425	113	211	69.6%	1.09 [0.94, 1.27]
**Total (95% CI)**		**623**		**398**	**100.0%**	**1.15 [1.01, 1.31]**
Total events	337		178			

Heterogeneity: Chi² = 1.59, df = 2 (P = 0.45); I² = 0%
Test for overall effect: Z = 2.07 (P = 0.04)

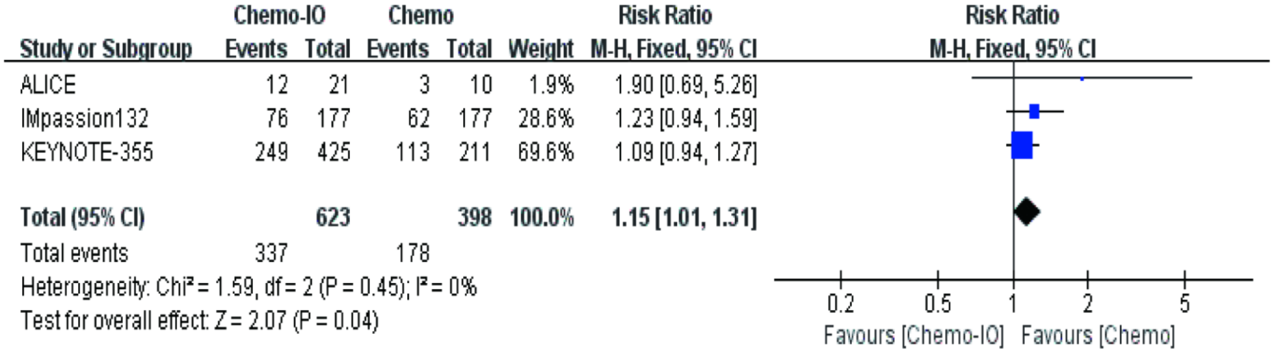

Supplementary Figure S20 The funnel plots of ORR for chemoimmunotherapy vs. chemotherapy in mTNBC.

ITT

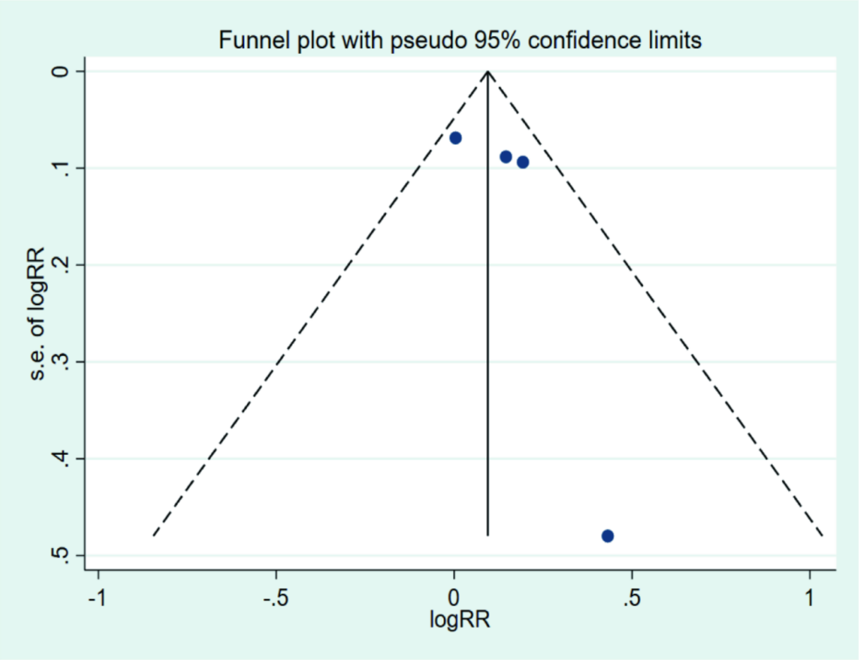

PD-L1-positive

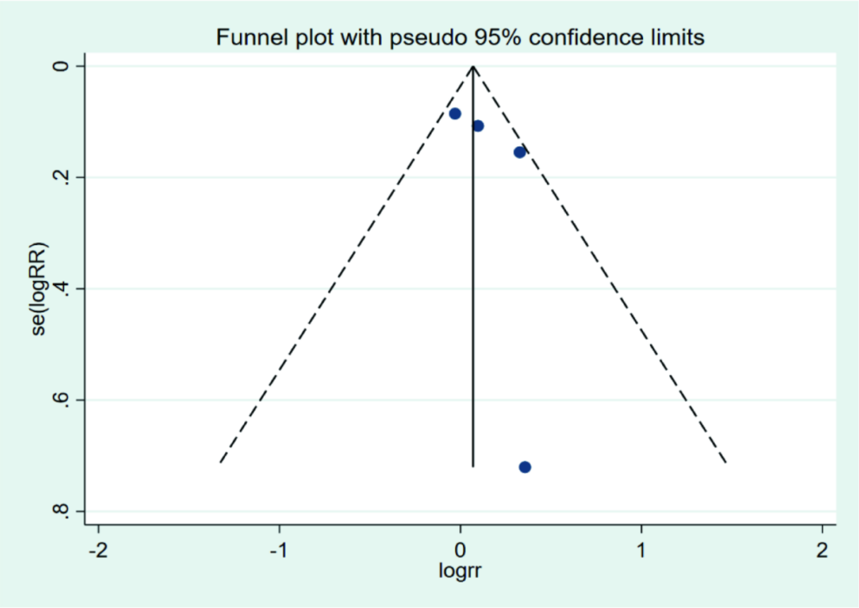

Supplementary Figure S21 The funnel plots of CBR for chemoimmunotherapy vs. chemotherapy in mTNBC.

ITT

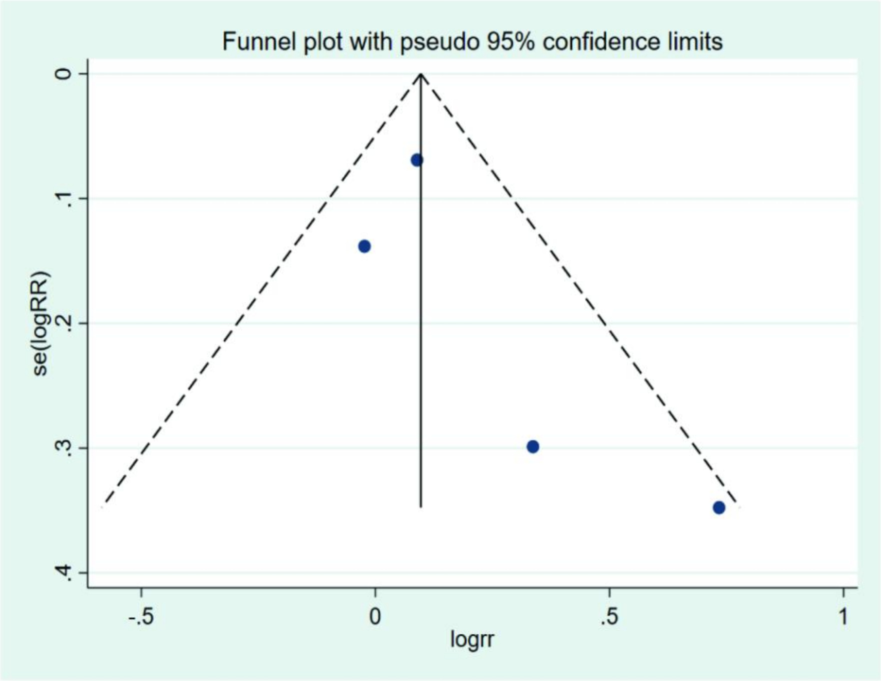

PD-L1-positive

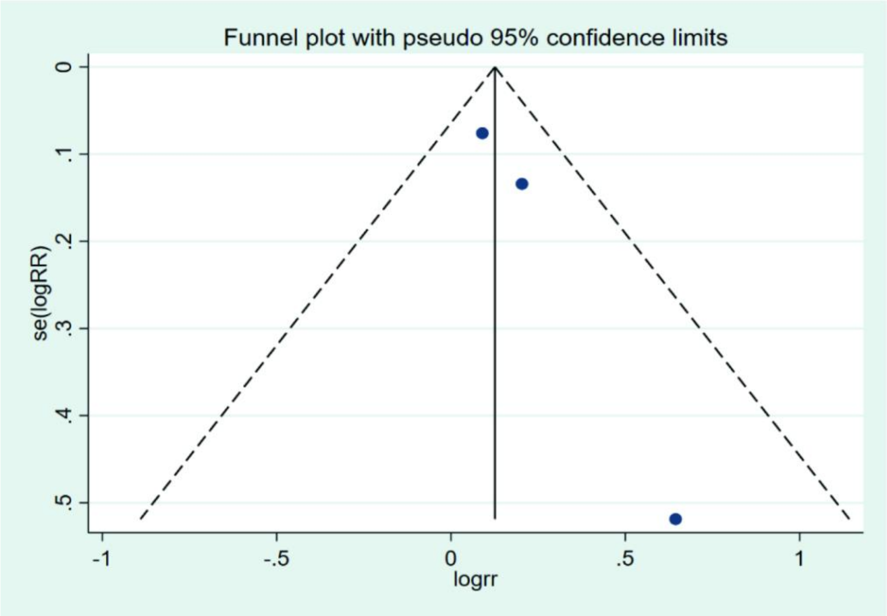

Supplementary Figure S22 Forest plots of safety outcomes for chemoimmunotherapy vs. chemotherapy in mTNBC.

A. AE

Study or Subgroup	Chemo-IO Events	Chemo-IO Total	Chemo Events	Chemo Total	Weight	Risk Ratio M-H, Fixed, 95% CI
ALICE	40	40	28	28	3.0%	1.00 [0.94, 1.06]
IMpassion130	449	452	429	438	38.9%	1.01 [1.00, 1.03]
IMpassion132	281	293	283	294	25.2%	1.00 [0.96, 1.03]
KEYNOTE-355	554	562	276	281	32.9%	1.00 [0.99, 1.02]
**Total (95% CI)**		**1347**		**1041**	**100.0%**	**1.01 [0.99, 1.02]**
Total events	1324		1016			

Heterogeneity: Chi^2 = 1.52, df = 3 (P = 0.68); I^2 = 0%
Test for overall effect: Z = 0.94 (P = 0.35)

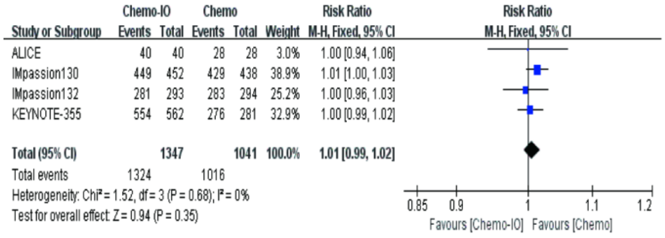

B. TEAE

Study or Subgroup	Chemo-IO Events	Chemo-IO Total	Chemo Events	Chemo Total	Weight	Risk Ratio M-H, Fixed, 95% CI
IMpassion130	436	452	410	438	32.7%	1.03 [1.00, 1.06]
IMpassion132	265	293	269	294	21.1%	0.99 [0.94, 1.04]
KEYNOTE-355	541	562	267	281	28.0%	1.01 [0.98, 1.05]
TORCHLIGHT	350	353	173	175	18.2%	1.00 [0.98, 1.02]
**Total (95% CI)**		**1660**		**1188**	**100.0%**	**1.01 [0.99, 1.03]**
Total events	1592		1119			

Heterogeneity: Chi^2 = 3.07, df = 3 (P = 0.38); I^2 = 2%
Test for overall effect: Z = 1.32 (P = 0.19)

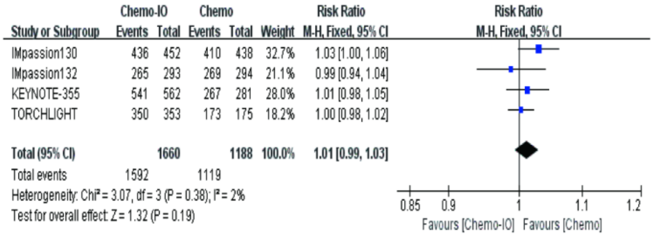

C. Grade ≥ 3 AE

Study or Subgroup	Chemo-IO Events	Chemo-IO Total	Chemo Events	Chemo Total	Weight	Risk Ratio M-H, Fixed, 95% CI
ALICE	25	40	12	28	2.0%	1.46 [0.89, 2.38]
IMpassion130	226	452	188	438	27.6%	1.16 [1.01, 1.34]
IMpassion132	199	293	212	294	30.6%	0.94 [0.85, 1.05]
KEYNOTE-355	438	562	207	281	39.8%	1.06 [0.97, 1.15]
**Total (95% CI)**		**1347**		**1041**	**100.0%**	**1.06 [1.00, 1.13]**
Total events	888		619			

Heterogeneity: Chi^2 = 8.10, df = 3 (P = 0.04); I^2 = 63%
Test for overall effect: Z = 1.85 (P = 0.06)

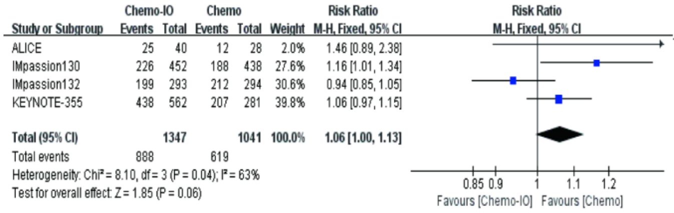

D. Grade ≥ 3 TEAE

Study or Subgroup	Chemo-IO Events	Chemo-IO Total	Chemo Events	Chemo Total	Weight	Risk Ratio M-H, Fixed, 95% CI
IMpassion130	182	452	133	438	19.2%	1.33 [1.11, 1.59]
IMpassion132	182	293	192	294	27.2%	0.95 [0.84, 1.07]
KEYNOTE-355	383	562	188	281	35.6%	1.02 [0.92, 1.13]
TORCHLIGHT	198	353	95	175	18.0%	1.03 [0.88, 1.22]
**Total (95% CI)**		**1660**		**1188**	**100.0%**	**1.06 [0.99, 1.14]**
Total events	945		608			

Heterogeneity: Chi^2 = 9.69, df = 3 (P = 0.02); I^2 = 69%
Test for overall effect: Z = 1.74 (P = 0.08)

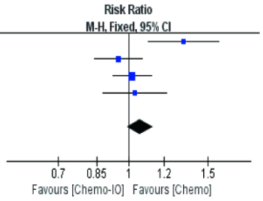

E. SAE

Study or Subgroup	Chemo-IO Events	Chemo-IO Total	Chemo Events	Chemo Total	Weight	Risk Ratio M-H, Fixed, 95% CI
ALICE	19	40	8	28	5.0%	1.66 [0.85, 3.25]
IMpassion130	103	452	80	438	43.3%	1.25 [0.96, 1.62]
IMpassion132	67	293	57	294	30.3%	1.18 [0.86, 1.61]
TORCHLIGHT	96	353	30	175	21.4%	1.59 [1.10, 2.29]
**Total (95% CI)**		**1138**		**935**	**100.0%**	**1.32 [1.11, 1.57]**
Total events	285		175			

Heterogeneity: Chi^2 = 2.09, df = 3 (P = 0.55); I^2 = 0%
Test for overall effect: Z = 3.20 (P = 0.001)

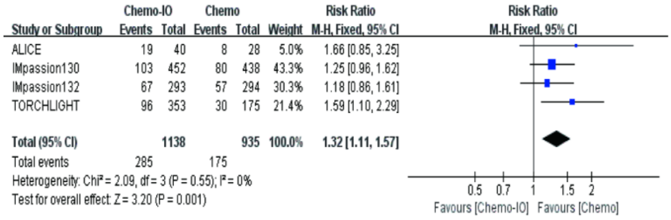

F. irAE

Study or Subgroup	Chemo-IO Events	Chemo-IO Total	Chemo Events	Chemo Total	Weight	Risk Ratio M-H, Fixed, 95% CI
ALICE	11	40	5	28	2.5%	1.54 [0.60, 3.94]
IMpassion131	268	432	116	217	64.4%	1.16 [1.00, 1.34]
KEYNOTE-355	144	562	17	281	9.5%	4.24 [2.62, 6.86]
TBCRC 043	12	56	0	50	0.2%	22.37 [1.36, 368.33]
TORCHLIGHT	144	353	42	175	23.4%	1.70 [1.27, 2.28]
**Total (95% CI)**		**1443**		**751**	**100.0%**	**1.63 [1.43, 1.86]**
Total events	579		180			

Heterogeneity: Chi^2 = 40.00, df = 4 (P < 0.00001); I^2 = 90%
Test for overall effect: Z = 7.29 (P < 0.00001)

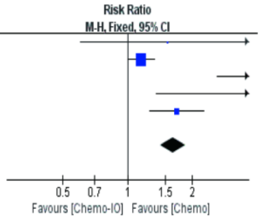

G. Grade ≥ 3 irAE

Study or Subgroup	Chemo-IO Events	Chemo-IO Total	Chemo Events	Chemo Total	Weight	Risk Ratio M-H, Fixed, 95% CI
ALICE	4	40	1	28	4.5%	2.80 [0.33, 23.74]
IMpassion131	49	432	11	217	55.5%	2.24 [1.19, 4.22]
KEYNOTE-355	29	562	0	281	2.5%	29.55 [1.81, 481.88]
TBCRC 043	3	56	0	50	2.0%	6.26 [0.33, 118.36]
TORCHLIGHT	53	353	7	175	35.5%	3.75 [1.74, 8.08]
**Total (95% CI)**		**1443**		**751**	**100.0%**	**3.57 [2.26, 5.65]**
Total events	138		19			

Heterogeneity: Chi^2 = 4.50, df = 4 (P = 0.34); I^2 = 11%
Test for overall effect: Z = 5.45 (P < 0.00001)

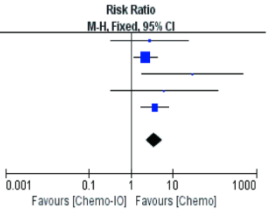

Supplementary Figure S23 The L'Abbe plot and Galbraith radial plot of safety outcomes for chemoimmunotherapy vs. chemotherapy in mTNBC.

Grade ≥3 AE

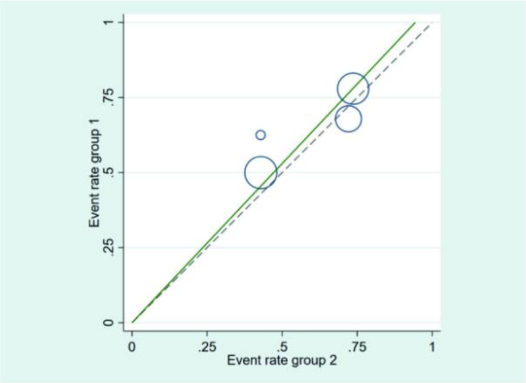

Grade ≥3 AE

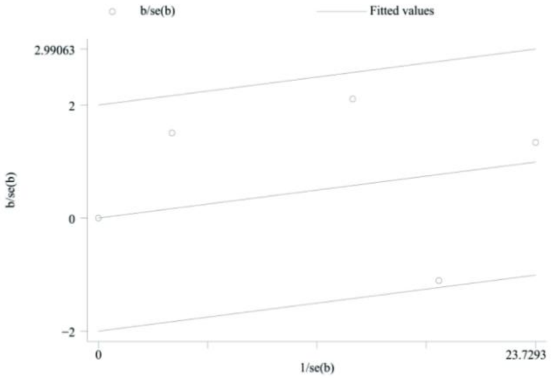

Grade ≥3 TEAE

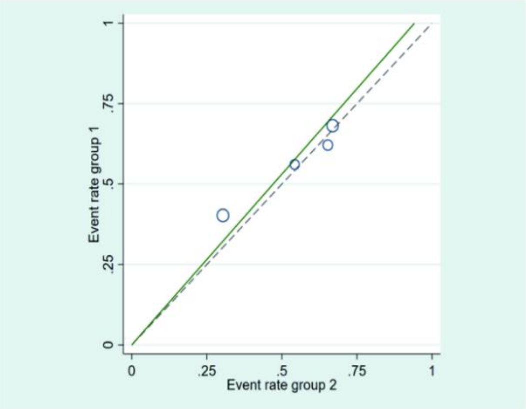

Grade ≥3 TEAE

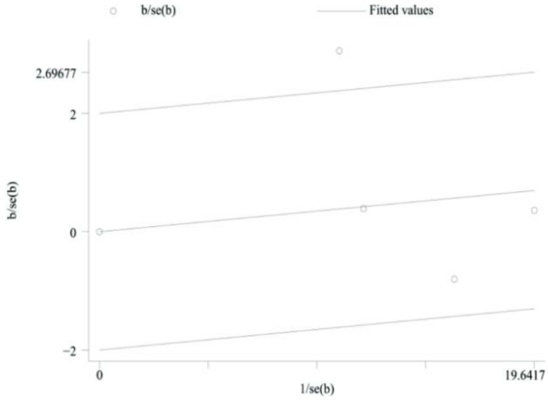

irAE

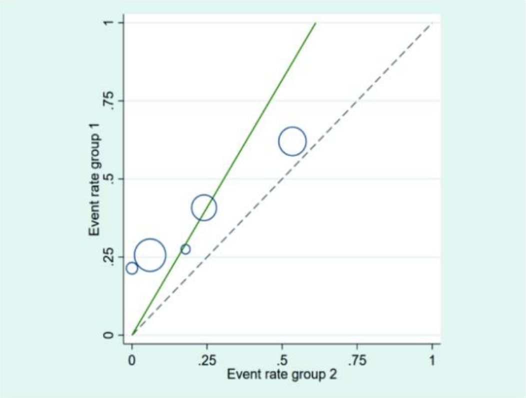

irAE

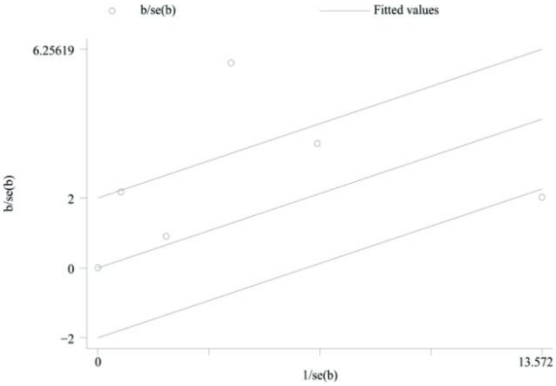

Supplementary Figure S24 The sensitivity analysis of safety outcomes for chemoimmunotherapy vs. chemotherapy in mTNBC.

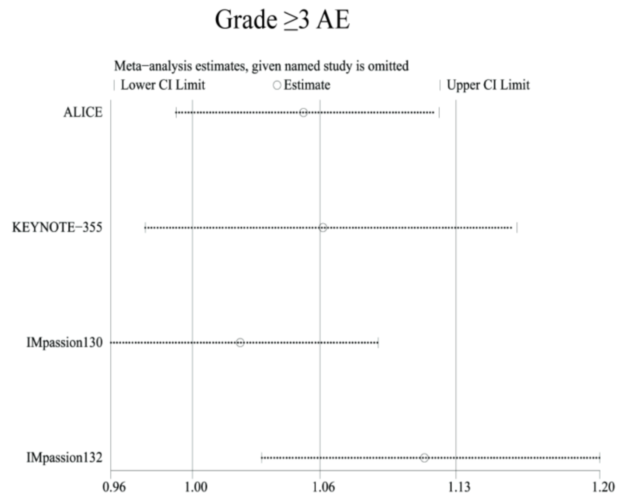

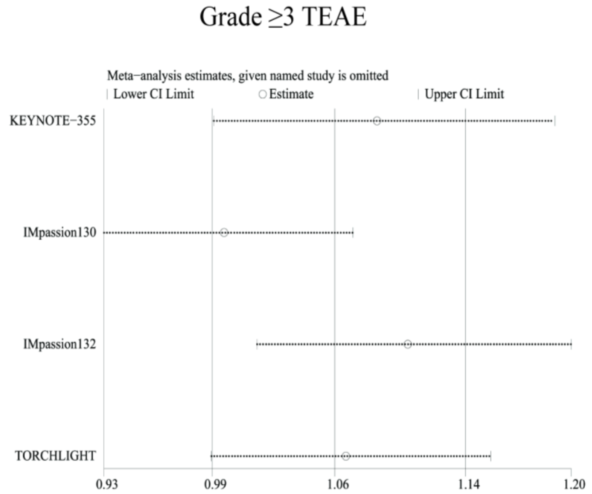

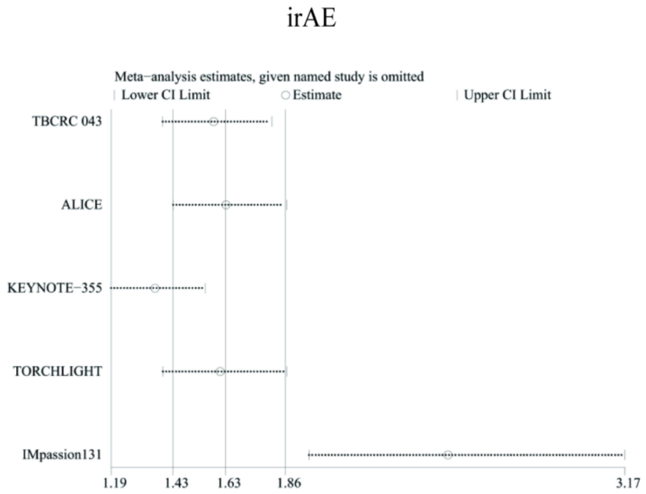

Supplementary Figure S25 The outcome of sensitivity analysis of safety outcomes for chemoimmunotherapy vs. chemotherapy in mTNBC.

Grade ≥3 AE

Study or Subgroup	Chemo-IO Events	Chemo-IO Total	Chemo Events	Chemo Total	Weight	Risk Ratio M-H, Fixed, 95% CI
ALICE	25	40	12	28	2.9%	1.46 [0.89, 2.38]
IMpassion130	226	452	188	438	39.7%	1.16 [1.01, 1.34]
KEYNOTE-355	438	562	207	281	57.4%	1.06 [0.97, 1.15]
**Total (95% CI)**		**1054**		**747**	**100.0%**	**1.11 [1.03, 1.20]**
Total events	689		407			

Heterogeneity: Chi² = 2.99, df = 2 (P = 0.22); I² = 33%
Test for overall effect: Z = 2.75 (P = 0.006)

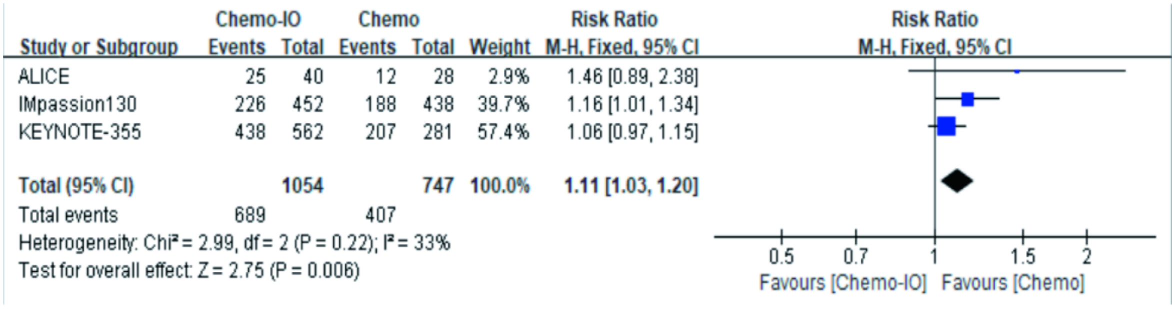

Grade ≥3 TEAE

Study or Subgroup	Chemo-IO Events	Chemo-IO Total	Chemo Events	Chemo Total	Weight	Risk Ratio M-H, Fixed, 95% CI
IMpassion132	182	293	192	294	33.7%	0.95 [0.84, 1.07]
KEYNOTE-355	383	562	188	281	44.0%	1.02 [0.92, 1.13]
TORCHLIGHT	198	353	95	175	22.3%	1.03 [0.88, 1.22]
**Total (95% CI)**		**1208**		**750**	**100.0%**	**1.00 [0.93, 1.07]**
Total events	763		475			

Heterogeneity: Chi² = 0.93, df = 2 (P = 0.63); I² = 0%
Test for overall effect: Z = 0.02 (P = 0.98)

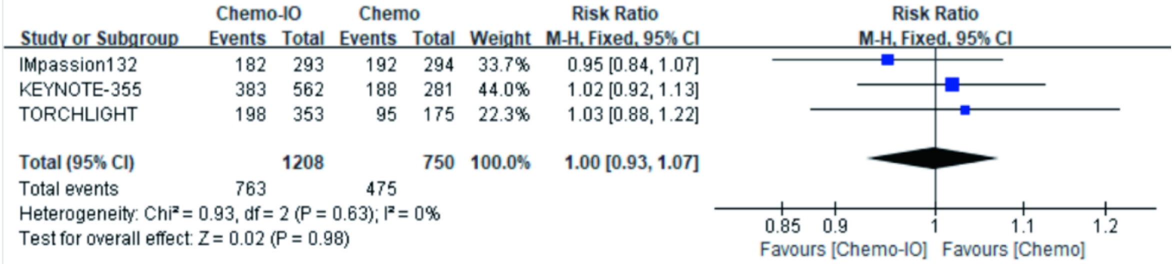

irAE

Study or Subgroup	Chemo-IO Events	Chemo-IO Total	Chemo Events	Chemo Total	Weight	Risk Ratio M-H, Fixed, 95% CI
ALICE	11	40	5	28	9.4%	1.54 [0.60, 3.94]
TBCRC 043	12	56	0	50	0.8%	22.37 [1.36, 368.33]
TORCHLIGHT	144	353	42	175	89.8%	1.70 [1.27, 2.28]
**Total (95% CI)**		**449**		**253**	**100.0%**	**1.86 [1.41, 2.45]**
Total events	167		47			

Heterogeneity: Chi² = 3.54, df = 2 (P = 0.17); I² = 44%
Test for overall effect: Z = 4.41 (P < 0.0001)

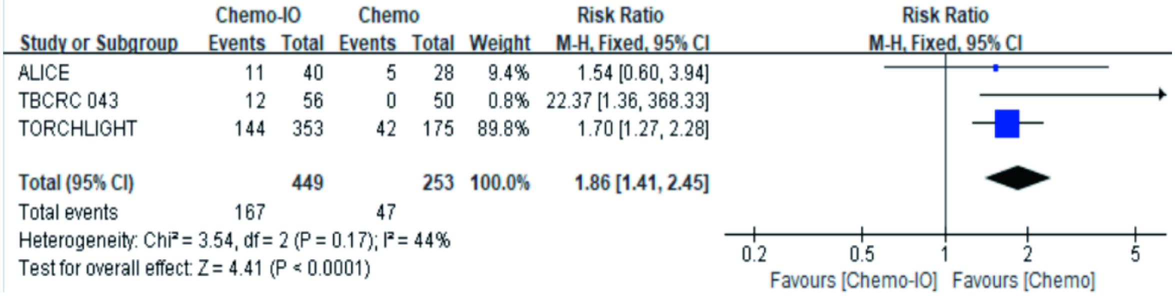

Supplementary Figure S26 The meta-regressions of safety outcomes for chemoimmunotherapy vs. chemotherapy in mTNBC according to publication year and sample size.

Grade ≥ 3 AE

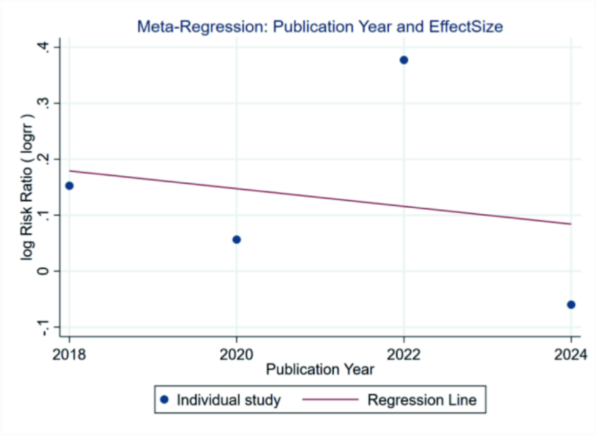

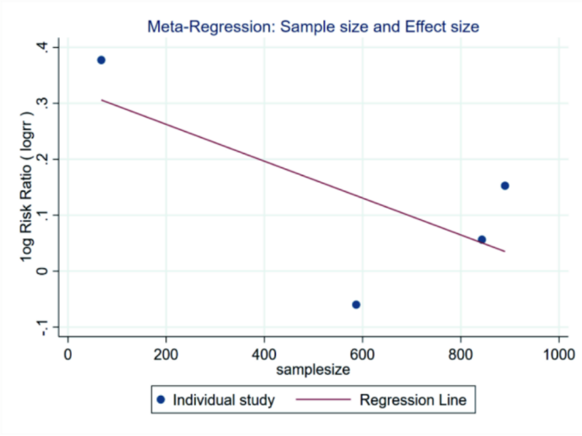

Grade ≥ 3 TEAE

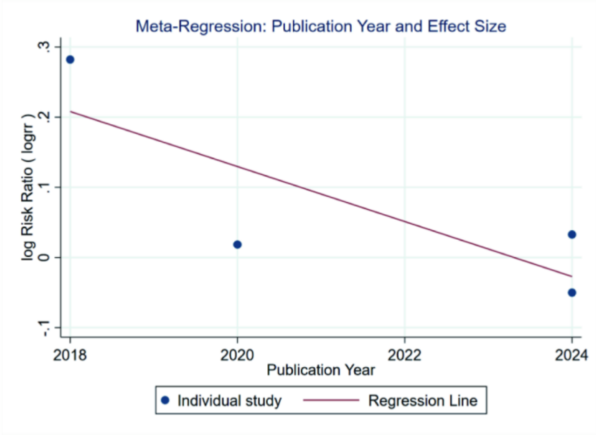

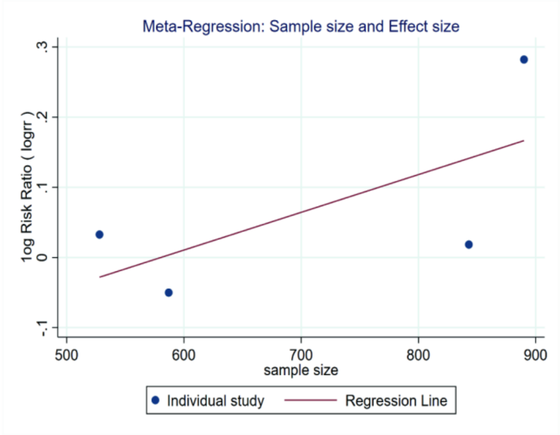

irAE

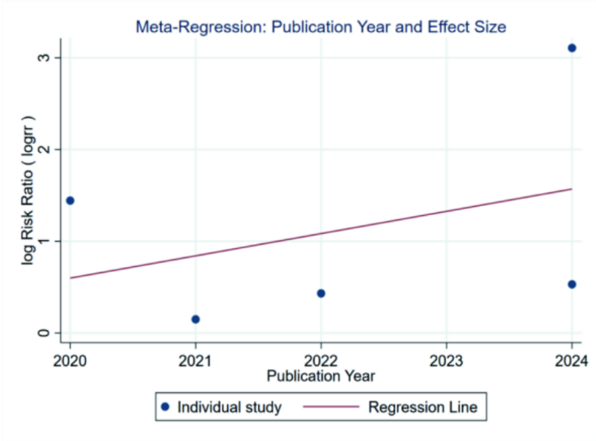

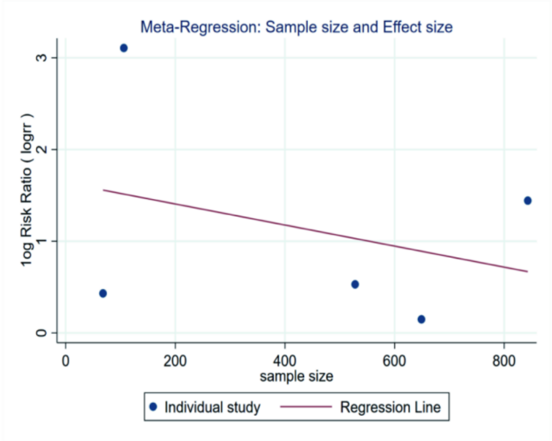

Supplementary Figure S27 The funnel plots of safety outcomes for chemoimmunotherapy vs. chemotherapy in mTNBC.

AE

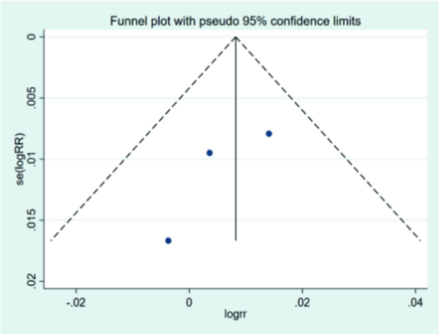

TEAE

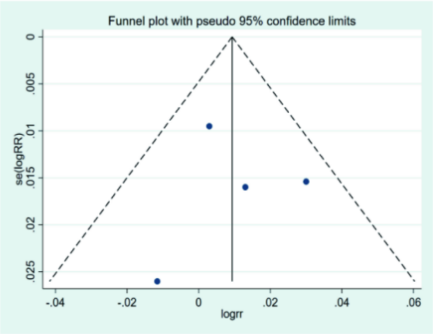

Grade ≥ 3 AE

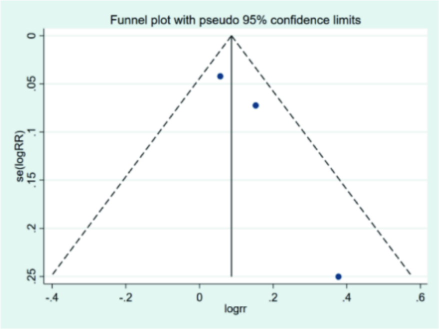

Grade ≥ 3 TEAE

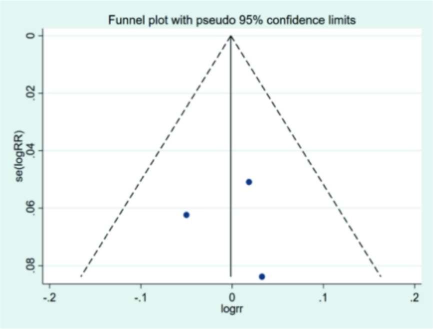

SAE

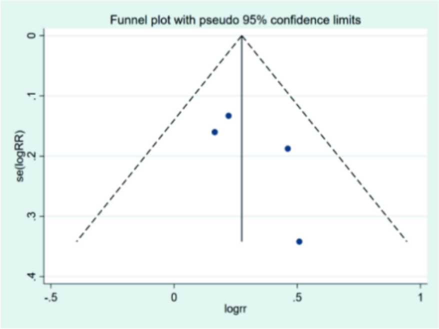

irAE

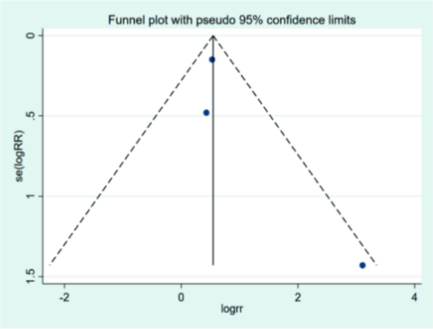

Grade ≥ 3 irAE

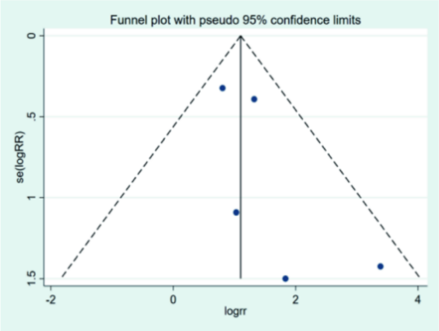

Supplementary Figure S28 Forest plots of the incidence of adverse events for chemoimmunotherapy versus chemotherapy in mTNBC.

Rash

Study or Subgroup	Chemo-IO Events	Chemo-IO Total	Chemo Events	Chemo Total	Weight	Risk Ratio M-H, Fixed, 95% CI
ALICE	26	40	11	28	9.8%	1.65 [0.99, 2.77]
IMpassion130	78	452	72	438	55.1%	1.05 [0.78, 1.41]
IMpassion131	78	432	35	217	35.1%	1.12 [0.78, 1.61]
**Total (95% CI)**		**924**		**683**	**100.0%**	**1.13 [0.92, 1.40]**
Total events	182		118			

Heterogeneity: Chi² = 2.35, df = 2 (P = 0.31); I² = 15%
Test for overall effect: Z = 1.17 (P = 0.24)

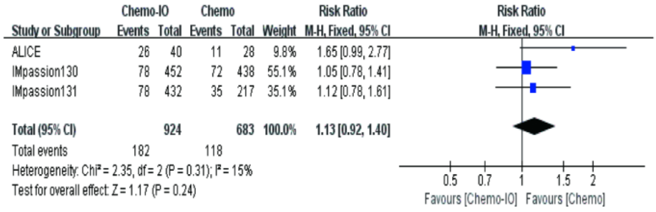

Fatigue

Study or Subgroup	Chemo-IO Events	Chemo-IO Total	Chemo Events	Chemo Total	Weight	Risk Ratio M-H, Fixed, 95% CI
ALICE	20	40	12	28	4.0%	1.17 [0.69, 1.98]
IMpassion130	211	452	196	438	56.7%	1.04 [0.90, 1.20]
IMpassion131	119	432	57	217	21.6%	1.05 [0.80, 1.37]
IMpassion132	69	293	62	294	17.6%	1.12 [0.83, 1.51]
**Total (95% CI)**		**1217**		**977**	**100.0%**	**1.06 [0.95, 1.19]**
Total events	419		327			

Heterogeneity: Chi² = 0.30, df = 3 (P = 0.96); I² = 0%
Test for overall effect: Z = 1.03 (P = 0.31)

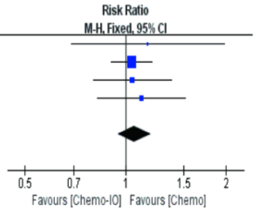

Asthenia

Study or Subgroup	Chemo-IO Events	Chemo-IO Total	Chemo Events	Chemo Total	Weight	Risk Ratio M-H, Fixed, 95% CI
IMpassion130	56	452	50	438	27.3%	1.09 [0.76, 1.55]
IMpassion131	101	432	47	217	33.6%	1.08 [0.80, 1.46]
IMpassion132	66	293	73	294	39.1%	0.91 [0.68, 1.21]
**Total (95% CI)**		**1177**		**949**	**100.0%**	**1.01 [0.85, 1.22]**
Total events	223		170			

Heterogeneity: Chi² = 0.86, df = 2 (P = 0.65); I² = 0%
Test for overall effect: Z = 0.15 (P = 0.88)

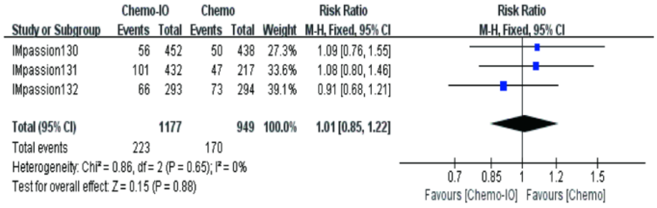

Constipation

Study or Subgroup	Chemo-IO Events	Chemo-IO Total	Chemo Events	Chemo Total	Weight	Risk Ratio M-H, Fixed, 95% CI
ALICE	18	40	12	28	6.2%	1.05 [0.61, 1.82]
IMpassion130	113	452	108	438	48.0%	1.01 [0.81, 1.27]
IMpassion131	81	432	33	217	19.2%	1.23 [0.85, 1.79]
IMpassion132	61	293	61	294	26.6%	1.00 [0.73, 1.38]
**Total (95% CI)**		**1217**		**977**	**100.0%**	**1.06 [0.90, 1.24]**
Total events	273		214			

Heterogeneity: Chi² = 0.89, df = 3 (P = 0.83); I² = 0%
Test for overall effect: Z = 0.66 (P = 0.51)

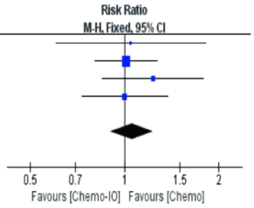

Vomiting

Study or Subgroup	Chemo-IO Events	Chemo-IO Total	Chemo Events	Chemo Total	Weight	Risk Ratio M-H, Fixed, 95% CI
IMpassion130	88	452	74	438	43.4%	1.15 [0.87, 1.52]
IMpassion131	69	432	21	217	16.2%	1.65 [1.04, 2.62]
IMpassion132	67	293	70	294	40.4%	0.96 [0.72, 1.29]
**Total (95% CI)**		**1177**		**949**	**100.0%**	**1.16 [0.96, 1.39]**
Total events	224		165			

Heterogeneity: Chi² = 3.83, df = 2 (P = 0.15); I² = 48%
Test for overall effect: Z = 1.53 (P = 0.13)

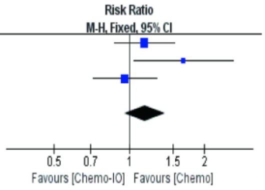

Nausea

Study or Subgroup	Chemo-IO Events	Chemo-IO Total	Chemo Events	Chemo Total	Weight	Risk Ratio M-H, Fixed, 95% CI
ALICE	23	40	15	28	4.6%	1.07 [0.69, 1.66]
IMpassion130	208	452	167	438	44.5%	1.21 [1.03, 1.41]
IMpassion131	116	432	54	217	18.9%	1.08 [0.82, 1.43]
IMpassion132	121	293	122	294	32.0%	1.00 [0.82, 1.21]
**Total (95% CI)**		**1217**		**977**	**100.0%**	**1.11 [1.00, 1.24]**
Total events	468		358			

Heterogeneity: Chi² = 2.41, df = 3 (P = 0.49); I² = 0%
Test for overall effect: Z = 1.87 (P = 0.06)

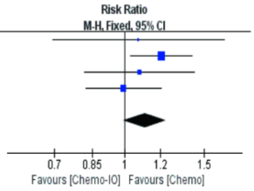

Anaemia

Study or Subgroup	Chemo-IO Events	Chemo-IO Total	Chemo Events	Chemo Total	Weight	Risk Ratio M-H, Fixed, 95% CI
IMpassion130	125	452	115	438	33.7%	1.05 [0.85, 1.31]
IMpassion131	128	432	68	217	26.1%	0.95 [0.74, 1.21]
IMpassion132	135	293	140	294	40.3%	0.97 [0.81, 1.15]
**Total (95% CI)**		**1177**		**949**	**100.0%**	**0.99 [0.88, 1.12]**
Total events	388		323			

Heterogeneity: Chi² = 0.52, df = 2 (P = 0.77); I² = 0%
Test for overall effect: Z = 0.15 (P = 0.88)

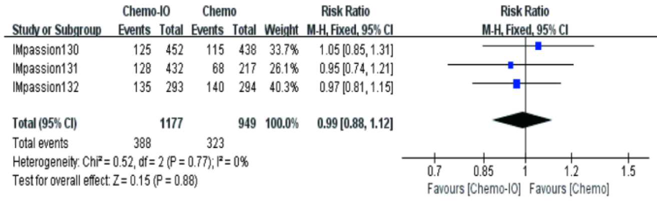

Alanineaminotransferase increased

Study or Subgroup	Chemo-IO Events	Chemo-IO Total	Chemo Events	Chemo Total	Weight	Risk Ratio M-H, Fixed, 95% CI
IMpassion130	47	452	40	438	21.3%	1.14 [0.76, 1.70]
IMpassion131	84	432	37	217	25.8%	1.14 [0.80, 1.62]
IMpassion132	94	293	101	294	52.9%	0.93 [0.74, 1.17]
**Total (95% CI)**		**1177**		**949**	**100.0%**	**1.03 [0.87, 1.23]**
Total events	225		178			

Heterogeneity: Chi² = 1.27, df = 2 (P = 0.53); I² = 0%
Test for overall effect: Z = 0.34 (P = 0.73)

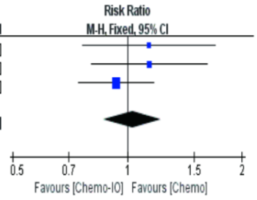

Neutrophil count decreased

Study or Subgroup	Chemo-IO Events	Chemo-IO Total	Chemo Events	Chemo Total	Weight	Risk Ratio M-H, Fixed, 95% CI
IMpassion130	57	452	48	438	26.6%	1.15 [0.80, 1.65]
IMpassion131	67	432	35	217	25.4%	0.96 [0.66, 1.40]
IMpassion132	76	293	88	294	48.0%	0.87 [0.67, 1.12]
**Total (95% CI)**		**1177**		**949**	**100.0%**	**0.97 [0.80, 1.16]**
Total events	200		171			

Heterogeneity: Chi² = 1.57, df = 2 (P = 0.46); I² = 0%
Test for overall effect: Z = 0.36 (P = 0.72)

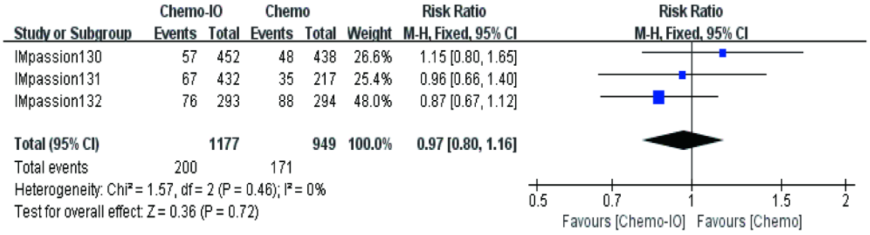

Supplementary Figure S29 Forest plots of the incidence of treatment-emergent adverse events for chemoimmunotherapy versus chemotherapy in mTNBC.

Fatigue

	Chemo-IO		Chemo			Risk Ratio
Study or Subgroup	Events	Total	Events	Total	Weight	M-H, Fixed, 95% CI
IMpassion130	181	452	167	438	60.3%	1.05 [0.89, 1.24]
KEYNOTE-355	160	562	83	281	39.3%	0.96 [0.77, 1.20]
TBCRC 043	11	56	1	50	0.4%	9.82 [1.31, 73.39]
**Total (95% CI)**		**1070**		**769**	**100.0%**	**1.05 [0.92, 1.20]**
Total events	352		251			

Heterogeneity: Chi² = 5.31, df = 2 (P = 0.07); I² = 62%
Test for overall effect: Z = 0.71 (P = 0.48)

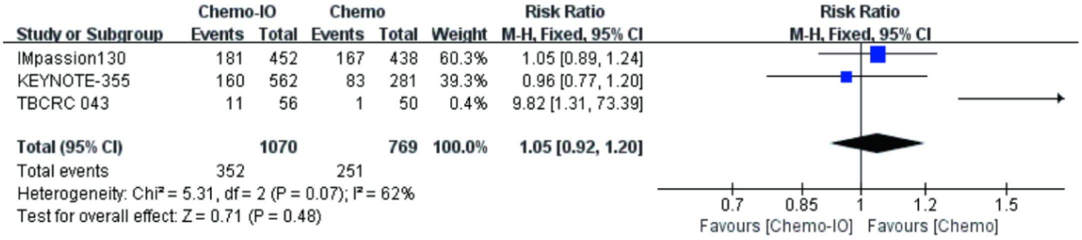

Nausea

	Chemo-IO		Chemo			Risk Ratio
Study or Subgroup	Events	Total	Events	Total	Weight	M-H, Fixed, 95% CI
IMpassion130	186	452	148	438	44.1%	1.22 [1.03, 1.45]
KEYNOTE-355	221	562	115	281	45.0%	0.96 [0.81, 1.14]
TBCRC 043	5	56	1	50	0.3%	4.46 [0.54, 36.93]
TORCHLIGHT	94	353	27	175	10.6%	1.73 [1.17, 2.54]
**Total (95% CI)**		**1423**		**944**	**100.0%**	**1.17 [1.04, 1.31]**
Total events	506		291			

Heterogeneity: Chi² = 10.48, df = 3 (P = 0.01); I² = 71%
Test for overall effect: Z = 2.58 (P = 0.010)

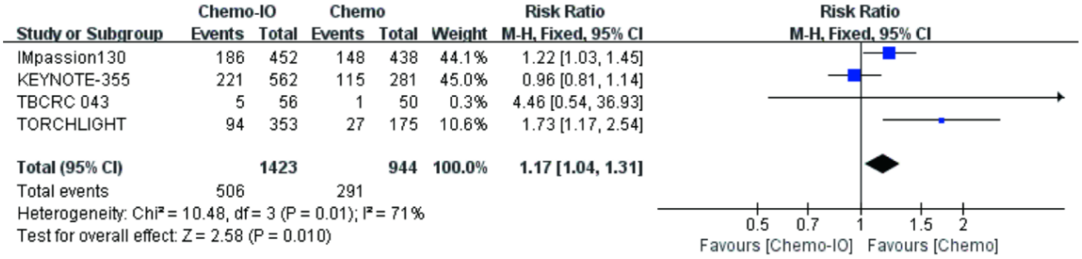

Anaemia

	Chemo-IO		Chemo			Risk Ratio
Study or Subgroup	Events	Total	Events	Total	Weight	M-H, Fixed, 95% CI
IMpassion130	112	452	99	438	24.8%	1.10 [0.87, 1.39]
KEYNOTE-355	275	562	129	281	42.4%	1.07 [0.92, 1.24]
TBCRC 043	17	56	0	50	0.1%	31.32 [1.93, 507.63]
TORCHLIGHT	215	353	99	175	32.6%	1.08 [0.92, 1.26]
**Total (95% CI)**		**1423**		**944**	**100.0%**	**1.12 [1.01, 1.23]**
Total events	619		327			

Heterogeneity: Chi² = 6.09, df = 3 (P = 0.11); I² = 51%
Test for overall effect: Z = 2.14 (P = 0.03)

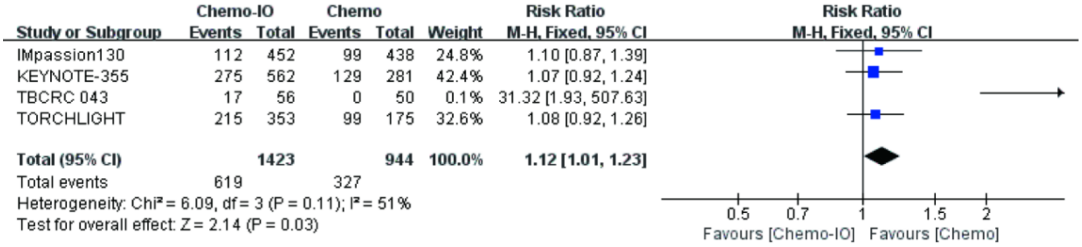

Alopecia

	Chemo-IO		Chemo			Risk Ratio
Study or Subgroup	Events	Total	Events	Total	Weight	M-H, Fixed, 95% CI
IMpassion130	253	452	251	438	48.6%	0.98 [0.87, 1.10]
KEYNOTE-355	186	562	94	281	23.9%	0.99 [0.81, 1.21]
TORCHLIGHT	238	353	108	175	27.5%	1.09 [0.95, 1.25]
**Total (95% CI)**		**1367**		**894**	**100.0%**	**1.01 [0.93, 1.10]**
Total events	677		453			

Heterogeneity: Chi² = 1.61, df = 2 (P = 0.45); I² = 0%
Test for overall effect: Z = 0.27 (P = 0.78)

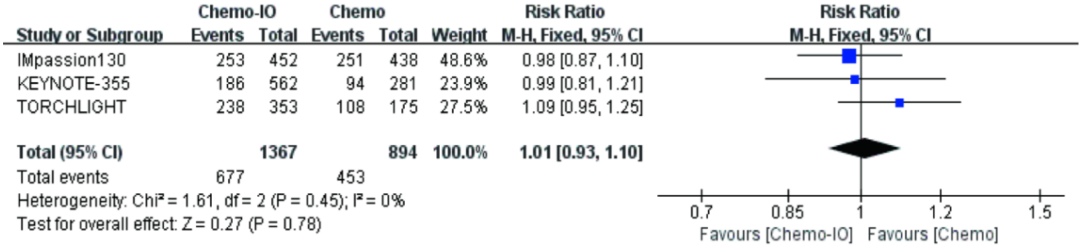

Neutropenia

	Chemo-IO		Chemo			Risk Ratio
Study or Subgroup	Events	Total	Events	Total	Weight	M-H, Fixed, 95% CI
IMpassion130	93	452	66	438	18.5%	1.37 [1.03, 1.82]
KEYNOTE-355	231	562	107	281	39.5%	1.08 [0.90, 1.29]
TBCRC 043	16	56	2	50	0.6%	7.14 [1.73, 29.54]
TORCHLIGHT	239	353	112	175	41.4%	1.06 [0.93, 1.21]
**Total (95% CI)**		**1423**		**944**	**100.0%**	**1.16 [1.04, 1.29]**
Total events	579		287			

Heterogeneity: Chi² = 9.99, df = 3 (P = 0.02); I² = 70%
Test for overall effect: Z = 2.74 (P = 0.006)

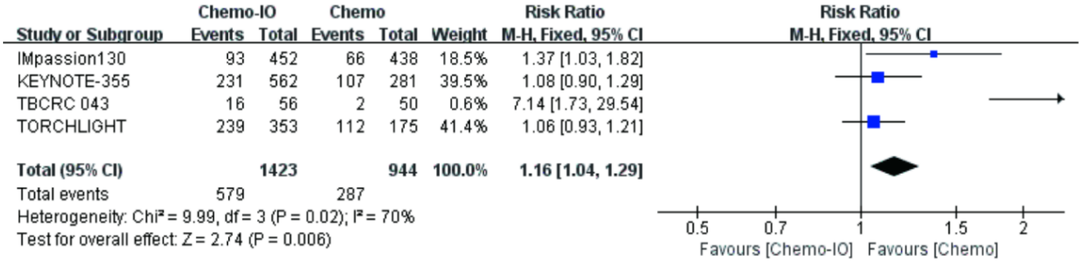

Supplementary Figure S30 Forest plots of the incidence of treatment-emergent adverse events for chemoimmunotherapy versus chemotherapy in mTNBC, adjusted for heterogeneity.

Fatigue

Study or Subgroup	Chemo-IO Events	Chemo-IO Total	Chemo Events	Chemo Total	Weight	Risk Ratio M-H, Fixed, 95% CI
IMpassion130	181	452	167	438	60.5%	1.05 [0.89, 1.24]
KEYNOTE-355	160	562	83	281	39.5%	0.96 [0.77, 1.20]
**Total (95% CI)**		**1014**		**719**	**100.0%**	**1.02 [0.89, 1.16]**
Total events	341		250			

Heterogeneity: Chi² = 0.37, df = 1 (P = 0.54); I² = 0%
Test for overall effect: Z = 0.24 (P = 0.81)

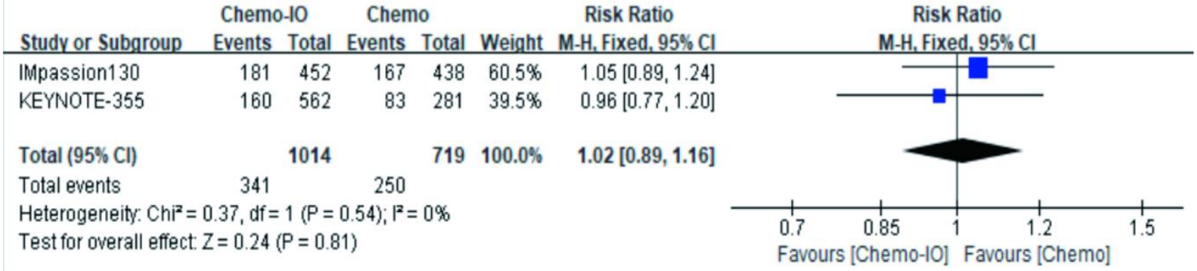

Nausea

Study or Subgroup	Chemo-IO Events	Chemo-IO Total	Chemo Events	Chemo Total	Weight	Risk Ratio M-H, Fixed, 95% CI
TBCRC 043	5	56	1	50	2.8%	4.46 [0.54, 36.93]
TORCHLIGHT	94	353	27	175	97.2%	1.73 [1.17, 2.54]
**Total (95% CI)**		**409**		**225**	**100.0%**	**1.80 [1.23, 2.64]**
Total events	99		28			

Heterogeneity: Chi² = 0.76, df = 1 (P = 0.38); I² = 0%
Test for overall effect: Z = 3.04 (P = 0.002)

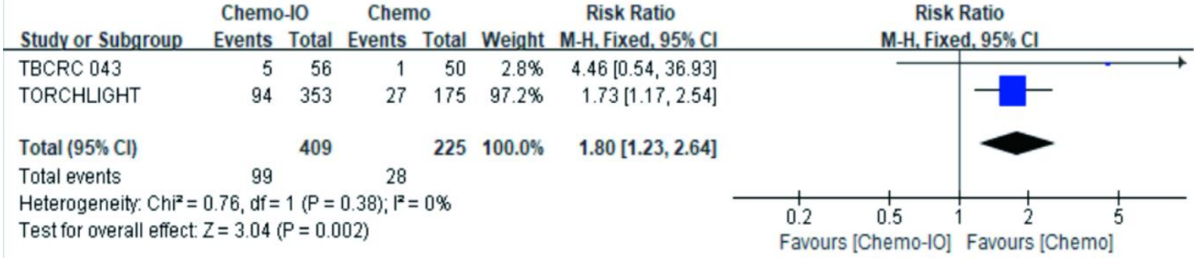

Anaemia

Study or Subgroup	Chemo-IO Events	Chemo-IO Total	Chemo Events	Chemo Total	Weight	Risk Ratio M-H, Fixed, 95% CI
IMpassion130	112	452	99	438	24.8%	1.10 [0.87, 1.39]
KEYNOTE-355	275	562	129	281	42.5%	1.07 [0.92, 1.24]
TORCHLIGHT	215	353	99	175	32.7%	1.08 [0.92, 1.26]
**Total (95% CI)**		**1367**		**894**	**100.0%**	**1.08 [0.97, 1.19]**
Total events	602		327			

Heterogeneity: Chi² = 0.04, df = 2 (P = 0.98); I² = 0%
Test for overall effect: Z = 1.44 (P = 0.15)

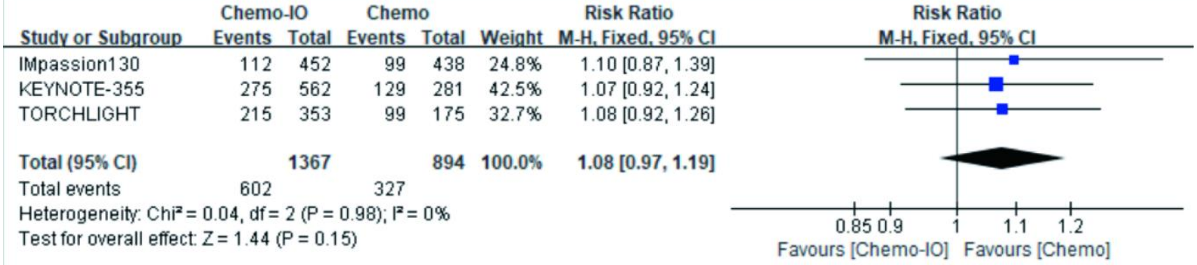

Neutropenia

Study or Subgroup	Chemo-IO Events	Chemo-IO Total	Chemo Events	Chemo Total	Weight	Risk Ratio M-H, Fixed, 95% CI
IMpassion130	93	452	66	438	18.6%	1.37 [1.03, 1.82]
KEYNOTE-355	231	562	107	281	39.7%	1.08 [0.90, 1.29]
TORCHLIGHT	239	353	112	175	41.7%	1.06 [0.93, 1.21]
**Total (95% CI)**		**1367**		**894**	**100.0%**	**1.12 [1.01, 1.25]**
Total events	563		285			

Heterogeneity: Chi² = 2.77, df = 2 (P = 0.25); I² = 28%
Test for overall effect: Z = 2.16 (P = 0.03)

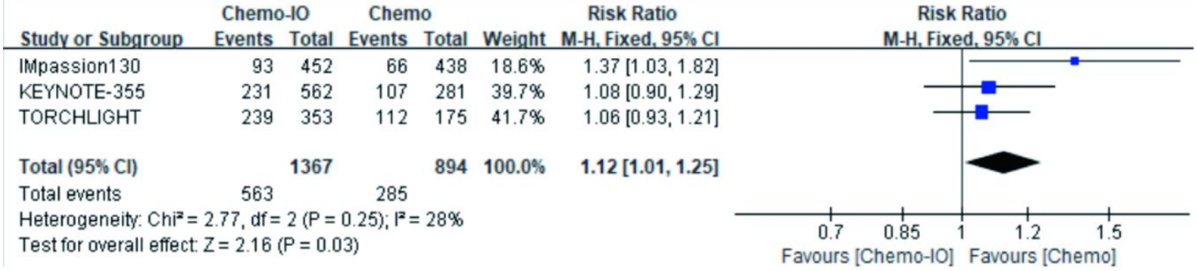

Supplementary Figure S31 Forest plots of the incidence of immune-related adverse events for chemoimmunotherapy versus chemotherapy in mTNBC.

Hypothyroidism

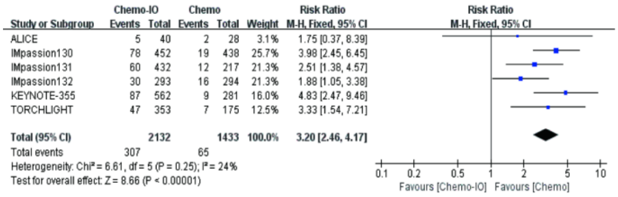

Hyperthyroidism

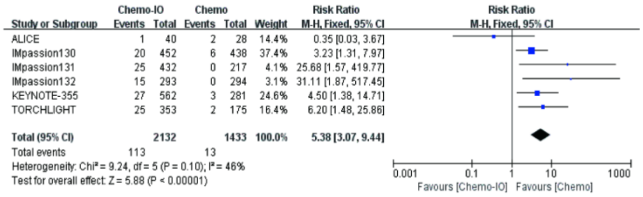

Pneumonitis

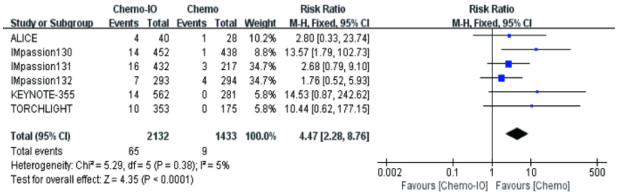

Rash

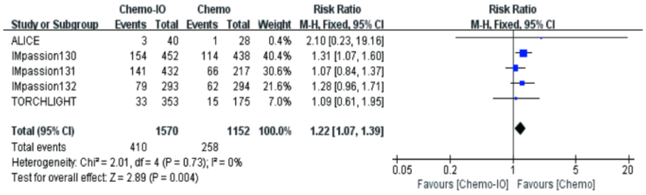

Pyrexia

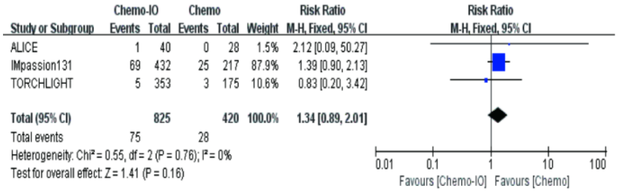

Infusion-related reactions

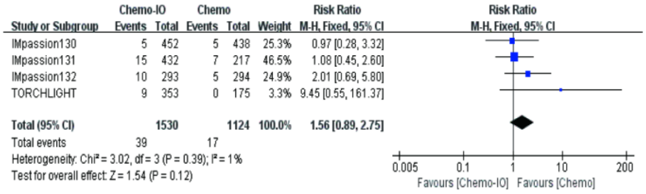

Colitis

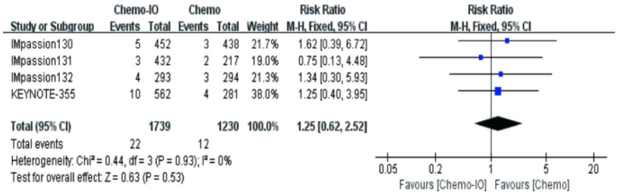

Pancreatitis

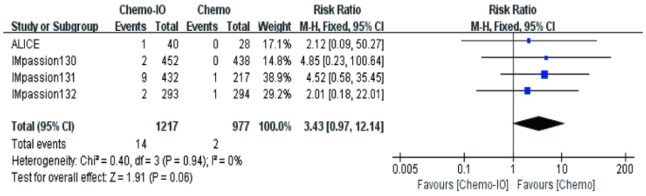

Immune-related diabetes

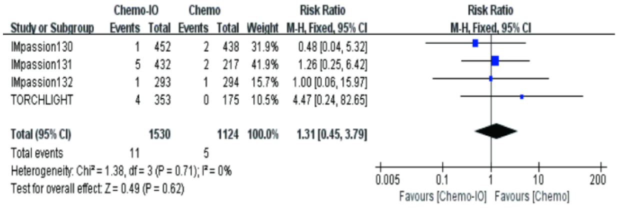

Immune-related hepatitis all types

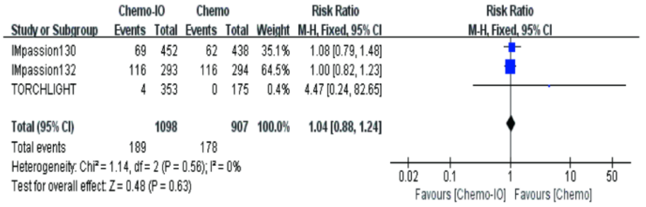

Immune-related hepatitis diagnosis

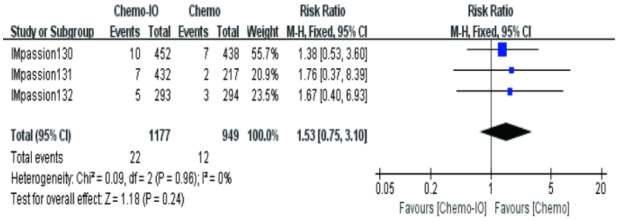

Immune-related adrenal insufficiency

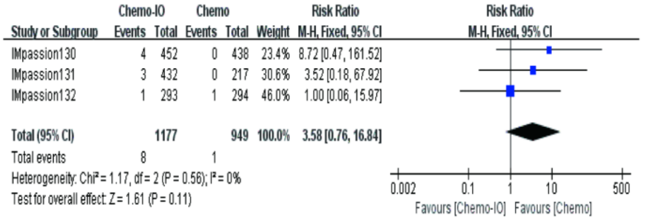

Supplementary Figure S32 Forest plots of the incidence of treatment-emergent adverse events for chemoimmunotherapy versus chemotherapy in mTNBC, adjusted for heterogeneity.

Hyperthyroidism

	Chemo-IO		Chemo			Risk Ratio
Study or Subgroup	Events	Total	Events	Total	Weight	M-H, Fixed, 95% CI
IMpassion130	20	452	6	438	43.7%	3.23 [1.31, 7.97]
IMpassion131	25	432	0	217	4.8%	25.68 [1.57, 419.77]
IMpassion132	15	293	0	294	3.6%	31.11 [1.87, 517.45]
KEYNOTE-355	27	562	3	281	28.7%	4.50 [1.38, 14.71]
TORCHLIGHT	25	353	2	175	19.2%	6.20 [1.48, 25.86]
**Total (95% CI)**		**2092**		**1405**	**100.0%**	**6.23 [3.41, 11.39]**
Total events	112		11			

Heterogeneity: Chi² = 4.57, df = 4 (P = 0.33); I² = 12%

Test for overall effect: Z = 5.95 (P < 0.00001)

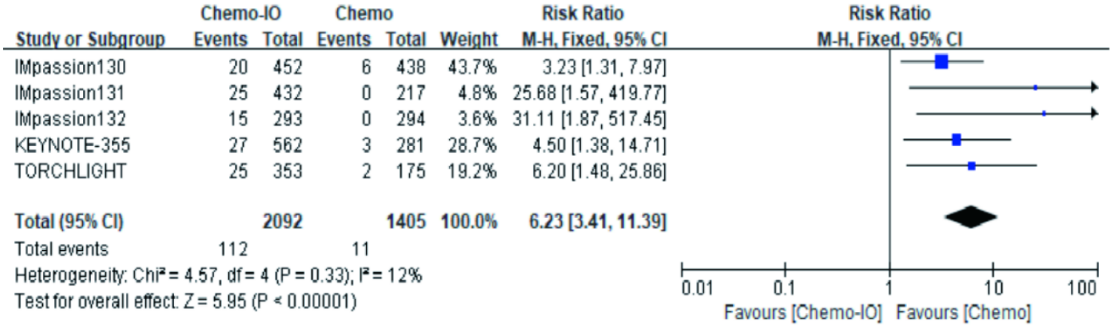

Supplement: Supplementary file 1 [file cancers-18-01352-s001.zip › cancers-4240496-supplementary.pdf]
