# Supplementary material for: PD-L1-Guided Chemo-Immunotherapy in Advanced Triple-Negative Breast Cancer: A Meta-Analysis of Survival Benefits and Toxicity Profiles
Source: Cancers (Basel). 2026 Apr 23;18(9):1352. doi: 10.3390/cancers18091352 (PMC13162691; doi:10.3390/cancers18091352)
Supplement: Supplementary file 1 [file cancers-18-01352-s001.zip › Supplementary File 1 PRISMA_2020_checklist.pdf]

PRISMA 2020 Checklist

Section and Topic	Item #	Checklist item	Location where item is reported
**TITLE**			
Title	1	Identify the report as a systematic review.	Page 1, lines 1–4.
**ABSTRACT**			
Abstract	2	See the PRISMA 2020 for Abstracts checklist.	Pages 1-2, lines 33–72.
**INTRODUCTION**			
Rationale	3	Describe the rationale for the review in the context of existing knowledge.	Pages 2-3, lines 75–101.
Objectives	4	Provide an explicit statement of the objective(s) or question(s) the review addresses.	Page 3, lines 101-103.
**METHODS**			
Eligibility criteria	5	Specify the inclusion and exclusion criteria for the review and how studies were grouped for the syntheses.	Page 3, lines 122–129.
Information sources	6	Specify all databases, registers, websites, organisations, reference lists and other sources searched or consulted to identify studies. Specify the date when each source was last searched or consulted.	Page 3, lines 110–129.
Search strategy	7	Present the full search strategies for all databases, registers and websites, including any filters and limits used.	Table S1
Selection process	8	Specify the methods used to decide whether a study met the inclusion criteria of the review, including how many reviewers screened each record and each report retrieved, whether they worked independently, and if applicable, details of automation tools used in the process.	Page 3, lines 119-129.
Data collection process	9	Specify the methods used to collect data from reports, including how many reviewers collected data from each report, whether they worked independently, any processes for obtaining or confirming data from study investigators, and if applicable, details of automation tools used in the process.	Page 3-4, lines 131-141.
Data items	10a	List and define all outcomes for which data were sought. Specify whether all results that were compatible with each outcome domain in each study were sought (e.g. for all measures, time points, analyses), and if not, the methods used to decide which results to collect.	Page 3, lines 126–129.
	10b	List and define all other variables for which data were sought (e.g. participant and intervention characteristics, funding sources). Describe any assumptions made about any missing or unclear information.	Page 3-4, lines 135-138.
Study risk of bias assessment	11	Specify the methods used to assess risk of bias in the included studies, including details of the tool(s) used, how many reviewers assessed each study and whether they worked independently, and if applicable, details of automation tools used in the process.	Page 4, lines 138-141.
Effect measures	12	Specify for each outcome the effect measure(s) (e.g. risk ratio, mean difference) used in the synthesis or presentation of results.	Page 4, lines 143-148.
Synthesis	13a	Describe the processes used to decide which studies were eligible for each synthesis (e.g. tabulating the study intervention characteristics	Page 3, lines

PRISMA

PRISMA 2020 Checklist

Section and Topic	Item #	Checklist item	Location where item is reported
methods		and comparing against the planned groups for each synthesis (item #5)).	122-125.
	13b	Describe any methods required to prepare the data for presentation or synthesis, such as handling of missing summary statistics, or data conversions.	Page 3, lines 115-118.
	13c	Describe any methods used to tabulate or visually display results of individual studies and syntheses.	Page 4, lines 151-159.
	13d	Describe any methods used to synthesize results and provide a rationale for the choice(s). If meta-analysis was performed, describe the model(s), method(s) to identify the presence and extent of statistical heterogeneity, and software package(s) used.	Page 4, lines 151-159.
	13e	Describe any methods used to explore possible causes of heterogeneity among study results (e.g. subgroup analysis, meta-regression).	Page 4, lines 148-150.
	13f	Describe any sensitivity analyses conducted to assess robustness of the synthesized results.	Page 4, lines 153-154.
Reporting bias assessment	14	Describe any methods used to assess risk of bias due to missing results in a synthesis (arising from reporting biases).	Page 4, lines 138-141.
Certainty assessment	15	Describe any methods used to assess certainty (or confidence) in the body of evidence for an outcome.	Page 4, lines 147-159.
**RESULTS**			
Study selection	16a	Describe the results of the search and selection process, from the number of records identified in the search to the number of studies included in the review, ideally using a flow diagram.	Page 4-5, lines 161-147.
	16b	Cite studies that might appear to meet the inclusion criteria, but which were excluded, and explain why they were excluded.	Page 5, Figure 1.
Study characteristics	17	Cite each included study and present its characteristics.	Page 5, lines 176-194, Table 1 and Tables S2-6.
Risk of bias in studies	18	Present assessments of risk of bias for each included study.	Page 5, lines 165-171, Figure S1.
Results of individual studies	19	For all outcomes, present, for each study: (a) summary statistics for each group (where appropriate) and (b) an effect estimate and its precision (e.g. confidence/credible interval), ideally using structured tables or plots.	Figure S2, 3, 7, 8, 14, 20, 22, 25, 28, 29, 30,

PRISMA

PRISMA 2020 Checklist

Section and Topic	Item #	Checklist item	Location where item is reported
			31, 32.
Results of syntheses	20a	For each synthesis, briefly summarise the characteristics and risk of bias among contributing studies.	Page 4, lines 161-171.
	20b	Present results of all statistical syntheses conducted. If meta-analysis was done, present for each the summary estimate and its precision (e.g. confidence/credible interval) and measures of statistical heterogeneity. If comparing groups, describe the direction of the effect.	Page 6-9, lines 198-291, Figure 2-4, table 7-22, Figure S2-S32.
	20c	Present results of all investigations of possible causes of heterogeneity among study results.	Page 6-7, lines 215-216.
	20d	Present results of all sensitivity analyses conducted to assess the robustness of the synthesized results.	Page 7-9, lines 238-241, lines 245-249, lines 269-271, Figure S9-11, 14-17, 23-25.
Reporting biases	21	Present assessments of risk of bias due to missing results (arising from reporting biases) for each synthesis assessed.	Page 6-7, lines 215-219, lines 241-242, lines 255-256, lines 272-273, Table S8, 9-14, 18, 20, S22; Figure 4-6, 18, 21, 26.
Certainty of evidence	22	Present assessments of certainty (or confidence) in the body of evidence for each outcome assessed.	Figure2-4, FigureS2-3, 7-8, 14, 20, 22, 25, 28, 29, 30, 31, 32.
**DISCUSSION**			
Discussion	23a	Provide a general interpretation of the results in the context of other evidence.	Pages 9, lines

PRISMA 2020 Checklist

Section and Topic	Item #	Checklist item	Location where item is reported
			293-299.
	23b	Discuss any limitations of the evidence included in the review.	Pages 10, lines 346-356, lines 363-367,
	23c	Discuss any limitations of the review processes used.	Pages 12, line 440.
	23d	Discuss implications of the results for practice, policy, and future research.	Pages 9-12, lines 306-308, lines 314-316, lines 337-339, lines 354-356, lines 375-378, lines 413-414, lines 434-439.
**OTHER INFORMATION**			
Registration and protocol	24a	Provide registration information for the review, including register name and registration number, or state that the review was not registered.	Page 3, lines 106-108.
	24b	Indicate where the review protocol can be accessed, or state that a protocol was not prepared.	Page 13, lines 479-481.
	24c	Describe and explain any amendments to information provided at registration or in the protocol.	Not reported in the manuscript.
Support	25	Describe sources of financial or non-financial support for the review, and the role of the funders or sponsors in the review.	Page 12, lines 467-468.
Competing interests	26	Declare any competing interests of review authors.	Page 13, lines 469-472.
Availability of data, code and other materials	27	Report which of the following are publicly available and where they can be found: template data collection forms; data extracted from included studies; data used for all analyses; analytic code; any other materials used in the review.	Page 13, lines 479-481.

From: Page MJ, McKenzie JE, Bossuyt PM, Boutron I, Hoffmann TC, Mulrow CD, et al. The PRISMA 2020 statement: an updated guideline for reporting systematic reviews. BMJ 2021;372:n71. doi: 10.1136/bmj.n71. This work is licensed under CC BY 4.0. To view a copy of this license, visit https://creativecommons.org/licenses/by/4.0/
